# Synthesis of π-Extended Thiele’s and Chichibabin’s Hydrocarbons and Effect of the π-Congestion on Conformations and Electronic States

**DOI:** 10.1021/jacs.2c02318

**Published:** 2022-04-15

**Authors:** Tomohiko Nishiuchi, Seito Aibara, Hiroyasu Sato, Takashi Kubo

**Affiliations:** † Department of Chemistry, Graduate School of Science, Osaka University, 1-1 Machikaneyama, Toyonaka, Osaka 560-0043, Japan; ‡ Innovative Catalysis Science Division, Institute for Open and Transdisciplinary Research Initiatives, (ICS-OTRI), Osaka University, Suita, Osaka 565-0871, Japan; § Rigaku Corporation, 3-9-12 Matsubara, Akishima, Tokyo 196-8666, Japan

## Abstract

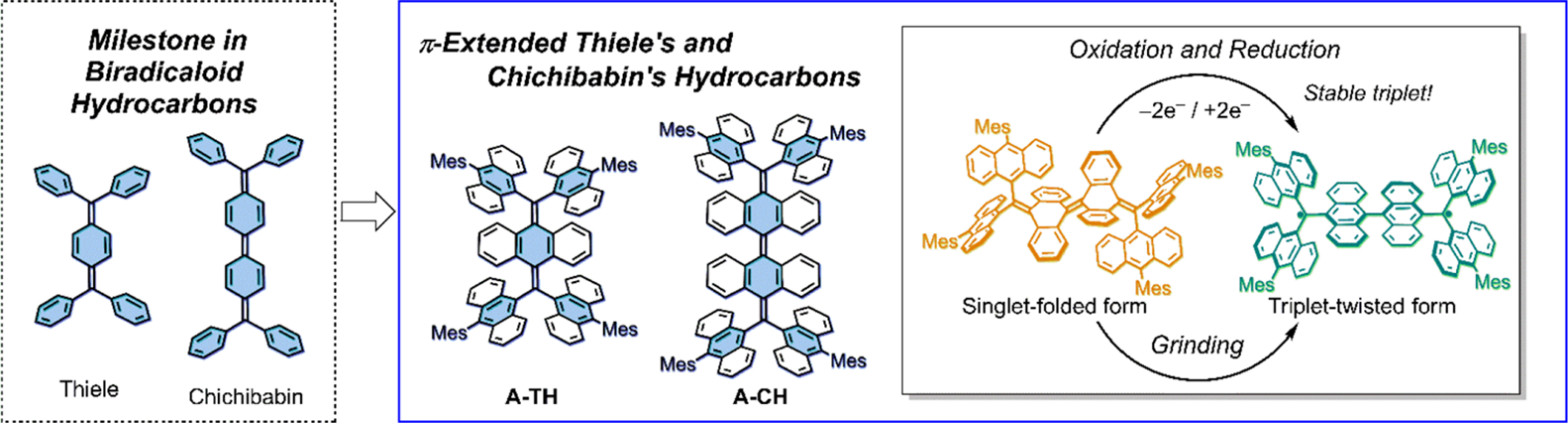

The biradicaloid of Chichibabin’s hydrocarbon exits in a unique thermal equilibrium between closed-shell singlet and open-shell triplet forms. Conceptually, the incorporation of nonplanar aromatic groups, such as anthraquinodimethane (AQD), in these species could bring about stabilization of the individual singlet and triplet spin biradicaloids by creating a high energy barrier for conformational interconversion between folded (singlet) and twisted (triplet) forms. Moreover, this alteration could introduce the possibility of controlling spin states through conformational changes induced by chemical and physical processes. Herein, we report the preparation of AQD-containing, π-extended Thiele’s (**A-TH**) and Chichibabin’s (**A-CH**) hydrocarbons, which have highly π-congested structures resulting from the presence of bulky 9-anthryl units. The π-congestion in these substances leads to steric frustration about carbon–carbon double bonds and creates flexible dynamic motion with a moderate activation barrier between folded singlet and twisted triplet states. These constraints make it possible to isolate the twisted triplet state of **A-CH**. In addition, simple mechanical grinding of the folded singlet of **A-CH** produces the twisted triplet.

## Introduction

**Figure 1:**
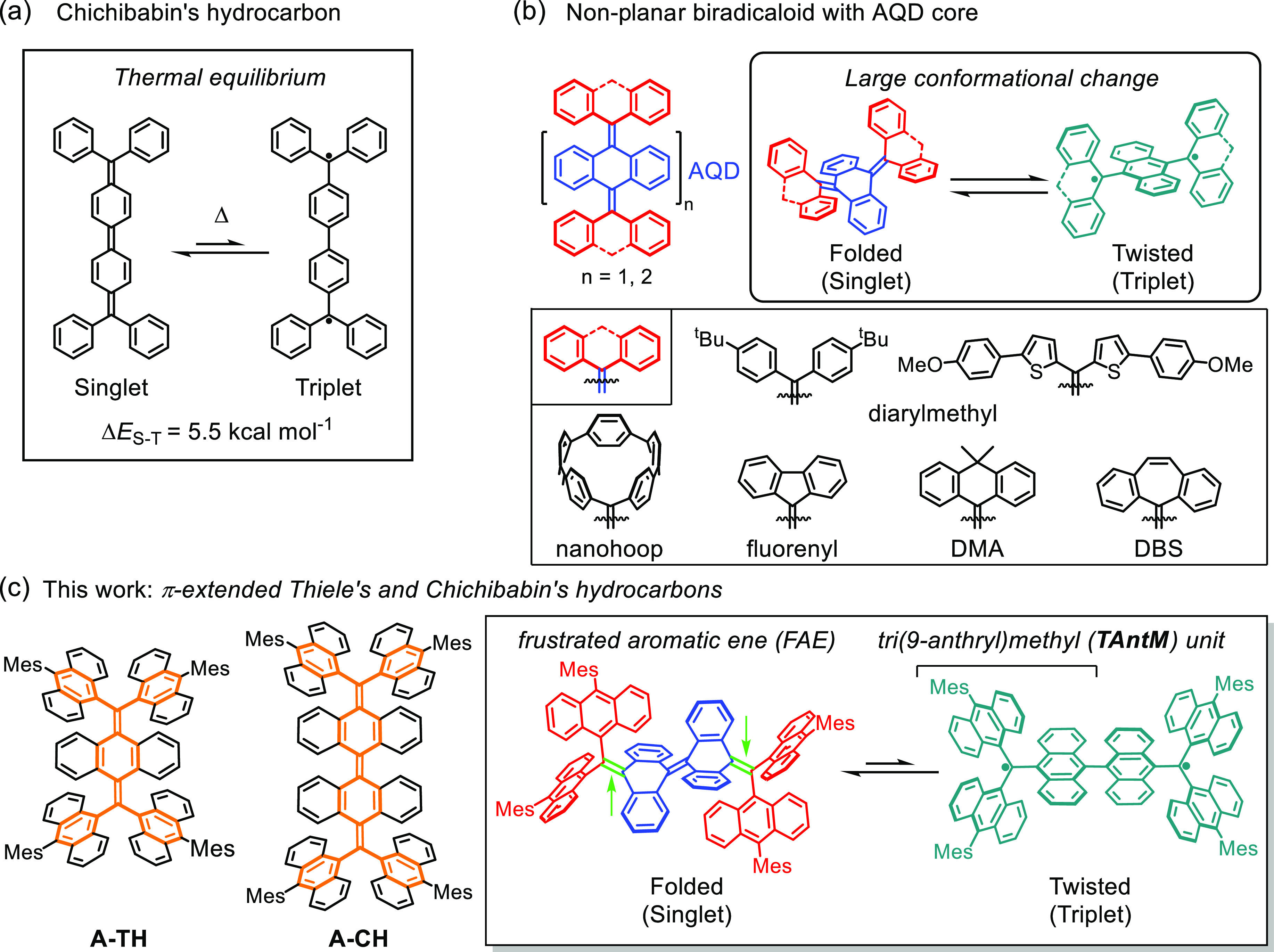
(a) Structure of Chichibabin’s hydrocarbon and its small Δ*E*_S-T_. (b) Basic structure and recent examples of nonplanar biradicaloids with the AQD core. (c) Structures of π-extended Thiele’s and Chichibabin’s hydrocarbons **A-TH** and **A-CH** (left), and the structural features in this system (right).Green arrows indicate the parts of FAE.

In 1986, Montgomery and co-workers solved the long-standing paradox about the nature of Chichibabin’s hydrocarbon (CH),^1,2^ which is composed of two triphenylmethyl (trityl) radical units (Figure 1a).^3,4^ Magnetic susceptibility measurements showed that this hydrocarbon exists in a singlet spin state^5^ and that no thermally excited triplet state signal but only a doublet signal is observed in the electron spin resonance (ESR) spectrum.^6−9^ This phenomenon was explained by using the concept that the hydrocarbon is a “biradicaloid,” having a structure in which two unpaired electrons weakly interact through an almost planar quinoid skeleton. This property leads to thermal equilibrium between singlet and triplet states that are separated by a small energy difference (Δ*E*_S-T_) of −5.5 kcal mol^–1^, and unusual carbon–carbon distances that are halfway between single and double bond values (Figure 1a). Based on this seminal effort, many aromatic biradicaloids have been synthesized and their unique optical and magnetic properties have been elucidated.^10−23^ Now, more than 35 years after Montgomery’s achievement, the chemistry of biradicaloids still attracts great attention.

Recently, the concept of biradicaloid has been extended to nonplanar aromatic systems such as overcrowded ethylenes (OCEs).^24−28^ While planar biradicaloids undergo rapid thermal equilibrium between singlet and triplet states in the absence of an attending conformational change, nonplanar biradicaloids embedded with anthraquinodimethane (AQD) groups undergo large conformational changes between folded closed-shell singlet and twisted open-shell triplet forms. Wu,^29,30^ Campos,^31^ Suzuki,^32,33^ Campaña,^34^ Sun,^35^ and our group^36^ have described various AQD-based nonplanar biradicaloids, which incorporate diarylmethyl, fluorenyl, dihydrodimethylanthryl, dibenzosuberenyl, and nanohoop units (Figure 1b). The most important feature of diradicaloids in which interconversions between singlet and triplets states are associated with large conformational changes is that singlet–triplet interconversion can be controlled and the triplet state is potentially isolable. However, experimental detection of the triplets, for example, by using ESR spectroscopy, is often unsuccessful in these systems because the triplet state conformer has a much higher energy than the singlet state conformer and the thermal activation barrier for the interconversion is low. Only Suzuki^33^ and our group^36^ have gained definitive evidence of triplet states through detection of zero-field splitting in ESR spectra of species generated by trapping in a matrix after heating and by photoisomerization in solution, respectively. However, isolation of triplet state conformers under ambient conditions and the observation of conformational switching between singlet and triplet state forms remain challenging.

In the study described below, we have designed a new family of AQD-based nonplanar biradicaloids, which possess per-dibenzo π-extended forms of Thiele’s (**A-TH**) and Chichibabin’s (**A-CH**) hydrocarbon (Figure 1c).

Introduction of di(9-anthryl)methyl units at the terminal sp^2^ hybridized carbons of the AQD core in each system gives rise to a highly congested π-system comprising two tri(9-anthryl)methyl (**TAntM**) radical units.^37,38^ As a result, highly stable triplet states are expected in the twisted conformers. In contrast, the folded conformers of these substances contain unique OCEs on the terminal C=C bonds, which give rise to sterically frustrated aromatic enes (FAEs), and the coexistence of both folded and twisted C=C bonds.^39^ In the current effort, we observed that π-congestion associated with the anthryl units present in **A-TH** and **A-CH** gives rise to unique dynamic behavior in solution. We also observed that **A-TH** can be isolated in the solid state and shown to exist in an unusual *anti*-conformation about the AQD core, which represents an intermediate in the conformational conversion between *syn*-conformations. Importantly, the triplet state of **A-CH** can be isolated and unambiguously characterized by using UV–vis–NIR and ESR spectroscopy. Furthermore, owing to the presence of flexible FAEs, the structural change of **A-CH** from a folded conformer (singlet state) to a twisted conformer (triplet state) can be promoted in the solid state by simple mechanical grinding. The unique characteristics of these new biradicaloids were also revealed by using computational approaches.

## Results and Discussion

### Synthesis and Dynamic Behavior in Solution

**Scheme 1:**
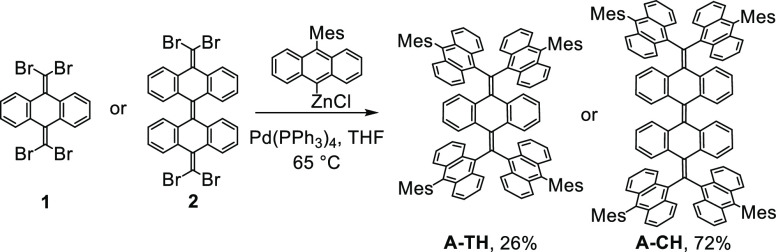
Route for Synthesis of **A-TH** and **A-CH**

The methods used to synthesize **A-TH** and **A-CH** are shown in Scheme 1. It is notable that even though the presence of 9-anthryl groups creates highly congested environments, these substances are generated with modest efficiencies through one-step Negishi coupling reactions of the perbrominated starting materials **1** and **2** with mesityl-substituted anthryl zinc chloride (26% for **A-TH** as a red solid or in 72% for **A-CH** as a yellow solid).

**Figure 2:**
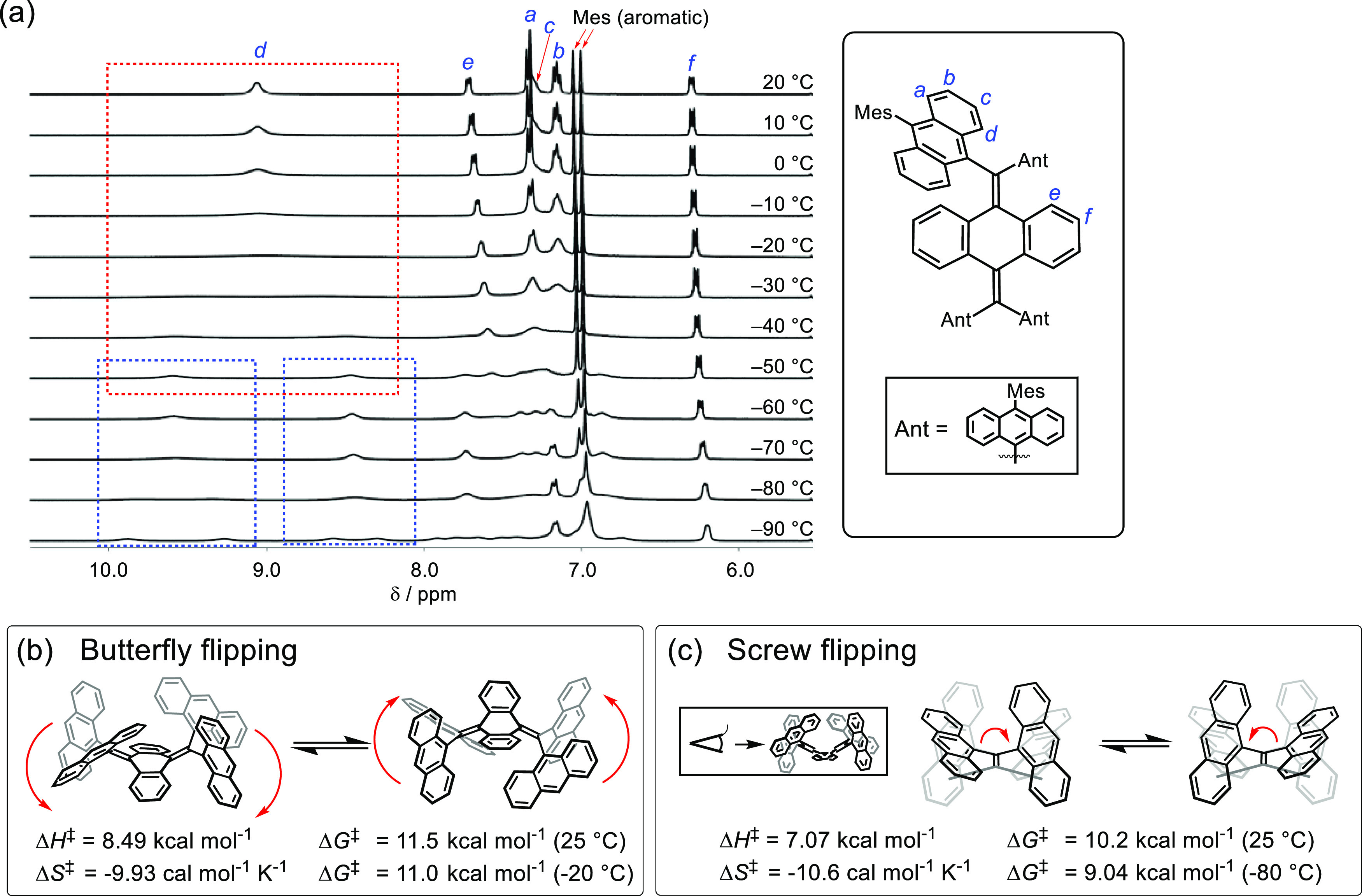
(a) VT ^1^H-NMR chart of **A-TH** from 20 to −90 °C in CD_2_Cl_2_. Large coalescence was observed at *d* protons. The first coalescence occurs from 20 to −50 °C (red dashed square), and the second coalescence occurs from −50 to −90 °C (blue dashed squares). (b) Dynamic motion of butterfly flipping in the AQD unit corresponding to the first coalescence and experimentally determined thermodynamic parameters. (c) Dynamic motion of screw flipping in the AQD unit observed from the long-axis direction, corresponding to the second coalescence and experimentally determined thermodynamic parameters. For (b) and (c), Mes substitution at anthryl units of **A-TH** is omitted for clarity.

**Figure 3:**
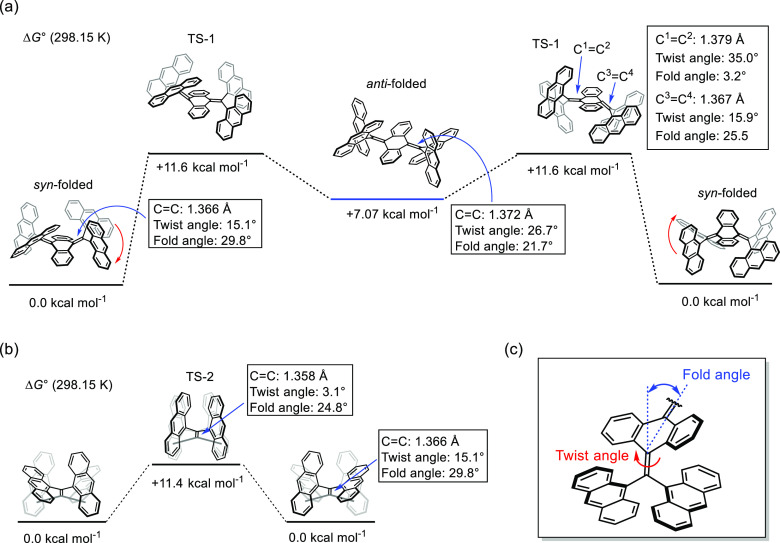
Energy diagrams for the structural interconversion of **A-TH′** and structural parameters such as the C–C bond length and its twist and fold angles at the ωB97X-D/6-31G** level of theory. For the calculations, Mes substitution at anthryl units of **A-TH** is omitted. (a) Energy diagram for the butterfly flipping of **A-TH′**. (b) Energy diagram for the screw flipping of **A-TH′**. (c) Definition of twist and fold angles.

The ^1^H-NMR signals of several known AQD-based biradicaloids gradually broaden with the increasing temperature probably because the population of triplet state species increases at higher temperatures.^40,41^ Thus, it is noteworthy that signals in the solution-phase ^1^H-NMR spectra of **A-TH** and **A-CH** display the opposite behavior. Specifically, the broad ^1^H-NMR signals at 25 °C become sharper upon heating to 80 °C (Figures S1 and S2). This behavior indicates that a slow conformational change takes place at ambient temperatures and no triplet species exist at high temperatures. To evaluate this dynamic behavior in solution, low-temperature VT ^1^H-NMR experiments were conducted. In Figure 2a, are shown the low magnetic field (5.5–10.5 ppm) regions of ^1^H-NMR spectra of **A-TH** at temperatures between −90 and 20 °C. The positions of protons for **A-TH** were assigned by using 2D-NMR (Figures S3 and S4). Inspection of the spectra shows that upon cooling the broad signal for anthracene ring proton *d* at 9.1 ppm broadens and at −40 °C separates into two signals at 8.5 and 9.6 ppm. In addition, the two newly formed peaks separate further at −90 °C. The VT-NMR results indicate that two dynamic motions of **A-TH** occur in solution. The thermodynamic parameters (Δ*H*^‡^, Δ*S*^‡^, and Δ*G*^‡^) for these motions determined by using curve-fitting analysis of the signals for proton *d* (Figure S5) are given in Figure 2b,c. The dynamic motion corresponding to the first ^1^H-NMR peak coalescence is associated with butterfly flipping of the AQD core, and the second coalescence corresponds to screw flipping of the di(9-anthryl)methyl units around the C=C bond. The Δ*G*^‡^ values associated with these respective processes were determined to be 11.0 kcal mol^–1^ (−20 °C, *T*_C_) and 9.04 kcal mol^–1^ (−80 °C, *T*_C_). To gain deeper insight, density functional theory (DFT) calculations for this structural change were also performed using **A-TH′** in which the Mes substituents are absent (Figure 3). The results show that the Δ*G*^‡^ value to reach the stepwise pathway for butterfly flipping is +11.6 kcal mol^–1^, which is in good agreement with the experimental result.^42^ Also, the *anti*-folded conformer has an energy of +7.07 kcal mol^–1^, which is higher than that of the *syn*-folded conformer, and the activation barrier to the *syn*-folded conformer is 4.53 kcal mol^–1^.

Therefore, in solution, detection of the *anti*-folded conformer is not possible by using NMR techniques owing to its very low population caused by the rapidly occurring conformational change. For screw flipping through TS-2, the Δ*G*^‡^ value was found to be +11.4 kcal mol^–1^, which is also consistent with the experimental result. In the calculated *syn*-folded conformer, a CH−π interaction exists between adjacent large anthryl units (Figure S6). This interaction stabilizes the *syn*-folded conformer, resulting in an increase in the activation barrier for screw flipping.

In contrast, low-temperature VT-NMR results for **A-CH** are highly complicated owing to the high flexibility of the system in solution. Although signal broadening was observed, quantitative determination of the thermodynamic parameters is difficult due to the presence of many overlapping broad signals (Figure S7). However, as discussed below, we can conclude that the change from the folded to the twisted conformer does not occur at low temperatures due to the high activation barrier.

### UV–vis, X-Ray Structure, CV, and ESR Spectra

**Figure 4:**
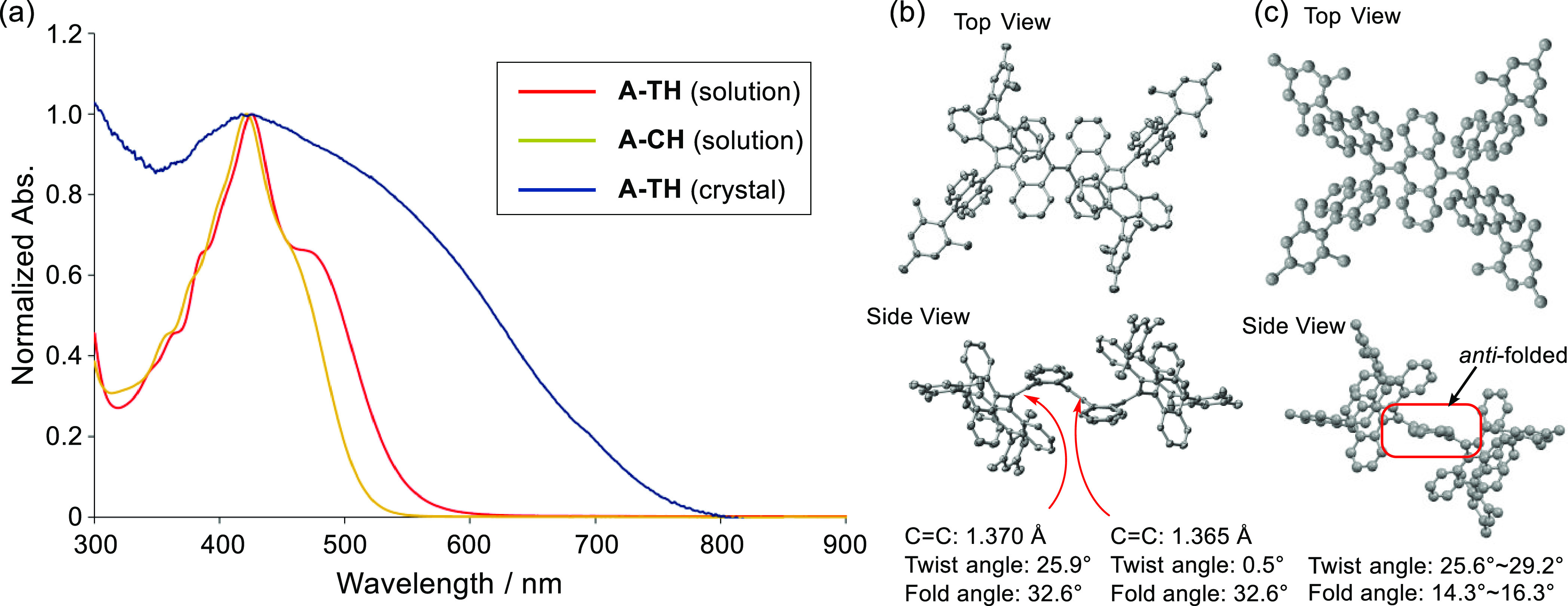
(a) Normalized UV–vis spectra of **A-TH** and **A-CH** (CH_2_Cl_2_ solution) as well as **A-TH** (crystalline state). (b) X-ray crystallographic structure of **A-CH**. Top view (up) and side view (down) with the bond length, twist angle, and fold angle of the C=C bond. (c) X-ray crystallographic structure of **A-TH**. Top view (up) and side view (down). Protons are omitted for clarity.

The UV–vis spectra of **A-TH** and **A-CH**, displayed in Figure 4, contain intense absorption bands with respective maxima at 425 nm (ε = 4.12 × 10^4^ cm^–1^ M^–1^) and 420 nm (ε = 5.05 × 10^4^ cm^–1^ M^–1^). Even though the structure of **A-CH** contains a more extended π-system than **A-TH** does, its absorption-edge occurs at a shorter wavelength, which is confirmed using time-dependent (TD)-DFT calculations (Figures S8 and S12).

The structures of **A-TH** and **A-CH** in the crystalline state were determined by using X-ray crystallographic analysis. In agreement with DFT calculations, the crystalline state structure of **A-CH** possesses a folded conformation (Figure 4b). The bond lengths of the central and terminal C=C bonds were found to be 1.365 and 1.370 Å, respectively, which are normal. The frustrated aromatic ene (FAE) moiety in **A-CH** contains a highly deformed C=C bond with a large twist angle (25.9°) and folded AQD core (32.6°).

On the other hand, **A-TH** in the crystalline state has an *anti*-folded conformation, which corresponds to the higher energy intermediate in the conformational interconversion (Figure 4c). The reason for this might lie in the presence of intermolecular interactions between the large mesityl-substituted anthryl units gaining large stabilization energy in the crystalline state. The twist and fold angles of C=C bonds in **A-TH** range from 25.6° to 29.2° and 14.3° to 16.3°, respectively.^43^ The unique structure of *anti*-folded **A-TH** in the crystalline state is reflected in its UV–vis absorption spectrum (Figure 4a). Compared with that in solution, the absorption maximum of the crystal is greatly red-shifted with its absorption edge extending to 800 nm. TD-DFT calculations also show that the S_0_ → S_1_ transition of the *anti-*folded conformer is 535 nm (*f* = 0.3736), which is longer than that of the *syn*-folded conformer (497 nm, *f* = 0.1905) (Figure S9).

Although the *anti*-folded conformer might be capable of accessing the thermally excited triplet state, the calculated energy difference between triplet and *anti*-folded conformers of +9.73 kcal mol^–1^ is too high for this to take place under normal experimental conditions (Figure S16).

**Figure 5:**
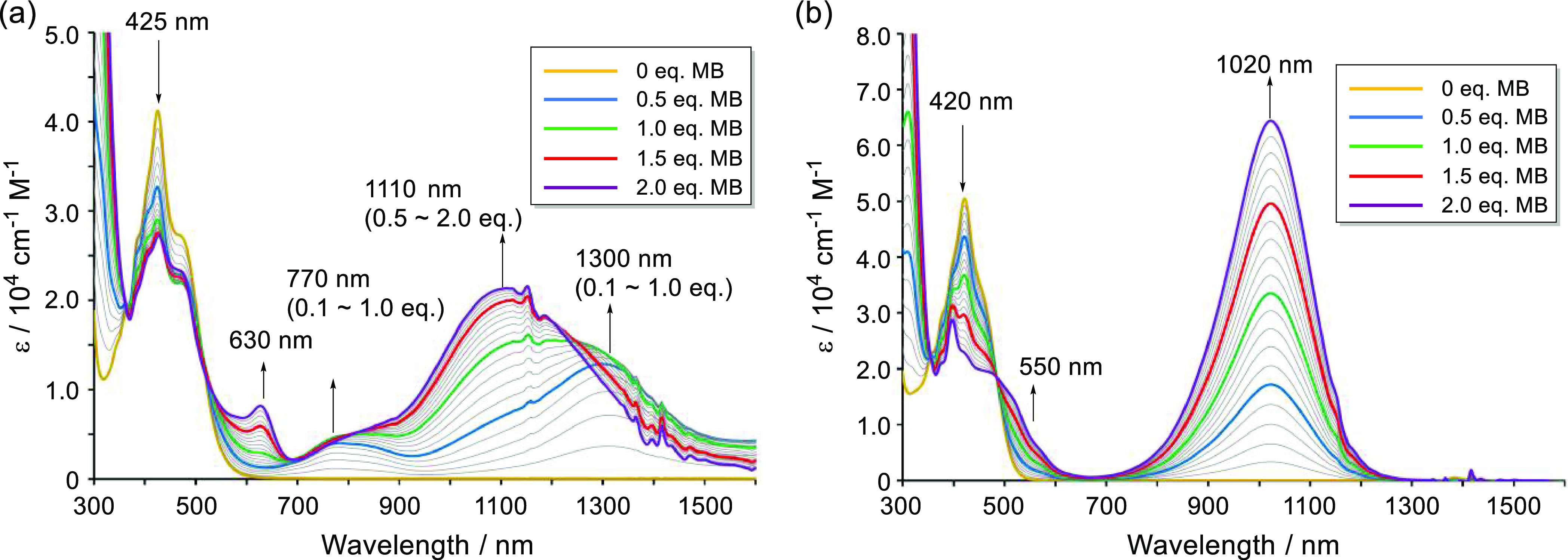
UV–vis–NIR chemical oxidative titration using MB (added at 0.1 equiv each) in CH_2_Cl_2_/MeCN. (a) **A-TH** with MB from 0 to 2.0 equiv. (b) **A-CH** with MB from 0 to 2.0 equiv.

Next, UV–vis–NIR oxidative titration of **A-TH** and **A-CH** using tris(4-bromophenyl)ammoniumyl hexachloroantimonate (magic blue, MB) was performed to generate and investigate their cation states. When 0.1–0.5 equiv of MB in MeCN solution is added to **A-TH** in CH_2_Cl_2_, a broad absorption peak at 1300 nm with a shoulder at 1110 nm forms (Figure 5a). Upon increasing the amount of MB relative to **A-TH**, the broad peak gradually shifts to a shorter wavelength and then an intense peak at 1110 nm arises after addition of 2.0 equiv of MB. The results are in accord with the expectation that radical cation **A-TH**^·^^+^ (λ_abs_ = 1300 nm) is initially formed together with a small amount of dication **A-TH**^2+^ (λ_abs_ = 1110 nm) and that the amount of dication **A-TH**^2+^ increases with increasing amounts of the oxidant MB. The absorption properties of the monocation and dication are well reproduced by using TD-DFT calculations (Figures S10 and S11). In the case of **A-CH**, addition of increasing amounts of MB only results in gradual formation of an intense broad peak at 1020 nm (Figure 5b), which is similar to that in the spectrum of the **TAntM** cation (λ_abs_ = 990 nm, Figure S17). Therefore, chemical oxidation of **A-CH** involves a disproportionation reaction of the intermediate radical cation **A-CH**^·^^+^, which directly produces dication **A-CH**^2+^. This is likely the result of the fact that the oxidation potential of radical cation **A-CH**^·^^+^ is lower than that of the folded **A-CH** (see below).

**Figure 6:**
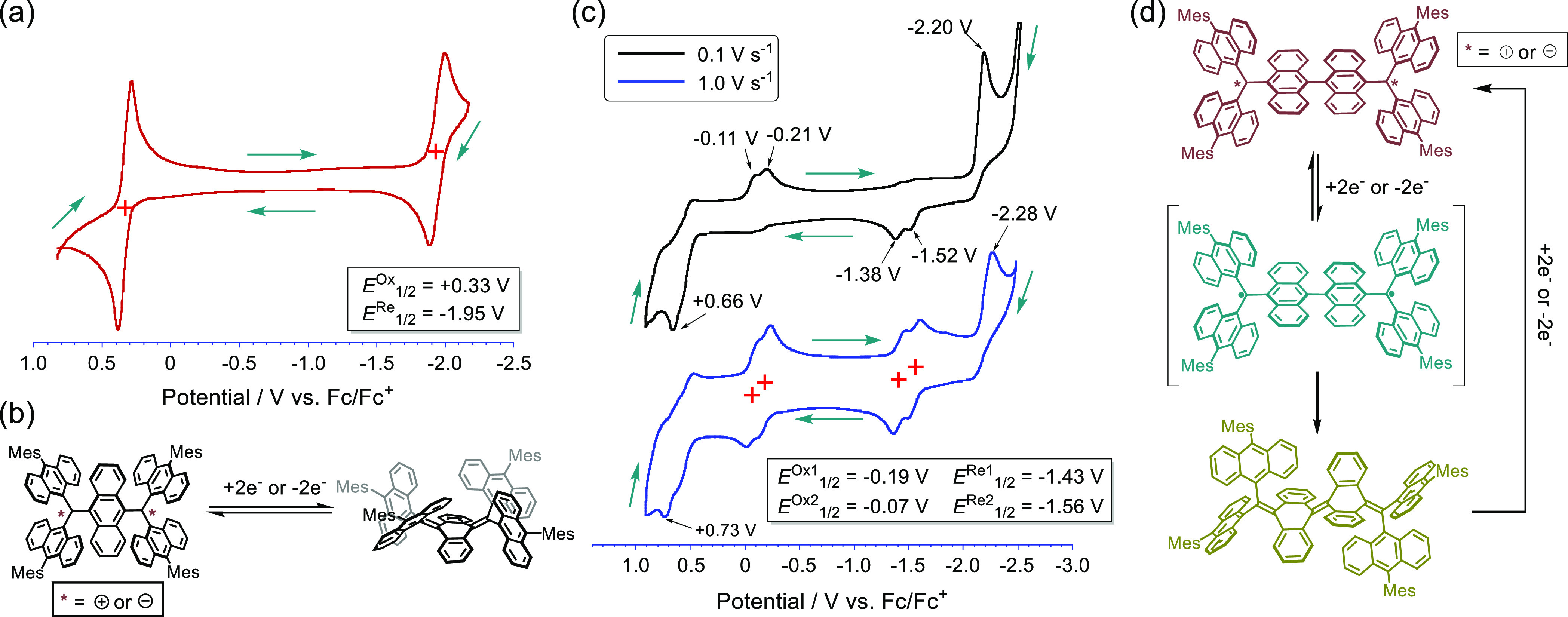
(a) CV of **A-TH** (0.1 M ^n^Bu_4_NPF_6_ in CH_2_Cl_2_, scan rate = 0.1 V s^–1^). Green arrows indicate the scan direction. (b) Conformational change of **A-TH** during the oxidation/reduction processes. (c) CV of **A-CH** (0.1 M ^n^Bu_4_NPF_6_ in CH_2_Cl_2_). Scan rate = 0.1 V s^–1^ (black line) and 1.0 V s^–1^ (blue line). Green arrows indicate the scan direction. (d) Conformational change of **A-CH** during the oxidation/reduction processes.

Cyclic voltammograms (CV) of **A-TH** and **A-CH** were measured to assess whether triplet states are generated during the redox processes. The CV of **A-TH** contains reversible oxidation/reduction waves *E*^Ox^_1/2_ = +0.34 V and *E*^Re^_1/2_ = −1.90 V (vs Fc/Fc^+^) (Figure 6a). This observation indicates that **A-TH** participates in one-step two-electron oxidation/reduction processes, and thus, the twisted dication **A-TH**^2+^ or dianion **A-TH**^2–^ conformer readily reverts to the *syn*-folded neutral counterpart (Figure 6b). Differing from the oxidative titration results described above, a stepwise oxidation process via radical cation species does not take place. This phenomenon is probably due to small on-site coulomb repulsion. The CV of **A-CH** contains irreversible oxidation/reduction waves at −0.1 to −0.2 V and −1.4 to −1.5 V (Figure 6c). It is noteworthy that increasing the scan rate from 0.1 to 1.0 V s^–1^ reduces the irreversibility, resulting in a CV that corresponds to a reversible two-step two-electron oxidation/reduction process. Furthermore, the reversible oxidation and reduction potentials (*E*^Ox^_1/2_ = −0.19 and −0.07 V; *E*^Re^_1/2_ = −1.43 and −1.56 V) are very close to those of the **TAntM** radical (*E*^Ox^_1/2_ = −0.19; *E*^Re^_1/2_ = −1.48 V). Therefore, different from that of **A-TH**, the twisted dication **A-CH**^2+^ or dianion **A-CH**^2–^ first forms the twisted biradical **A-CH**^2^^·^ upon reduction or oxidation, indicating that the activation barrier for conversion of the twisted to the folded conformer is higher in **A-CH** than in **A-TH**. (Figure 6d).

**Figure 7:**
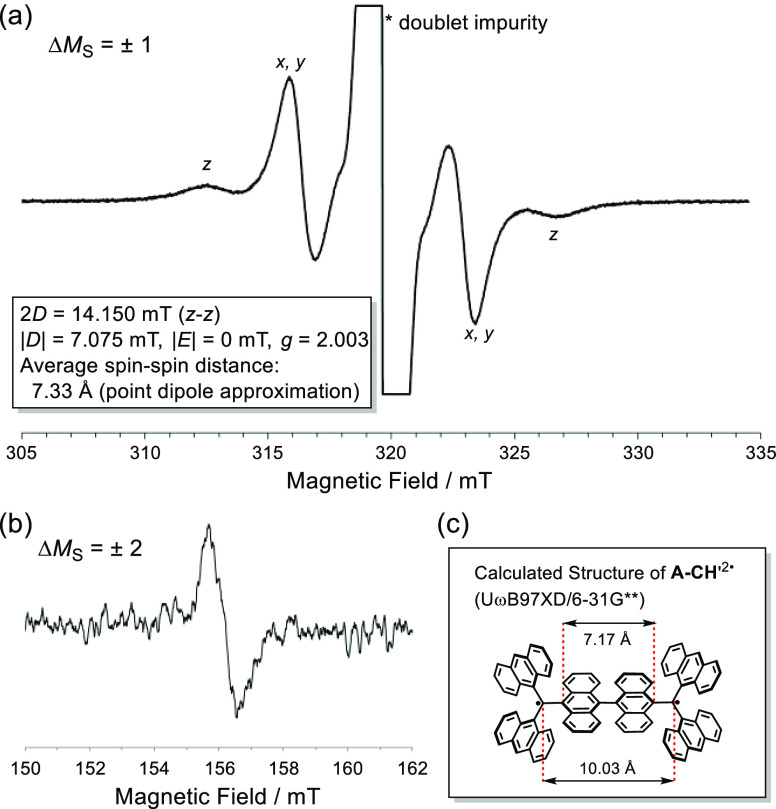
(a) ESR spectrum of **A-CH**^2^^·^ (Δ*M*_S_ = ±1) in toluene at −73 °C. (b) Forbidden half-field transition (Δ*M*_S_ = ±2) of **A-CH**^2^^·^. (c) Calculated carbon–carbon distances between sp^2^ carbons and 10,10′-positions of the central dianthryl unit.

**Scheme 2:**
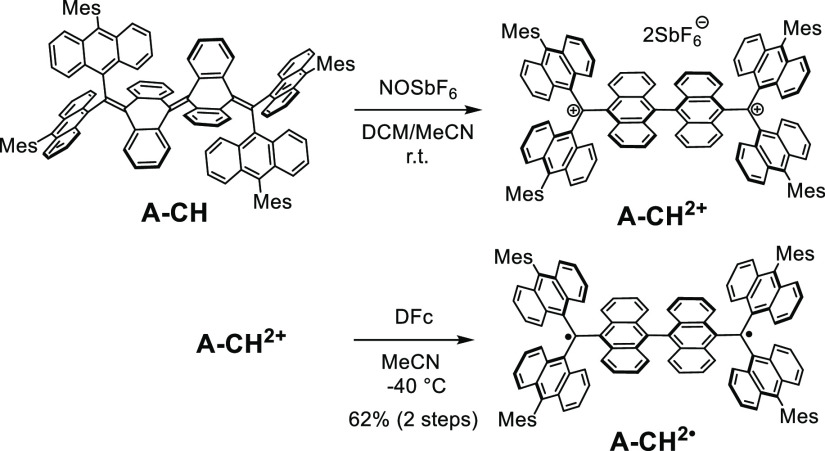
Route for Synthesis of **A-CH**^2·^

These experimental results suggest that, while isolating **A-TH** in its twisted triplet state will be difficult, it might be possible to form and isolate the twisted triplet **A-CH**^2^^·^ by using reduction of the twisted dication **A-CH**^2+^. To explore this possibility, reduction of **A-CH**^2+^ using decamethyl ferrocene (DFc) in MeCN was carried out at −40 °C (Scheme 2). This process formed a powder precipitate having a purple-metallic luster that was collected using filtration. The ESR spectrum of the powder in toluene at −73 °C contains a characteristic pattern for Δ*M*_S_ = ±1 peaks associated with triplet species (Figure 7a), along with a forbidden half-field transition for Δ*M*_S_ = ±2 (Figure 7b). Using the point dipole approximation, the average spin–spin distance was estimated to be 7.33 Å, which is shorter than the distance between the spin-center carbons in **A-CH** (10.03 Å) and close to the distance between 10- and 10′-positions of the central dianthryl unit (7.17 Å) (Figures 7c and S18). The findings provide unambiguous evidence for the conclusion that the triplet state of twisted **A-CH**^2^^·^ can be isolated using precipitation at low temperatures.

**Figure 8:**
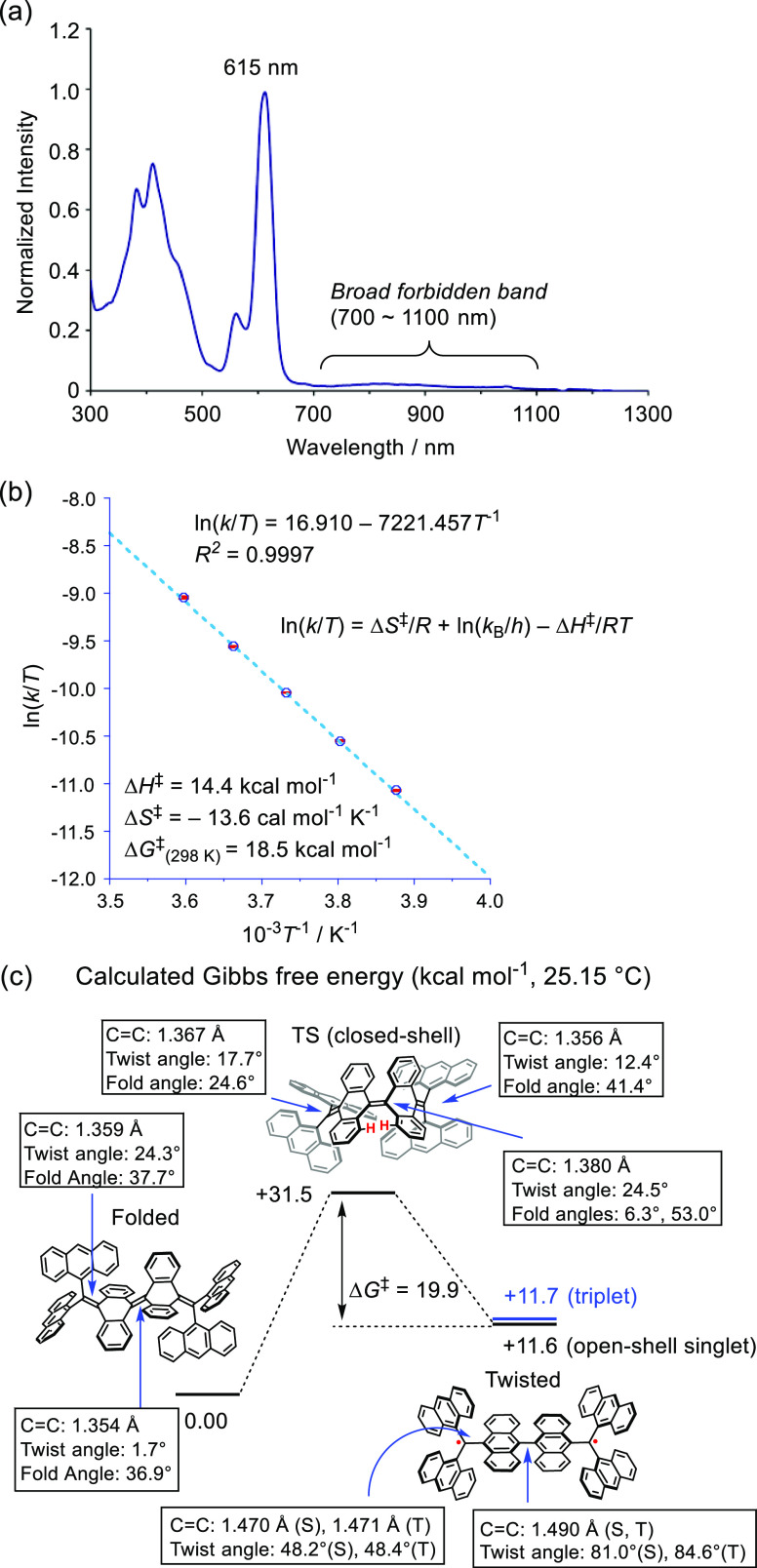
(a) UV–vis–NIR spectrum of twisted **A-CH**^2^^·^ in CH_2_Cl_2_ (at −30 °C). (b) Eyring plot of the structural conversion from twisted **A-CH**^2^^·^ to folded **A-CH**, and its thermodynamic parameters, Δ*H*^‡^, Δ*S*^‡^, and Δ*G*^‡^ (25 °C). (c) Calculated Gibbs free energies of **A-CH′** for the structural conversion, and structural parameters such as the C–C bond length and its twist and fold angles ((U)ωB97X-D/6-31G**). For the twisted conformer, S and T indicate singlet and triplet states, respectively. In the TS state, red protons are the most sterically repulsive positions.

Owing to the orthogonal orientation of the central dianthryl unit, the UV–vis–NIR spectrum (Figure 8a) of triplet **A-CH**^2^^·^ at a low temperature (−30 °C) is almost identical to that of the **TAntM** radical (Figure S17). Specifically, the spectrum contains an intense peak at 615 nm and a broad forbidden band from 700 to 1100 nm. It should be emphasized that the UV–vis–NIR spectrum was measured at a low temperature because the broad band decays quickly at room temperature. To determine thermodynamic parameters for the conversion of triplet **A-CH**^2^^·^ to folded **A-CH** (Figure S19), decay of the band at 615 nm at temperatures including 5, 0, –5, −10, and −15 °C was analyzed. The half-life *t*_1/2_ of **A-CH**^2^^·^ was found to be only 21 s at 5 °C and ca. 3 min at −15 °C. These decay half-lives are considerably shorter than those of the π-extended CH reported by Wu (*t*_1/2_ = 495 min at 25 °C).^30^

The reason for the difference is not totally clear. Related to this issue is the fact that the **TAntM** radical itself displays high flexibility in association with anthryl rotation and unpaired electron migration (Δ*E* = ca. 10 kcal mol^–1^).^37^ Therefore, the fast decay rate of triplet **A-CH**^2^^·^ is consistent with the properties of the **TAntM** radical. In addition, due to the moderate spin distribution on the anthryl units in the triplet state (Figure S18), thermodynamic stabilization of **A-CH**^2^^·^ is also expected. Thus, the triplet **A-CH**^2^^·^ seems to approach the folded **A-CH** conformation in energy, which also leads to a reduction of the interconversion energy barrier. The rate constants derived from averages of three measurements at each temperature were used to construct an Eyring plot, which gave the following thermodynamic parameters for conversion of twisted triplet **A-CH**^2^^·^ to folded **A-CH**: Δ*H*^‡^ = +14.4 kcal mol^–1^, Δ*S*^‡^ = −13.6 cal mol^–1^ K^–1^, and Δ*G*^‡^ = +18.5 kcal mol^–1^ (25 °C) (Figure 8b).

DFT calculations on an **A-CH′** analogue missing Mes substituents were conducted to understand the details of the triplet **A-CH**^2^^·^ decay process (Figure 8c). Compared to that of the folded conformer, the relative Gibbs free energy of the twisted conformer having an open-shell singlet state is calculated to be +11.6 kcal mol^–1^ and its triplet state energy is +11.7 kcal mol^–1^. Thus, Δ*E*_S-T_ of twisted **A-CH′**^2^^·^ is quite small. The transition state for this conversion has a quinoidal structure with a closed-shell configuration and a relative free energy of +31.5 kcal mol^–1^. Thus, the Δ*G*^‡^ value for transition of twisted **A-CH′**^2^^·^ to folded **A-CH′** is +19.9 kcal mol^–1^ (25 °C), which is in good agreement with the experimental result. Therefore, the results obtained both experimentally and computationally show that the generation of the triplet state from the folded form of **A-CH** using thermal activation would be difficult due to the presence of a high barrier. However, the alternative route we developed, using the kinetic trapping method starting with the dication species, serves as an effective method to generate and isolate the twisted triplet state of **A-CH**^2^^·^.

### Mechanical-Grinding-Induced Triplet State Generation of **A-CH**

As mentioned in the introduction above, in the AQD-embedded biradicaloids, thermal equilibrium between singlet and triplet states is linked to sterically governed, high activation barrier conformational changes between folded and twisted conformers. However, the only approaches developed prior to this study for generating triplet states are thermal activation and light irradiation. Recently, mechanical stress-induced C–C σ-bond cleavage, promoted by grinding, pulling, or pressing,^44−52^ has been used to produce two monoradical species. This protocol has gained attention for use in mechanical sensors and self-healing.

**Figure 9:**
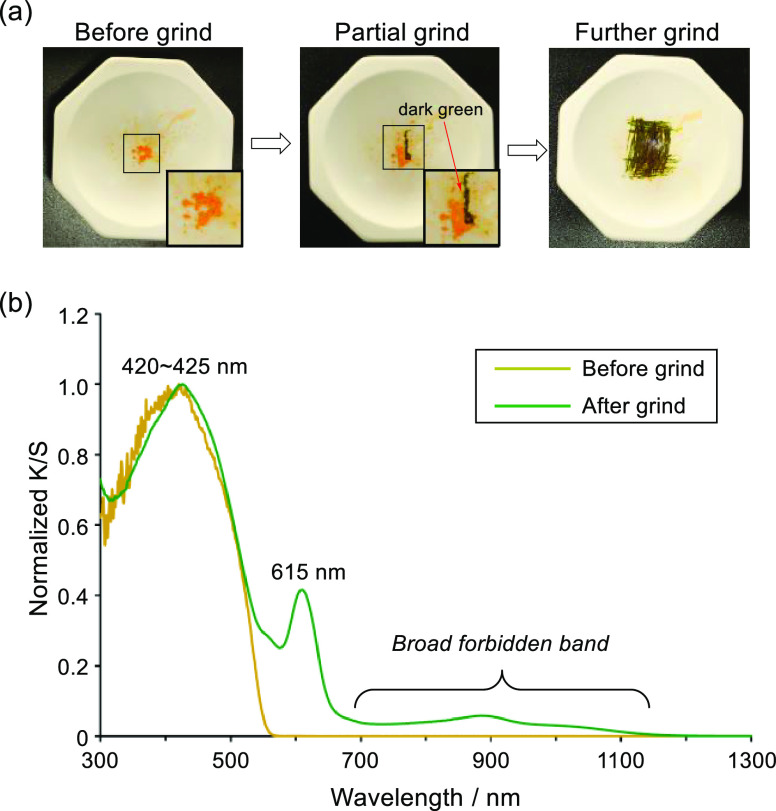
(a) Photoimages of before and after grinding of folded **A-CH**. The color at the ground part is changed from orange to dark green. (b) Solid-state diffuse-reflection UV–vis–NIR spectra before and after grinding of folded **A-CH**.

Owing to the presence of flexible FAE units in folded **A-CH**, it was anticipated that C=C π-bond cleavage to afford the twisted **A-CH**^2^^·^ in the solid state could be accomplished by grinding. In fact, grinding under air causes the orange color of solid folded **A-CH** to change to dark green (Figure 9a). Solid-state diffuse-reflection UV–vis–NIR absorption spectroscopy was used to analyze the substance formed by grinding after the color change is complete. Although the intense peak λ_abs_ = 420–425 nm corresponding to the folded **A-CH** remains in the spectrum, a new sharp peak at 615 nm is produced by grinding along with a weak broad band from 700 to 1100 nm that corresponds to triplet **A-CH**^2^^·^ (Figure 9b). In addition, the ESR spectrum of a cold (−78 °C) solution formed by grinding of folded **A-CH** contains signals associated with the triplet state of **A-CH**^2^^·^ (Figure S20). Judging from these observations, mechanical grinding of folded **A-CH** induces triplet generation by promoting C=C π-bond cleavage in the solid state. Although determination of the yield for conversion of folded **A-CH** to twisted **A-CH**^2^^·^ is difficult, the newly devised method enables us to prepare and analyze the triplet-state aromatic hydrocarbon under ambient conditions. In addition, being different from the solution state, the absorption of the twisted triplet **A-CH**^2^^·^ formed by grinding is persistent for a week at ambient temperatures in the solid state (Figure S21).

## Conclusions

In the investigation described above, we synthesized the π-extended versions of Thiele’s and Chichibabin’s hydrocarbons, **A-TH** and **A-CH**, which contain appended 9-anthryl moieties. The presence of flexible FAE components enables both **A-TH** and **A-CH** to undergo dynamic conformational changes, which can be quantitatively analyzed experimentally and computationally. The crystalline states of **A-TH** and **A-CH** possess quinoidal nonplanar structures. **A-TH** in the crystalline state has a unique unstable *anti*-folded shape, which is the intermediate in the folded-to-twisted conformational interconversion process. Although **A-CH** in its ground state exists in a folded conformation as a result of strong intramolecular spin–spin interactions, chemical oxidation experiments revealed that conformational change from folded neutral to twisted dication form can be induced. Reduction of twisted dication **A-CH**^2+^ with subsequent precipitation at low temperatures serves as a convenient method to produce the twisted triplet of **A-CH**^2^^·^. This finding enables elucidation of the fundamental properties of twisted **A-CH**^2^^·^. Furthermore, twisted triplet **A-CH**^2^^·^ can be generated by simple grinding of folded **A-CH** in the solid state. The unique dynamic behaviors of **A-TH** and **A-CH** in both solution and solid states originate from the effect of π-congestion caused by the presence of bulky 9-anthryl units. It is expected that π-congested multiradicaloids probed in future efforts will exhibit more complicated and interesting natures.

## Supporting Information Available

Experimental procedures, characterization of products, spectroscopic data, and theoretical calculations (PDF)

## Accession Codes

CCDC 2149926–2149927 contain the supplementary crystallographic data for this paper. These data can be obtained free of charge via www.ccdc.cam.ac.uk/data_request/cif, or by emailing data_request@ccdc.cam.ac.uk, or by contacting The Cambridge Crystallographic Data Centre, 12 Union Road, Cambridge CB2 1EZ, UK; fax: +44 1223 336033.
