# Supplementary material for: Synthesis of π-Extended Thiele’s and Chichibabin’s Hydrocarbons and Effect of the π-Congestion on Conformations and Electronic States
Source: J Am Chem Soc. 2022 Apr 15;144(16):7479–88. doi: 10.1021/jacs.2c02318 (PMC9136924; doi:10.1021/jacs.2c02318)
Supplement: Supplementary file 1 — ja2c02318_si_001.pdf [file ja2c02318_si_001.pdf]

Supporting Information

Synthesis of π-Extended Thiele's and Chichibabin's Hydrocarbons and Effect of the π-congestion on Conformations and Electronic States

Tomohiko Nishiuchi,*[a,b] Seito Aibara,[a] Hiroyasu Sato,[c] and Takashi Kubo*[a,b]

[a] Department of Chemistry, Graduate School of Science, Osaka University, 1-1 Machikaneyama, Toyonaka, Osaka 560-0043, Japan. E-mail: nishiuchit13@chem.sci.osaka-u.ac.jp

[b] Innovative Catalysis Science Division, Institute for Open and Transdisciplinary Research Initiatives, (ICS-OTRI), Osaka University, Suita, Osaka 565-0871, Japan.

[c] Rigaku Corporation, 3-9-12 Matsubara, Akishima, Tokyo 196-8666, Japan.

Contents

1. General information S2
2. Synthesis S2
3. VT-NMR of **A-TH** and **A-CH** at high temperature S4
4. 2D NMR of **A-TH** and **A-CH** at high temperature S6
5. VT-NMR of **A-TH** and **A-CH** at low temperature S8
6. Kohn-Sham molecular orbitals and TD-DFT calculations of **A-TH** and **A-CH** S10
7. Relative Energy Differences of **A-TH** Conformers S18
8. UV-vis NIR spectra of **TAntM** cation and **TAntM** radical S18
9. Spin density map of twisted triplet state of **A-CH'**$^{2•}$ S19
10. Decay plots of UV-vis absorption of **A-CH**$^{2•}$ at 5, 0 –5, –10, and –15 °C S20
11. ESR spectrum of folded **A-CH** after ground S20
12. Solid-state diffuse-reflection UV-vis-NIR spectra of **A-CH** after ground S21
13. X-ray crystallographic data S22
14. NMR spectra S24
15. Cartesian coordination of calculated structures S30

General information

All experiments with moisture- or air-sensitive compounds were performed in anhydrous solvents under nitrogen atmosphere in well-dried glassware. Dried solvents (THF and dichloromethane) were purchased from KANTO CHEMICAL. Column chromatography was performed with silica gel [Silica gel 60N (KANTO CHEMICAL)]. ^{1}H and ^{13}C NMR spectra were recorded on Bruker JEOL lambda-400 or Agilent VNS600 spectrometer. Data collection for X-ray crystal analysis was performed on Rigaku XtaLAB Synaergy Custom (Detector is Hypix-6000HE. Mo-Kα (λ = 0.71069 Å) or Cu- Kα (λ = 1.54187 Å)). The structure was solved with direct methods and refined with full-matrix least squares. The UV-vis spectra were recorded on JASCO V-570 spectrophotometer. The Solid state UV-vis spectra were recorded on JASCO V-570 spectrophotometer with ISN-470 integral sphere unit (Jasco). The obtained reflectance spectrum was converted to absorption spectra according to the Kubelka–Munk function.

Computational Methods. All DFT calculations were performed with the Gaussian 16 program. Structure optimizations were performed by (U)ωB97X-D/6-31G**. TD-DFT calculations were performed by (U)B3LYP/6-31G** method using the optimized structures by (U)ωB97X-D/6-31G** method.

Synthesis

Synthesis of compound A-TH

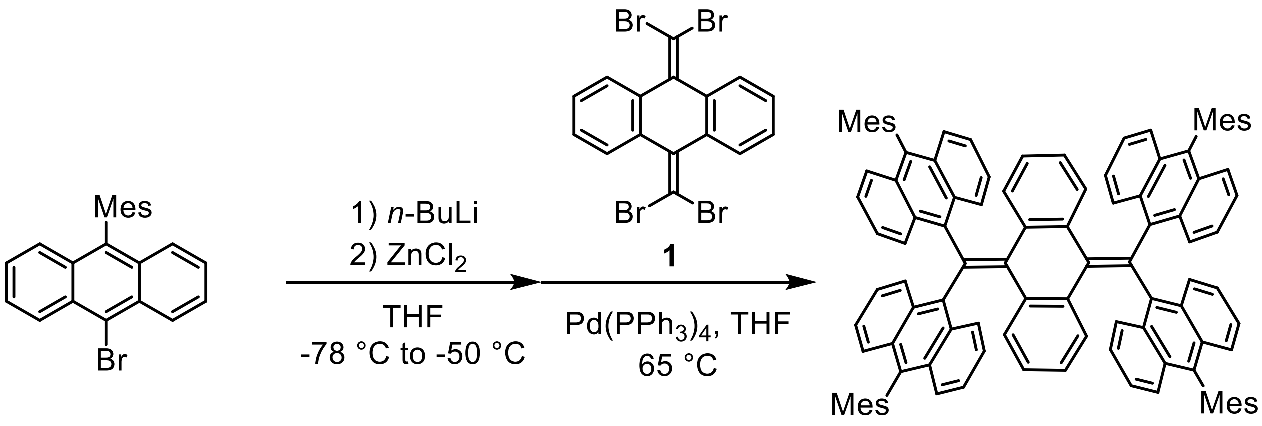

To a solution of 9-bromo-10-mesitylanthracene (2.30 g, 6.13 mmol) in THF (30 ml) was added *n*-BuLi (1.6 M hexane solution, 4.2 ml, 6.72 mmol) at -78 °C. After stirring for 1 h at same temperature, a suspension of zinc chloride (1.00 g, 7.34 mmol) in THF (15 ml) was added and stirred at -50 °C. After stirring for additional 1 h, a suspension of **1** (520 mg, 1.00 mmol) and $Pd(PPh_3)_4$ (360 mg, 0.312 mmol) in THF (15 ml) was added and heated up to 65 °C for 72 h. The reaction was quenched by water, and extracted with dichloromethane and washed with brine. After removal of the solvent *in vacuo*, the crude material was subjected to column chromatography on silica gel (toluene : hexane = 1 : 1 to 3 : 2) to afford the title compound **A-TH** (360 mg, 0.261 mmol, 26%) as reddish-brown solid. Mp: 283-284 °C (dec.). ^{1}H NMR (600 MHz, $C_2D_2Cl_4$, 80 °C) δ 8.93 (d, *J* = 8.4 Hz, 8H, aromatic proton), 7.89-7.87 (m, 4H, aromatic proton), 7.22 (d, *J* =9.0 Hz, 8H, aromatic proton), 7.17 (br, 8H, aromatic proton), 7.02 (t, *J* =7.2 Hz, 8H, aromatic proton), 6.94 (s, 4H, aromatic proton), 6.86 (s, 4H, aromatic proton), 6.32-6.31 (m, 4H, aromatic proton), 2.31 (s, 12H, C*H*$_3$), 1.67 (s, 12H, C*H*$_3$), 1.08 (s, 12H, C*H*$_3$); ^{13}C NMR (150 MHz, $C_2D_2Cl_4$, 80 °C) δ 141.06, 139.85, 137.36, 136.94, 136.80, 136.66, 136.31, 135.83, 134.75, 131.05, 129.62, 128.47, 128.02, 127.81, 127.64, 126.41, 126.14, 125.07, 124.50, 21.03, 19.64, 19.00. HR-MS (APCI) Calcd for $C_{108}H_{84}$ [$(M$+H$)^+$]: *m/z* 1381.6601, Found: 1381.6626.

Synthesis of compound A-CH

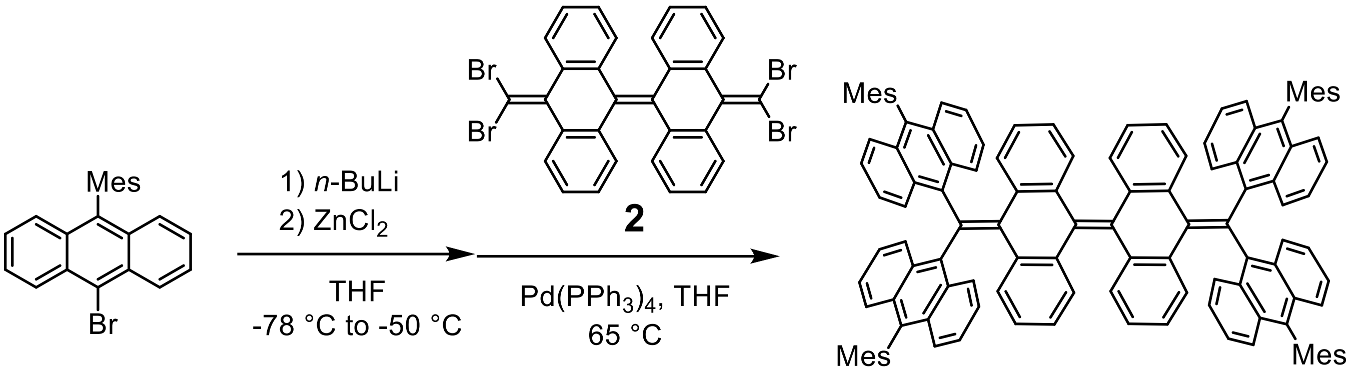

A-CH

To a solution of 9-bromo-10-mesitylanthracene (2.80 g, 7.46 mmol) in THF (40 ml) was added *n*-BuLi (1.6 M hexane solution, 5.0 ml, 8.00 mmol) at -78 °C. After stirring for 1 h at same temperature, a suspension of zinc chloride (1.20 g, 8.80 mmol) in THF (15 ml) was added and stirred at -50 °C. After stirring for additional 1 h, a suspension of **2** (1.00 g, 1.44 mmol) and $Pd(PPh_3)_4$ (175 mg, 0.152 mmol) in THF (15 ml) was added and heated up to 65 °C for 12 h. The reaction was quenched by water, and extracted with dichloromethane and washed with brine. After removal of the solvent *in vacuo*, the crude material was subjected to column chromatography on silica gel (toluene : hexane = 1 : 1 to 3 : 2) to afford the title compound **A-CH** (1.60 g, 1.03 mmol, 72%) as reddish-brown solid. Mp: 370 °C (dec.), ^{1}H NMR (400 MHz, $C_2D_2Cl_4$, 80 °C) δ 8.83 (d, J = 8.8 Hz, 4H, aromatic proton), 8.79 (d, J = 9.2 Hz, 4H, aromatic proton), 7.62 (d, J = 6.8 Hz, 4H, aromatic proton), 7.36 (d, J = 8.8 Hz, 4H, aromatic proton), 7.30 (d, J = 8.8 Hz, 4H, aromatic proton), 7.20 (d, J = 8.4 Hz, 4H, aromatic proton), 7.09-6.80 (m, 28H, aromatic proton), 6.51 (t, J = 7.6 Hz, 4H, aromatic proton), 2.35 (s, 12H, C*H₃*), 1.66 (s, 12H, C*H₃*), 1.48 (s, 12H, C*H₃*); ^{13}C NMR (100 MHz, $C_2D_2Cl_4$, 80 °C) δ 138.17, 138.04, 137.37, 137.20, 137.06, 136.91, 136.82, 134.80, 132.21, 129.81, 129.48, 128.08, 128.05, 127.99, 127.60, 127.43, 126.86, 126.51, 126.32, 126.03, 125.35, 124.83, 124.61, 21.07, 19.63, 19.51. HR-MS (APCI) Calcd for $C_{122}H_{92}$ [$(M$+H$)^+$]: *m*/*z* 1557.7227, Found: 1557.7218.

Synthesis of compound A-CH$^{2\bullet}$

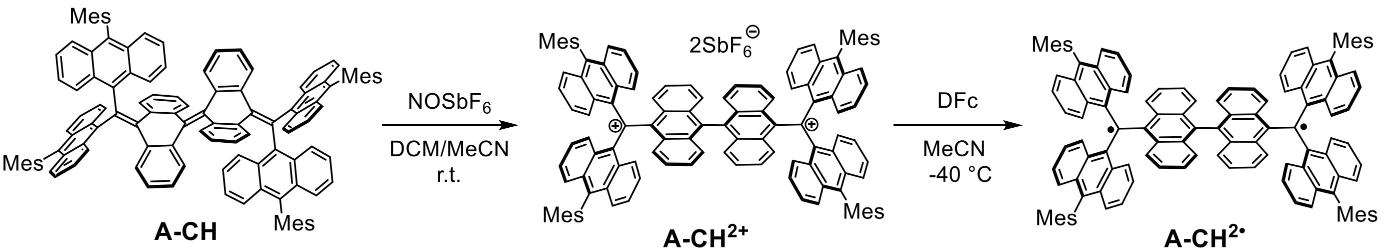

To a solution of **A-CH** (160 mg, 0.103 mmol) in CH_2Cl_2 (5 ml) was added $NOSbF_6$ (56 mg, 0.211 mmol in 2 ml CH_3CN solution) at room temperature. After stirring for 1 h, the solution was evaporated and dissolved again in a small amount of CH_3CN. The insoluble residue was removed by filtration and the filtrate was evaporated to afford the **A-CH^{2+}** as dark red solid. The obtained dark red solid was used for next reaction without further purification. The solid of **A-CH^{2+}** was dissolved in 5 ml CH_3CN and the solution was cooled at −40 °C. A solution of decamethylferrocene (DFc, 72 mg, 0.221 mmol) in 5 ml CH_3CN was slowly added to the solution of **A-CH^{2+}** at the same temperature. After keeping the temperature for 1h, the precipitation was collected by filtration to afford the title compound **A-CH$^{2\bullet}$** (97 mg, 0.0623 mmol, 61% (2 steps)) as metallic purple solid. ^{1}H NMR of **A-CH^{2+}** (400 MHz, CD_2Cl_2) δ 8.40 (d, J = 8.8 Hz, 4H, aromatic proton), 8.30 (d, J = 9.2 Hz, 4H, aromatic proton), 8.12 (d, J = 9.2 Hz, 4H, aromatic proton), 7.72-7.65 (m(br), 8H, aromatic proton), 7.50-7.40 (m(br), 8H, aromatic proton), 7.40-7.33 (m(br), 8H, aromatic proton), 7.30-7.20 (m, 20H, aromatic proton), 2.49 (s, 12H, C*H₃*), 1.94 (s, 12H, C*H₃*), 1.90 (s, 12H, C*H₃*); ^{13}C NMR of **A-CH^{2+}** (100 MHz, CD_2Cl_2) δ 206.76, 147.55, 139.92, 137.29, 135.86, 134.49, 132.87, 131.91, 130.20, 129.26, 129.21, 128.86, 126.11, 30.97, 21.40, 20.23. (10 aromatic carbons out of 23 are missing probably due to duplication of the peaks and the peak coalescing originating from the rotational flexibility of anthryl rings.)

VT-NMR of A-TH and A-CH at high temperature

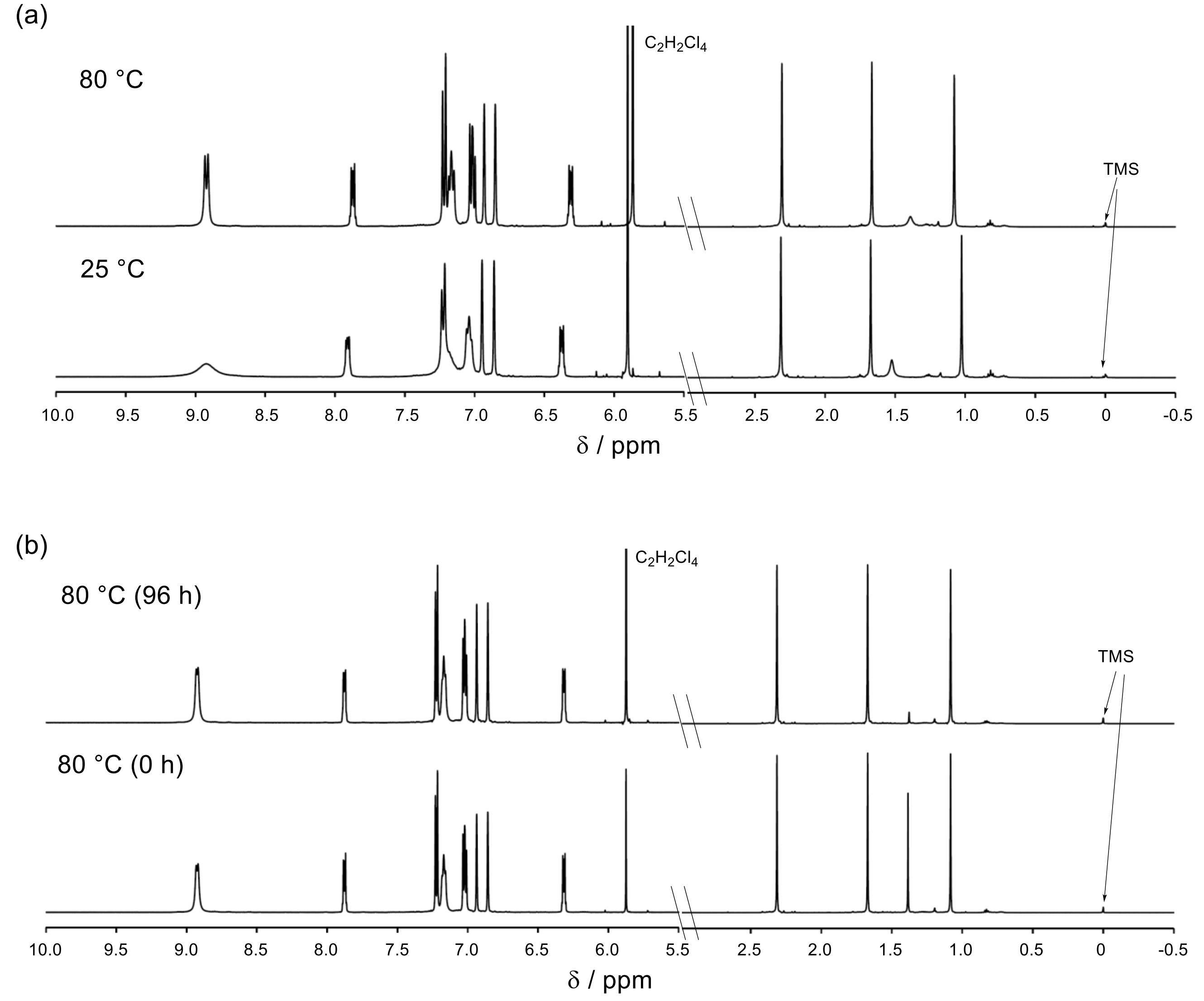

Figure S1. (a) ^{1}H-NMR spectra of **A-TH** at 25 °C and 80 °C. (b) Evaluation of the thermal persistence of **A-TH** at 80 °C. No spectrum changing was observed after 96 h left at 80 °C. These ^{1}H-NMR spectra were measured in 1,1,2,2-tetrachloroethane-d_2.

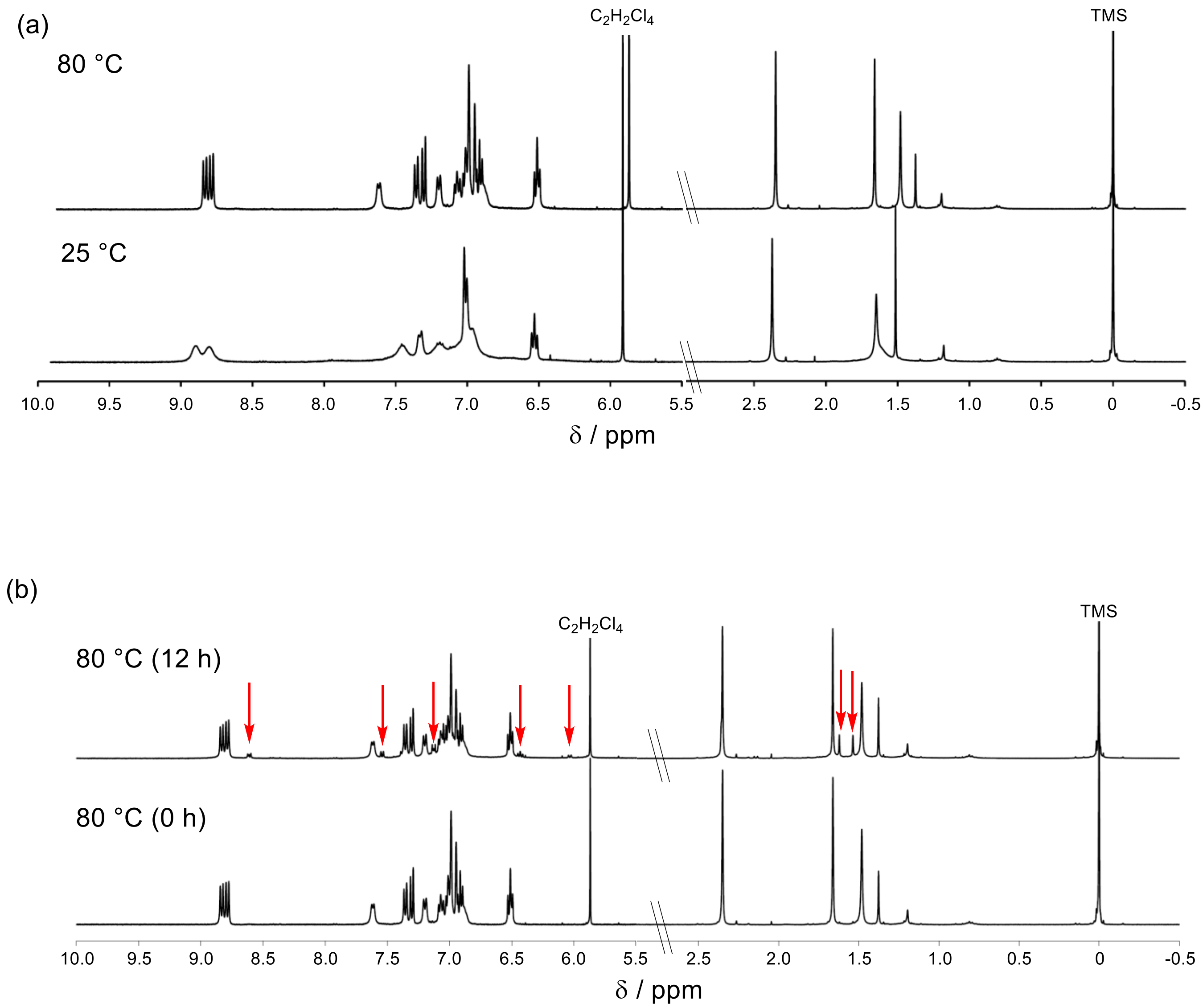

Figure S2. (a) ^{1}H NMR spectra of **A-CH** at 25 °C and 80 °C. (b) Evaluation of the thermal persistence of **A-CH** at 80 °C. Several new peaks were observed after 12 h left at 80 °C, indicating that there is structural isomerization or decomposition. These ^{1}H-NMR spectra were measured in 1,1,2,2-tetrachloroethane-d_2.

2D NMR of A-TH and A-CH at high temperature

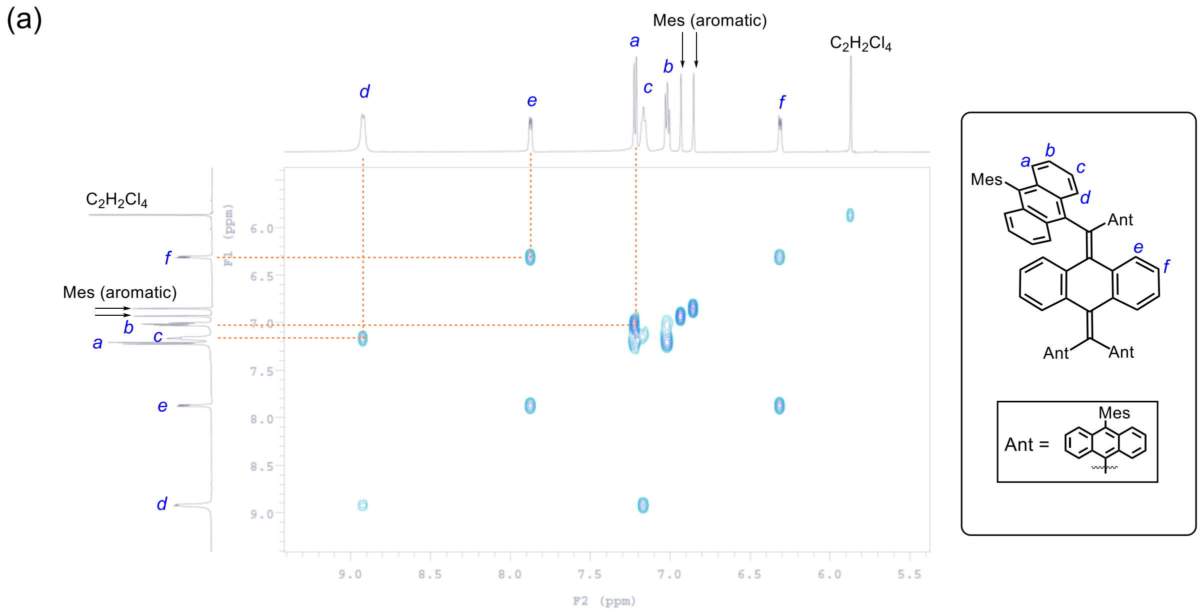

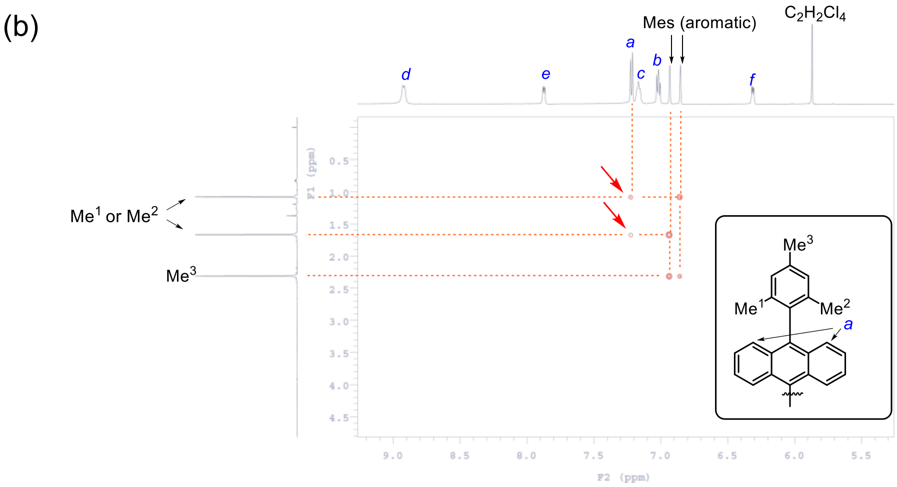

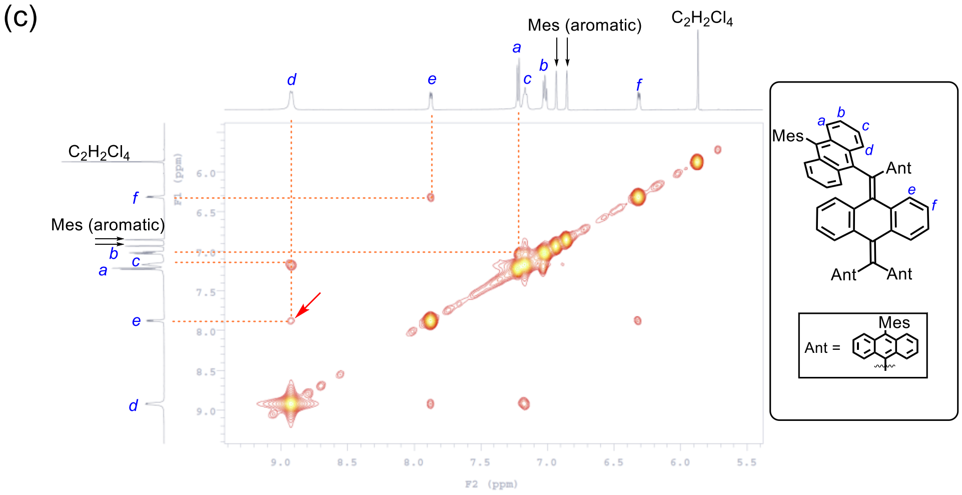

Figure S3. (a) ^{1}H-^{1}H COSY of **A-TH** in low magnetic field. (b) NOESY spectrum of **A-TH** in high and low magnetic fields to evaluate the coupling between methyl protons (Me^1 and Me^2) and anthryl protons *a*, which is indicated by red arrows. (c) NOESY spectrum of **A-TH** in low magnetic field to evaluate the coupling between *d* and *e,* which is indicated by red arrows. (Solvent: 1,1,2,2-tetrachloroethane-d_2 at 80 °C.)

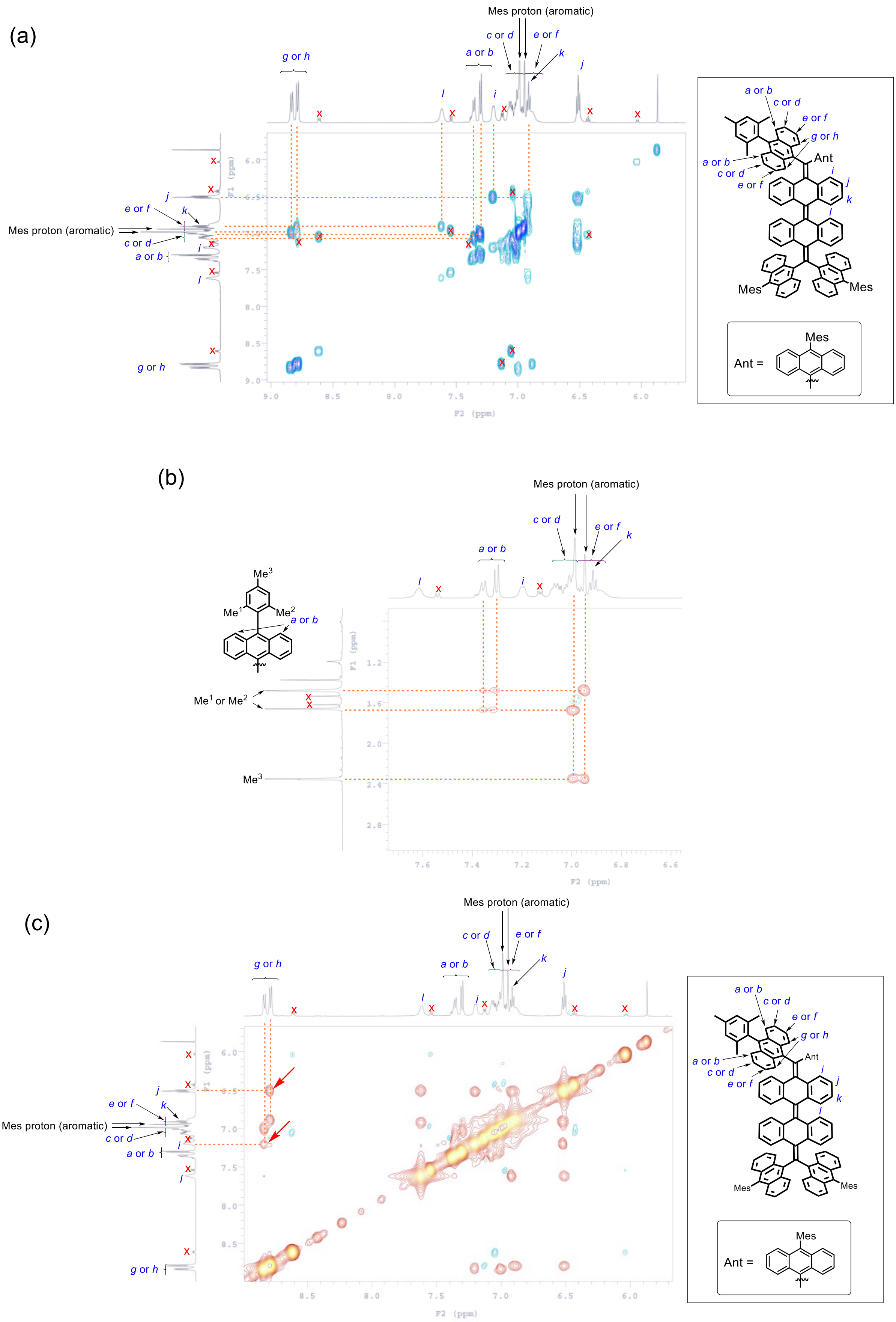

Figure S4. (a) ^{1}H-^{1}H COSY of **A-CH** in low magnetic field. (b) NOESY spectrum of **A-CH** in high and low magnetic fields to evaluate the coupling between methyl protons (Me^1 and Me^2) and anthryl protons *a* or *b*. (c) NOESY spectrum of **A-CH** in low magnetic field to evaluate the coupling between *g* or *h* and *i* or *j*, which is indicated by red arrows. Red x indicates conformational isomers or decomposed species due to the high temperature. (Solvent: 1,1,2,2-tetrachloroethane-d_2 at 80 °C.)

VT-NMR of A-TH and A-CH at low temperature

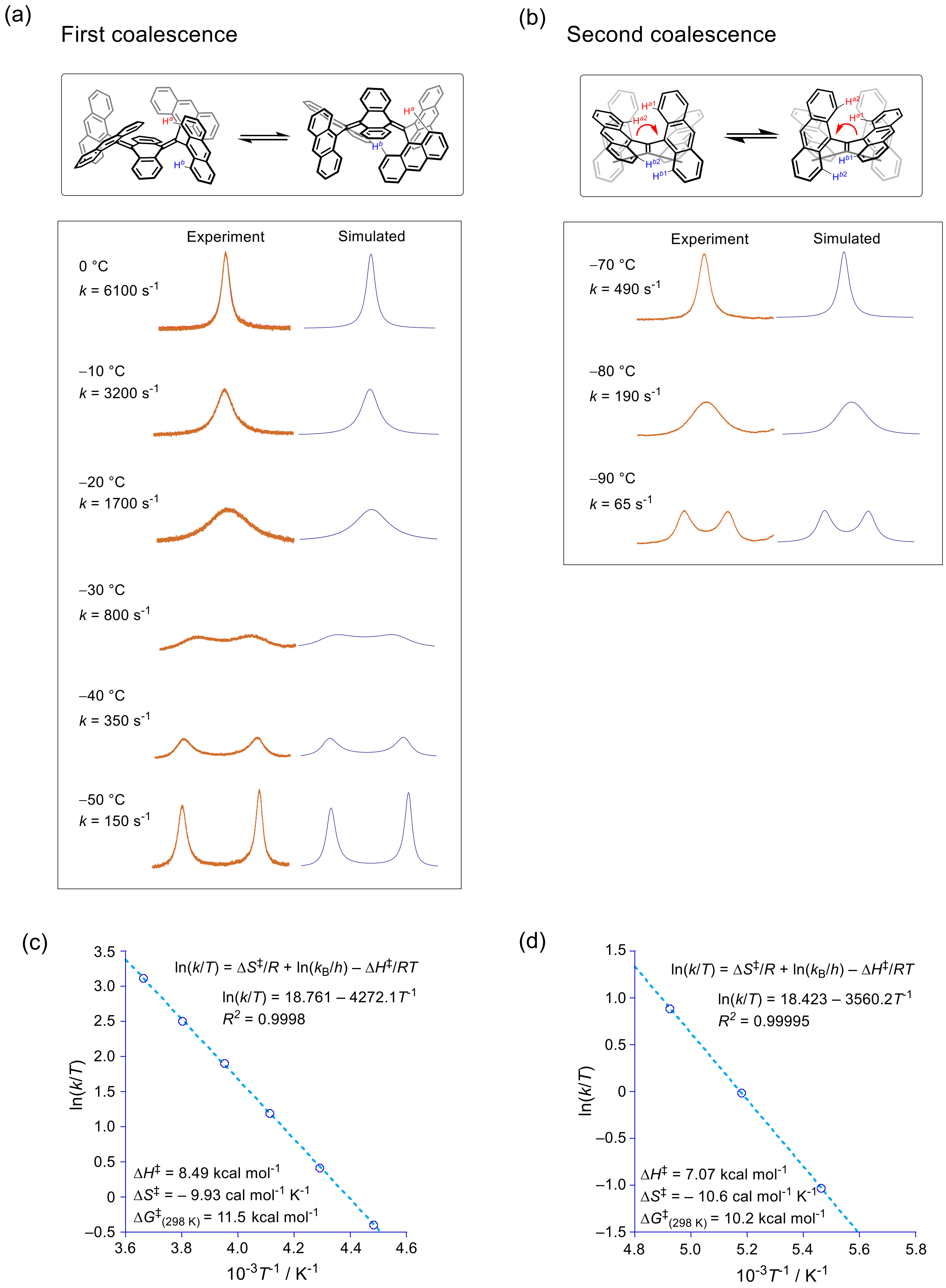

Figure S5. (a) Curve-fitting analysis of the first coalescence of **A-TH** at *d* proton (*ca*. 9.1 ppm) from 0 °C to –50 °C. (b) Curve-fitting analysis of the second coalescence of **A-TH** at *d* proton (8.0~8.75 ppm) from -70 °C to –90 °C. (c) Eyring plot for the first coalescence and its thermodynamic parameters. (d) Eyring plot for the second coalescence and its thermodynamic parameters.

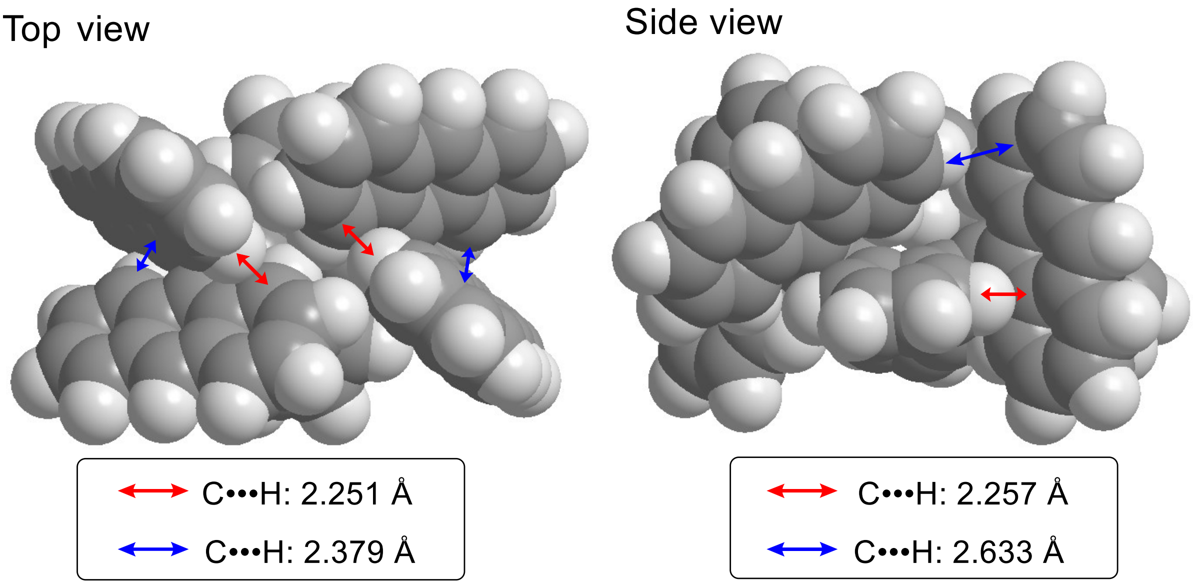

Figure S6. CH-π interaction between adjacent anthryl units of *syn*-folded **A-TH'** (ωB97X-D/6-31G**).

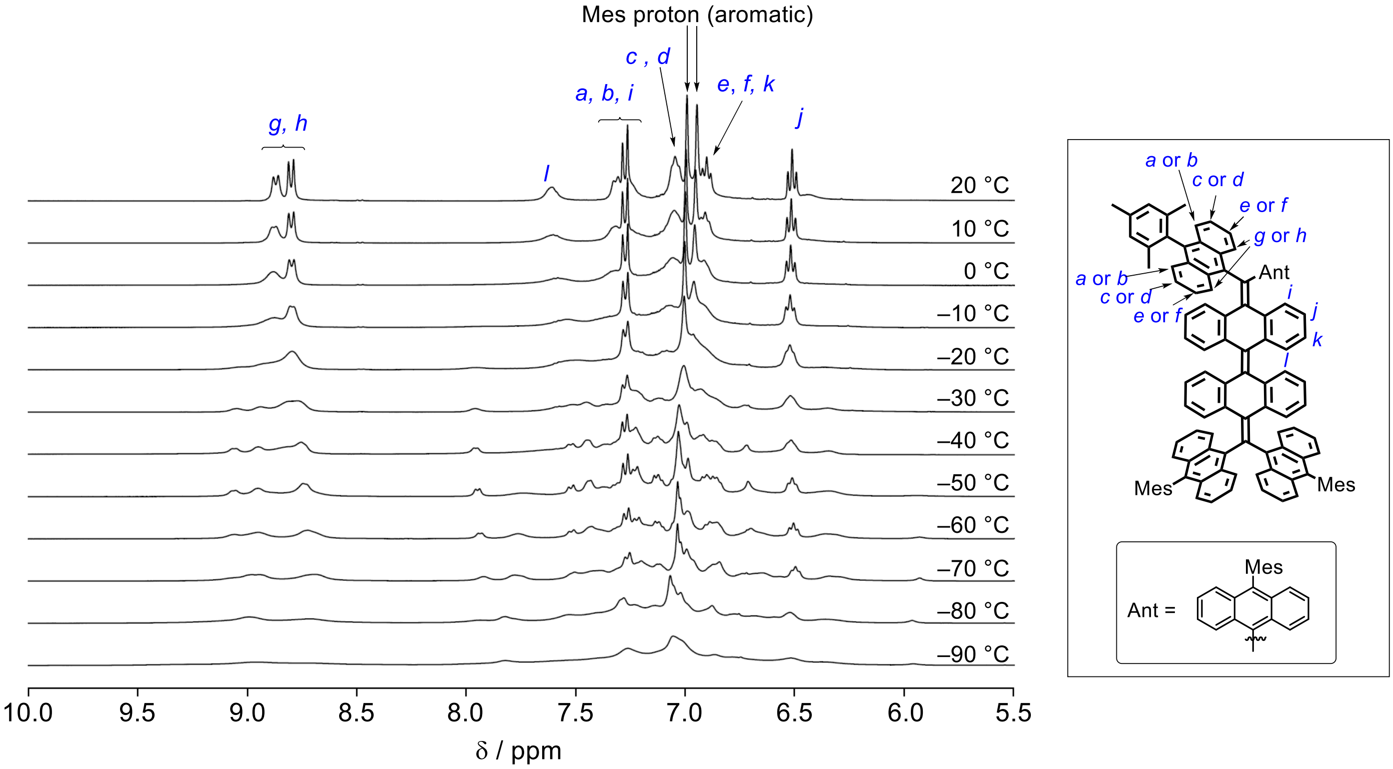

Figure S7. VT NMR spectra of **A-CH** in low magnetic field (5.5~10.0 ppm) from 20 °C to –90 °C in CD_2Cl_2.

Kohn-Sham molecular orbitals and TD-DFT calculations of A-TH and A-CH

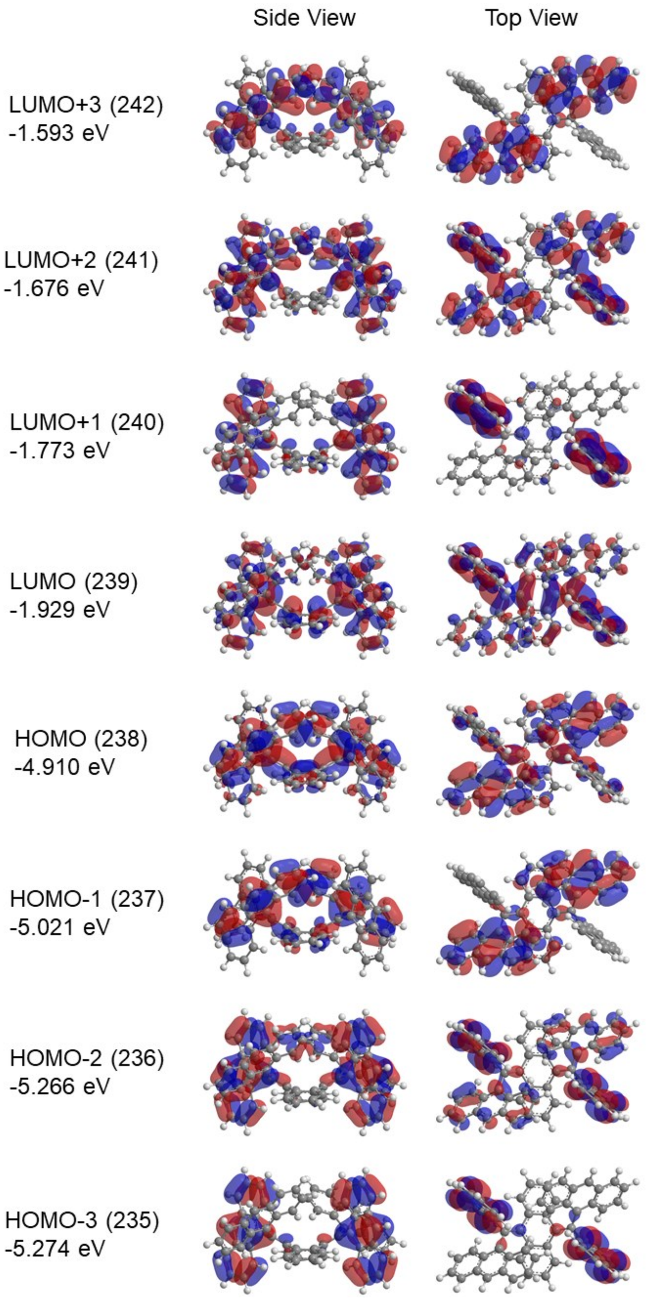

Excited State 1: Singlet-A 2.4940 eV 497.13 nm f=0.1905
237 -> 240 -0.16431
238 -> 239 0.68155

Excited State 2: Singlet-A 2.5782 eV 480.90 nm f=0.0064
237 -> 239 0.58344
237 -> 241 0.11417
238 -> 240 -0.36202

Excited State 3: Singlet-A 2.6999 eV 459.21 nm f=0.0025
237 -> 239 0.37635
237 -> 241 -0.10378
238 -> 240 0.57942

Excited State 4: Singlet-A 2.7290 eV 454.32 nm f=0.0718
236 -> 239 -0.24876
237 -> 240 -0.42583
238 -> 239 -0.13652
238 -> 241 0.47816

Excited State 5: Singlet-A 2.8424 eV 436.20 nm f=0.0061
236 -> 239 -0.14860
237 -> 240 0.53176
238 -> 239 0.10362
238 -> 241 0.41366

Excited State 6: Singlet-A 2.8781 eV 430.79 nm f=0.0096
235 -> 239 0.56961
235 -> 241 -0.19680
236 -> 242 0.15376
237 -> 241 -0.11603
238 -> 242 0.30169

Excited State 7: Singlet-A 2.8798 eV 430.53 nm f=0.1210
236 -> 239 0.61317
238 -> 241 0.29750

Excited State 8: Singlet-A 2.9051 eV 426.78 nm f=0.0052
235 -> 239 -0.33988
238 -> 242 0.59423

Excited State 9: Singlet-A 2.9557 eV 419.47 nm f=0.0213
237 -> 241 0.66678
238 -> 240 0.14650
238 -> 242 0.11805

Excited State 10: Singlet-A 3.0578 eV 405.47 nm f=0.1034
237 -> 242 0.68712

Figure S8. Kohn-Sham molecular orbitals and its energies of *syn*-folded **A-TH'** (without Mes substitution of **A-TH**, left), and TD-DFT calculated results (right). (B3LYP/6-31G**//ωB97X-D/6-31G**)

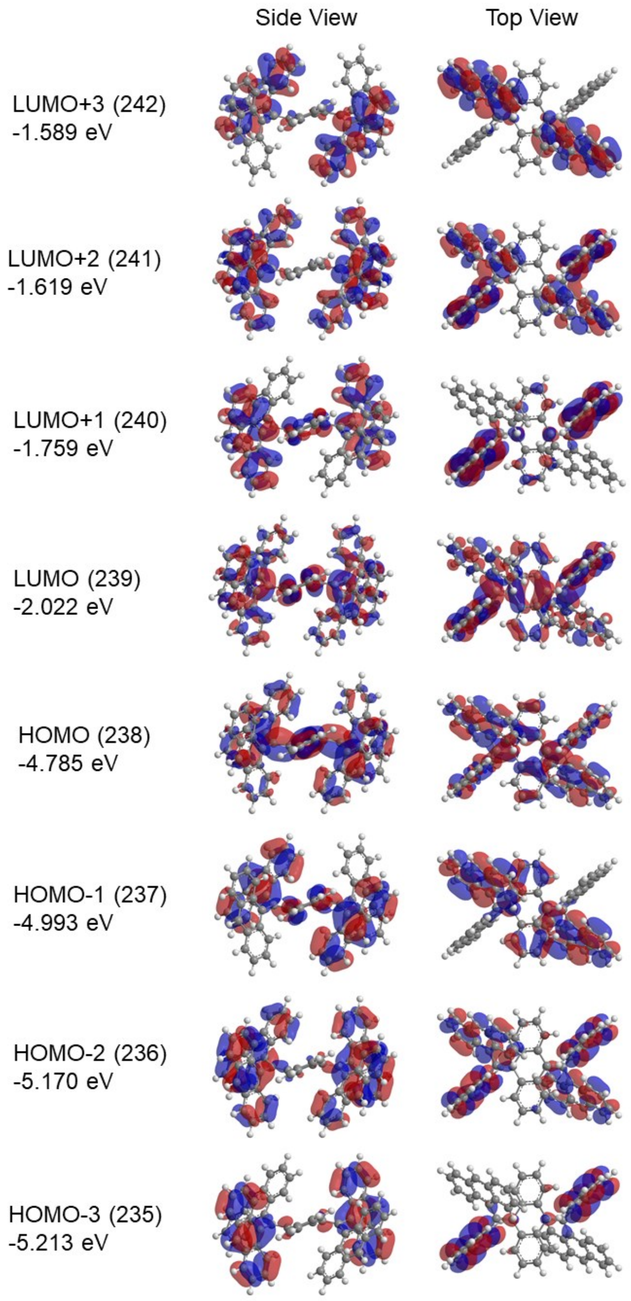


```
Excited State   1:      Singlet-AU     2.3180 eV  534.88 nm  f=0.3736
   237 -> 240       -0.10411
   238 -> 239        0.69596

Excited State   2:      Singlet-AG     2.4801 eV  499.92 nm  f=0.0000
   237 -> 239        0.61089
   237 -> 241       -0.10175
   238 -> 240       -0.30019

Excited State   3:      Singlet-AG     2.5637 eV  483.61 nm  f=0.0000
   237 -> 239        0.31849
   238 -> 240        0.61460

Excited State   4:      Singlet-AU     2.6159 eV  473.96 nm  f=0.0416
   236 -> 239        0.48158
   237 -> 240       -0.25796
   238 -> 241       -0.42715

Excited State   5:      Singlet-AG     2.7308 eV  454.02 nm  f=0.0000
   235 -> 239        0.43441
   235 -> 241        0.12532
   236 -> 242       -0.14038
   238 -> 242        0.50775

Excited State   6:      Singlet-AU     2.7384 eV  452.75 nm  f=0.1540
   236 -> 239        0.49003
   237 -> 240        0.14234
   238 -> 241        0.47137

Excited State   7:      Singlet-AG     2.7544 eV  450.13 nm  f=0.0000
   235 -> 239        0.52313
   238 -> 242       -0.45456

Excited State   8:      Singlet-AU     2.7994 eV  442.89 nm  f=0.0093
   237 -> 240        0.62717
   238 -> 241       -0.28133

Excited State   9:      Singlet-AG     2.9829 eV  415.65 nm  f=0.0000
   236 -> 240        0.12613
   237 -> 241        0.67136
   238 -> 240       -0.12233

Excited State  10:      Singlet-AU     3.0178 eV  410.85 nm  f=0.0884
   234 -> 239       -0.36987
   235 -> 240        0.10141
   237 -> 242        0.47903
   238 -> 243        0.32978
```

Figure S9. Kohn-Sham molecular orbitals and its energies of *anti*-folded **A-TH'** (without Mes substitution of **A-TH**, left), and TD-DFT calculated results (right). (B3LYP/6-31G**//ωB97X-D/6-31G**)

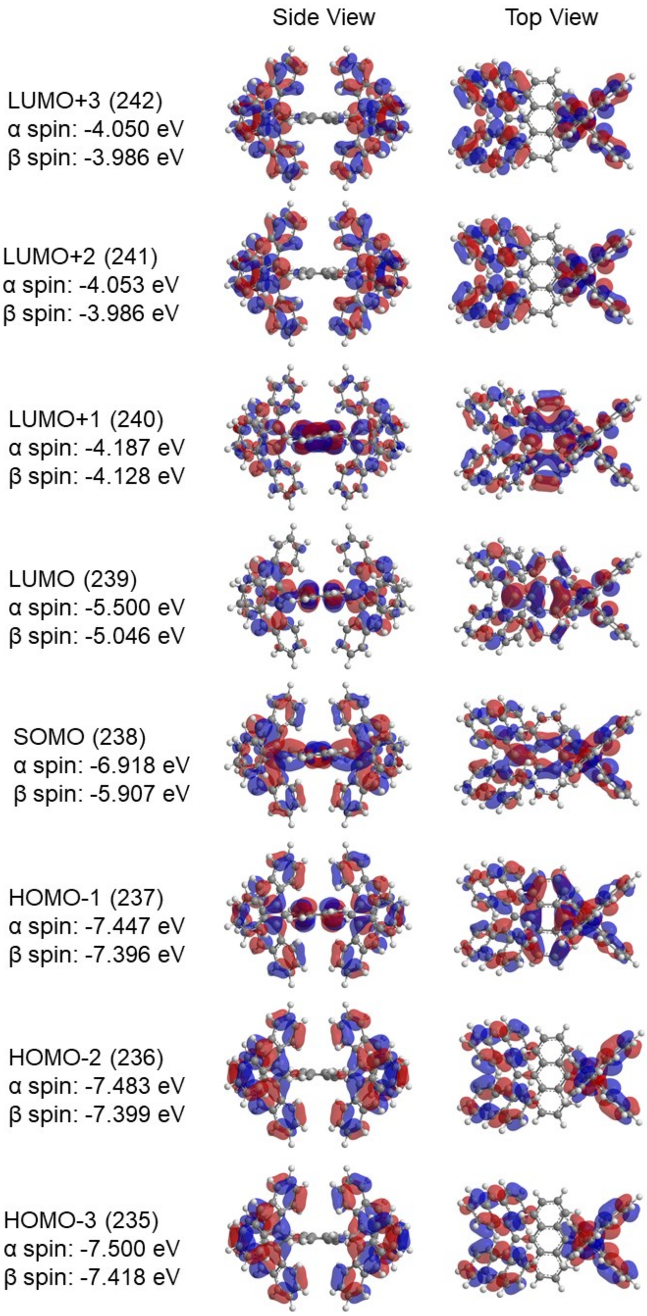


```
Excited State   1:  2.025-B1    0.7313 eV 1695.36 nm  f=0.0158  <S**2>=0.775
   238A -> 239A       0.73613
   233B -> 238B       0.10213
   236B -> 238B       0.66172

Excited State   2:  2.267-B2    0.9305 eV 1332.39 nm  f=0.0158  <S**2>=1.035
   235A -> 239A      -0.28506
   235B -> 239B       0.18259
   237B -> 238B       0.92930

Excited State   3:  2.291-B3    0.9434 eV 1314.22 nm  f=0.0212  <S**2>=1.063
   236A -> 239A      -0.29790
   235B -> 238B       0.92171
   237B -> 239B       0.19813

Excited State   4:  2.076-B1    1.0450 eV 1186.47 nm  f=0.4193  <S**2>=0.828
   237A -> 240A      -0.12062
   238A -> 239A      -0.63270
   234B -> 239B       0.15646
   236B -> 238B       0.73647

Excited State   5:  3.207-A     1.2494 eV  992.36 nm  f=0.0000  <S**2>=2.321
   237A -> 239A       0.87495
   238A -> 240A       0.11885
   234B -> 238B      -0.24295
   236B -> 239B      -0.35354

Excited State   6:  3.080-B3    1.4207 eV  872.67 nm  f=0.0320  <S**2>=2.121
   235A -> 240A      -0.10645
   236A -> 239A       0.86424
   235B -> 238B       0.36849
   235B -> 240B       0.11247
   237B -> 239B      -0.26243

Excited State   7:  3.108-B2    1.4413 eV  860.21 nm  f=0.0218  <S**2>=2.165
   235A -> 239A       0.86251
   236A -> 240A      -0.11222
   235B -> 239B      -0.27886
   237B -> 238B       0.35278
   237B -> 240B       0.12161

Excited State   8:  2.314-A     1.6034 eV  773.24 nm  f=0.0000  <S**2>=1.088
   233A -> 239A       0.20921
   237A -> 239A       0.19819
   238A -> 244A      -0.12283
   233B -> 239B      -0.18800
   234B -> 238B       0.90496
   236B -> 239B      -0.15407

Excited State   9:  2.712-B1    1.7114 eV  724.46 nm  f=0.0022  <S**2>=1.588
   234A -> 239A       0.39008
   235A -> 242A      -0.15839
   236A -> 241A      -0.16023
   237A -> 240A      -0.18573
   238A -> 239A      -0.10175
   238A -> 243A       0.21858
   233B -> 238B       0.72942
   234B -> 239B      -0.30592
   235B -> 242B       0.14952
   236B -> 240B       0.13401
   237B -> 241B       0.14961

Excited State  10:  2.368-A     1.7194 eV  721.07 nm  f=0.0000  <S**2>=1.152
   235A -> 241A       0.16297
   236A -> 242A       0.16628
   237A -> 239A       0.42270
   237A -> 243A       0.11114
   238A -> 240A      -0.36124
   235B -> 241B      -0.13654
   236B -> 239B       0.75330
   237B -> 242B      -0.13760
```

Figure S10. Kohn-Sham molecular orbitals and its energies of radical cation state of **A-TH'** (**A-TH'**$^{\bullet +}$, left), and TD-DFT calculated results (right). (UB3LYP/6-31G**//UωB97X-D/6-31G**)

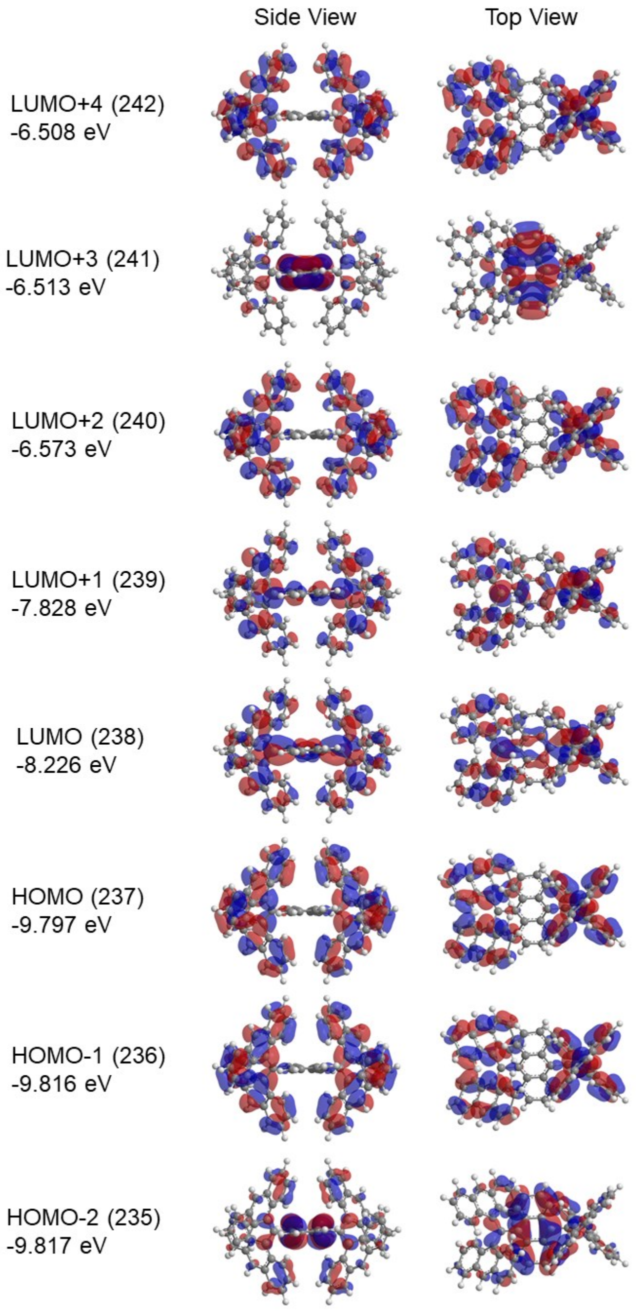


```
Excited State   1:      Singlet-B1     1.1072 eV 1119.80  nm  f=0.1514
    235 -> 238        0.70534

Excited State   2:      Singlet-B3     1.2659 eV  979.39 nm  f=0.0573
    237 -> 238        0.70617

Excited State   3:      Singlet-B2     1.3175 eV  941.04 nm  f=0.0648
    236 -> 238        0.69357
    237 -> 239       -0.15061

Excited State   4:      Singlet-A     1.5035 eV  824.63 nm  f=0.0000
    235 -> 239        0.70210

Excited State   5:      Singlet-B3     1.6493 eV  751.73 nm  f=0.0998
    236 -> 239        0.70402

Excited State   6:      Singlet-B2     1.6599 eV  746.95 nm  f=0.1507
    236 -> 238        0.15877
    237 -> 239        0.69249

Excited State   7:      Singlet-A     2.1089 eV  587.90 nm  f=0.0000
    234 -> 238        0.69651

Excited State   8:      Singlet-B1     2.1345 eV  580.87 nm  f=0.0307
    233 -> 238        0.69626

Excited State   9:      Singlet-B3     2.4043 eV  515.67 nm  f=0.0004
    229 -> 238        0.29302
    230 -> 239       -0.10363
    232 -> 238        0.63174

Excited State  10:      Singlet-B1     2.4460 eV  506.89 nm  f=0.0366
    227 -> 238       -0.17739
    228 -> 239       -0.13506
    231 -> 238        0.59138
    234 -> 239        0.30249
```

Figure S11. Kohn-Sham molecular orbitals and its energies of dication state of **A-TH’** (**A-TH’**$^{2+}$, left), and TD-DFT calculated results (right). (B3LYP/6-31G**//ωB97X-D/6-31G**)

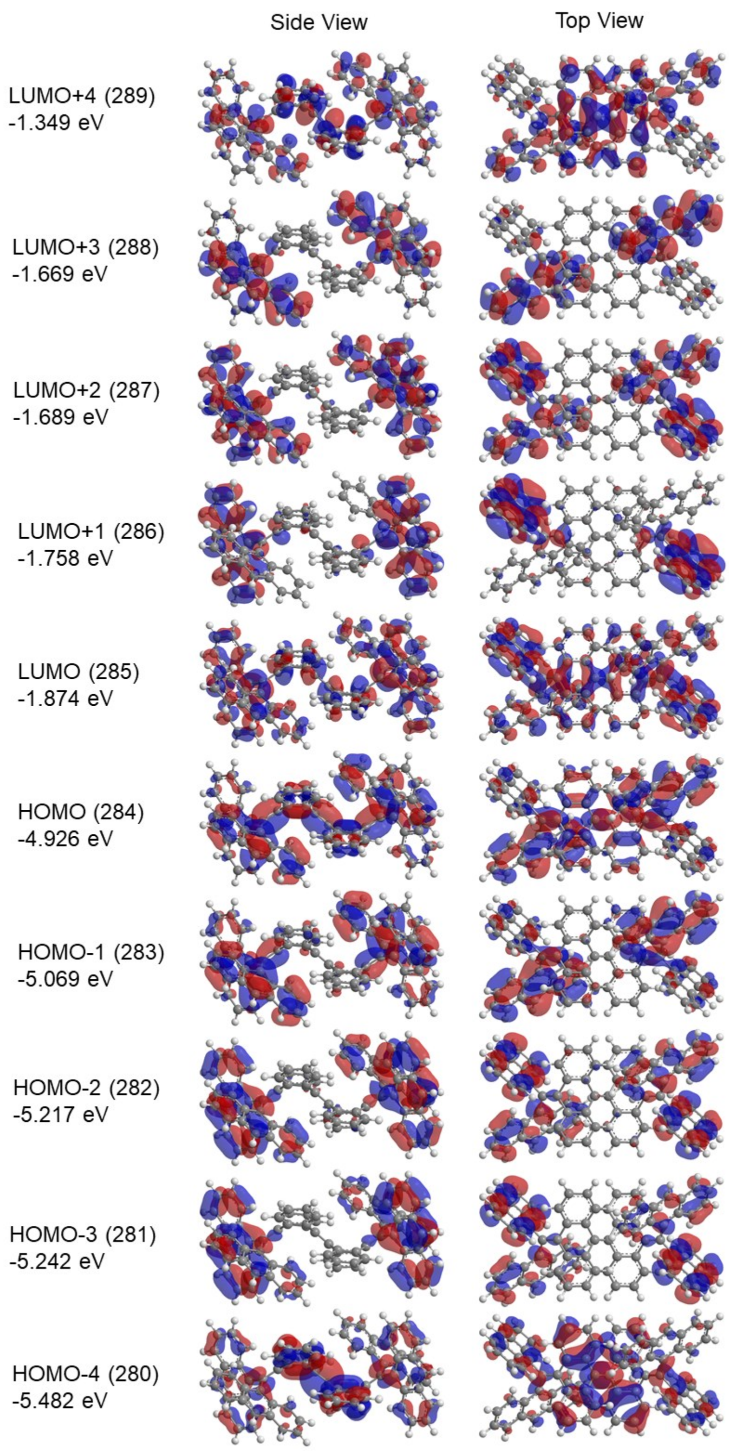

Excited State 1: Singlet-A 2.5796 eV 480.63 nm f=0.3486
283 -> 286 0.18404
284 -> 285 0.67456

Excited State 2: Singlet-A 2.6859 eV 461.62 nm f=0.0000
281 -> 285 -0.10358
282 -> 286 -0.11744
283 -> 285 0.40155
283 -> 287 0.15794
284 -> 286 0.52554

Excited State 3: Singlet-A 2.7549 eV 450.04 nm f=0.0417
281 -> 286 -0.12554
282 -> 285 -0.24507
283 -> 286 0.24827
283 -> 288 0.18334
284 -> 287 0.55914

Excited State 4: Singlet-A 2.8015 eV 442.57 nm f=0.0000
281 -> 285 -0.15873
281 -> 287 0.10171
282 -> 288 0.13129
283 -> 285 -0.34366
283 -> 287 0.22661
284 -> 288 0.50846

Excited State 5: Singlet-A 2.8344 eV 437.42 nm f=0.0000
281 -> 285 -0.13956
283 -> 285 0.42802
283 -> 289 0.12483
284 -> 286 -0.40833
284 -> 288 0.30116

Excited State 6: Singlet-A 2.9035 eV 427.02 nm f=0.1467
281 -> 286 0.18938
281 -> 288 -0.13198
282 -> 285 0.57336
284 -> 287 0.29514
284 -> 289 -0.10033

Excited State 7: Singlet-A 2.9262 eV 423.70 nm f=0.0000
281 -> 285 0.55092
282 -> 286 0.23191
282 -> 288 -0.13519
284 -> 288 0.30196

Excited State 8: Singlet-A 2.9481 eV 420.56 nm f=0.0121
280 -> 285 -0.10226
281 -> 288 -0.16045
282 -> 287 -0.20164
283 -> 286 0.55941
283 -> 288 -0.12851
284 -> 285 -0.16112
284 -> 287 -0.21277
284 -> 289 -0.10020

Excited State 9: Singlet-A 3.0368 eV 408.28 nm f=0.0000
281 -> 285 0.13424
282 -> 288 -0.10127
283 -> 287 0.61511
284 -> 286 -0.17811
284 -> 288 -0.14834

Excited State 10: Singlet-A 3.0576 eV 405.50 nm f=0.1222
281 -> 288 0.18265
282 -> 285 0.15804
282 -> 287 0.17184
283 -> 286 0.19133
283 -> 288 0.54492
284 -> 287 -0.19389
284 -> 289 0.13582

Figure S12. Kohn-Sham molecular orbitals and its energies of folded **A-CH**’ (without Mes substitution of **A-CH**, left), and TD-DFT calculated results (right). (B3LYP/6-31G**//ωB97X-D/6-31G**)

LUMO+5 (289)
-5.981 eV

LUMO+4 (288)
-5.992 eV

LUMO+3 (287)
-6.058 eV

LUMO+2 (286)
-6.063 eV

LUMO+1 (285)
-7.547 eV

LUMO (284)
-7.575 eV

HOMO (283)
-9.330 eV

HOMO-1 (282)
-9.332 eV

HOMO-2 (281)
-9.371 eV

HOMO-3 (280)
-9.428 eV

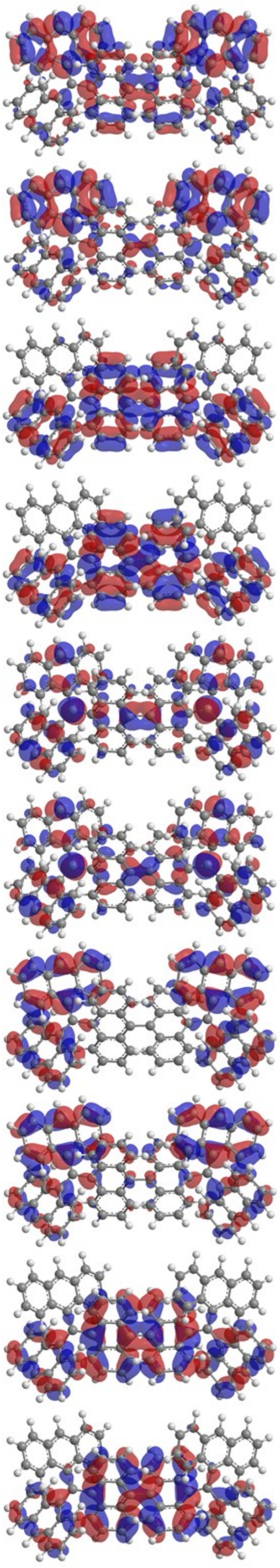

Excited State 1: Singlet-B 1.3401 eV 925.21 nm f=0.2928
280 -> 285 0.29979
281 -> 284 0.50974
282 -> 284 -0.35449
283 -> 285 -0.15860

Excited State 2: Singlet-A 1.4303 eV 866.81 nm f=0.1300
280 -> 284 -0.24297
281 -> 285 -0.31568
282 -> 285 0.42666
283 -> 284 0.40107

Excited State 3: Singlet-A 1.4573 eV 850.79 nm f=0.0113
280 -> 284 -0.38658
281 -> 285 0.40034
282 -> 285 -0.25278
283 -> 284 0.35221

Excited State 4: Singlet-B 1.4659 eV 845.77 nm f=0.0101
280 -> 285 -0.33874
281 -> 284 0.38820
283 -> 285 0.48102

Excited State 5: Singlet-B 1.4770 eV 839.43 nm f=0.0814
280 -> 285 0.41533
281 -> 284 0.16306
282 -> 284 0.54145
283 -> 285 0.10166

Excited State 6: Singlet-A 1.5109 eV 820.62 nm f=0.1370
280 -> 284 0.51272
281 -> 285 0.28173
282 -> 285 0.16412
283 -> 284 0.36503

Excited State 7: Singlet-A 1.5355 eV 807.45 nm f=0.0223
280 -> 284 -0.16286
281 -> 285 0.39949
282 -> 285 0.47866
283 -> 284 -0.29068

Excited State 8: Singlet-B 1.5361 eV 807.13 nm f=0.0071
280 -> 285 0.34824
281 -> 284 -0.24998
282 -> 284 -0.28251
283 -> 285 0.48545

Excited State 9: Singlet-B 2.3115 eV 536.39 nm f=0.0112
278 -> 284 0.51448
279 -> 285 0.45556

Excited State 10: Singlet-A 2.3124 eV 536.16 nm f=0.0006
278 -> 285 0.43962
279 -> 284 0.52902

Figure S13. Kohn-Sham molecular orbitals and its energies of dication state of **A-CH'** (**A-CH'**$^{2+}$, left), and TD-DFT calculated results (right). (B3LYP/6-31G**//ωB97X-D/6-31G**)

LUMO+4 (289)
α spin: -1.652 eV
β spin: -1.582 eV

LUMO+3 (288)
α spin: -1.660 eV
β spin: -1.594 eV

LUMO+2 (287)
α spin: -1.711 eV
β spin: -1.628 eV

LUMO+1 (286)
α spin: -1.716 eV
β spin: -1.629 eV

LUMO (285)
α spin: -4.293 eV
β spin: -2.614 eV

HOMO (284)
α spin: -4.338 eV
β spin: -2.647 eV

HOMO-1 (283)
α spin: -5.087 eV
β spin: -5.008 eV

HOMO-2 (282)
α spin: -5.134 eV
β spin: -5.057 eV

HOMO-3 (281)
α spin: -5.170 eV
β spin: -5.090 eV

HOMO-4 (280)
α spin: -5.176 eV
β spin: -5.096 eV

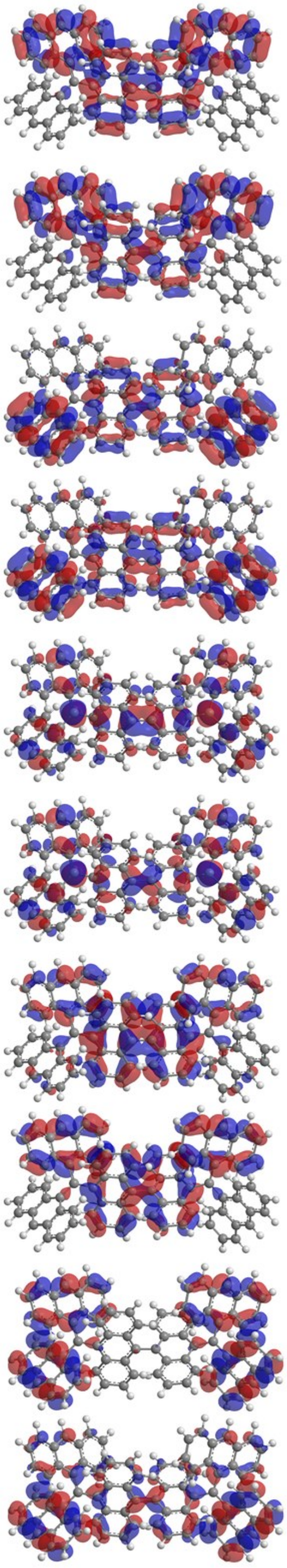

Figure S14. Kohn-Sham molecular orbitals and its energies of twisted triplet state of **A-CH’** (**A-CH’$^{2•}$**) (UB3LYP/6-31G**//UωB97X-D/6-31G**).

```
Excited State   1:  3.368-?Sym    1.4591 eV
        849.75 nm  f=0.0120  <S**2>=2.586
   280A -> 289A        0.10460
   282A -> 286A       -0.12593
   283A -> 287A        0.10984
   283A -> 291A       -0.10803
   284A -> 286A        0.22519
   284A -> 289A       -0.25516
   285A -> 287A        0.27068
   285A -> 288A       -0.28080
   280B -> 285B       -0.26083
   280B -> 289B        0.10582
   282B -> 285B       -0.40295
   282B -> 286B       -0.11938
   283B -> 284B        0.57692
   283B -> 287B        0.12390
```

```
Excited State   2:  3.371-?Sym    1.4699 eV
        843.50 nm  f=0.0006  <S**2>=2.591
   280A -> 288A        0.10436
   282A -> 287A       -0.12582
   283A -> 286A        0.11448
   283A -> 290A        0.10392
   284A -> 287A        0.25059
   284A -> 288A       -0.26210
   285A -> 286A        0.27305
   285A -> 289A       -0.30209
   280B -> 284B       -0.27934
   280B -> 288B        0.10334
   282B -> 284B       -0.43078
   282B -> 287B       -0.12132
   283B -> 285B        0.52022
   283B -> 286B        0.10996
```

```
Excited State   3:  3.350-?Sym    1.4952 eV
        829.20 nm  f=0.0039  <S**2>=2.555
   281A -> 290A       -0.11800
   282A -> 288A       -0.11477
   284A -> 287A       -0.31100
   284A -> 288A       -0.24372
   285A -> 286A       -0.32825
   285A -> 289A       -0.23161
   280B -> 284B        0.42071
   280B -> 287B       -0.10467
   281B -> 285B        0.49291
   282B -> 284B       -0.30848
   282B -> 288B       -0.11553
   283B -> 285B        0.10180
```

```
Excited State   4:  3.350-?Sym    1.4953 eV
        829.18 nm  f=0.0001  <S**2>=2.555
   280A -> 290A        0.10371
   281A -> 291A        0.11058
   282A -> 289A       -0.10971
   283A -> 288A        0.10392
   284A -> 286A       -0.32399
   284A -> 289A       -0.22762
   285A -> 287A       -0.31944
   285A -> 288A       -0.24158
   280B -> 285B        0.40759
   281B -> 284B        0.50845
   282B -> 285B       -0.30077
   282B -> 289B       -0.10760
   283B -> 284B        0.10400
```

```
Excited State   5:  3.732-?Sym    1.7885 eV
        693.22 nm  f=0.0009  <S**2>=3.231
   280A -> 286A        0.24490
   281A -> 287A       -0.21800
   281A -> 288A       -0.12278
   282A -> 289A       -0.23466
   283A -> 287A       -0.12749
   283A -> 288A        0.21499
   284A -> 290A       -0.37789
   285A -> 291A        0.39118
   278B -> 285B       -0.28201
   279B -> 284B       -0.29748
   280B -> 286B        0.24697
   281B -> 287B        0.20057
   281B -> 288B        0.15579
   282B -> 289B       -0.24312
   283B -> 287B       -0.15907
   283B -> 288B        0.20376
```

```
Excited State   6:  3.730-?Sym    1.7902 eV
        692.56 nm  f=0.0005  <S**2>=3.229
   280A -> 287A       -0.24231
   281A -> 286A        0.22227
   281A -> 289A        0.11402
   282A -> 288A        0.23342
   283A -> 286A        0.11812
   283A -> 289A       -0.21792
   284A -> 291A       -0.36776
   285A -> 290A        0.40087
   278B -> 284B        0.29343
   279B -> 285B        0.28673
   280B -> 287B       -0.24209
   281B -> 286B       -0.22045
   281B -> 289B       -0.12499
   282B -> 288B        0.24372
   283B -> 286B        0.12354
   283B -> 289B       -0.21908
```

```
Excited State   7:  3.155-?Sym    1.9252 eV
        644.01 nm  f=0.3995  <S**2>=2.239
   284A -> 286A       -0.27371
   284A -> 289A        0.36686
   285A -> 287A       -0.36292
   285A -> 288A        0.43301
   280B -> 285B       -0.15811
   281B -> 284B       -0.10582
   282B -> 285B       -0.18768
   283B -> 284B        0.58008
```

```
Excited State   8:  3.148-?Sym    2.0122 eV
        616.17 nm  f=0.0132  <S**2>=2.228
   284A -> 287A       -0.11083
   284A -> 288A        0.26303
   285A -> 286A       -0.14200
   285A -> 289A        0.31374
   280B -> 284B        0.15542
   282B -> 284B        0.20469
   282B -> 287B        0.11464
   283B -> 285B        0.82377
```

```
Excited State   9:  3.127-?Sym    2.0224 eV
        613.05 nm  f=0.0275  <S**2>=2.195
   284A -> 286A        0.37536
   284A -> 289A        0.29791
   285A -> 287A        0.39433
   285A -> 288A        0.36210
   280B -> 285B        0.30393
   281B -> 284B        0.49949
   282B -> 285B       -0.29863
```

```
Excited State  10:  3.146-?Sym    2.0241 eV  612.53 nm  f=0.1535  <S**2>=2.224
   284A -> 287A       -0.15570
   284A -> 288A       -0.38168
   285A -> 286A       -0.16519
   285A -> 289A       -0.43874
   281B -> 285B       -0.27361
   282B -> 284B        0.67912
   283B -> 285B        0.10729
   283B -> 286B        0.11648
```

, **Figure S15**. TD-DFT calculated results of **A-CH'**$^{2\bullet}$. (UB3LYP/6-31G**//UωB97X-D/6-31G**)

Relative energy differences of A-TH conformers

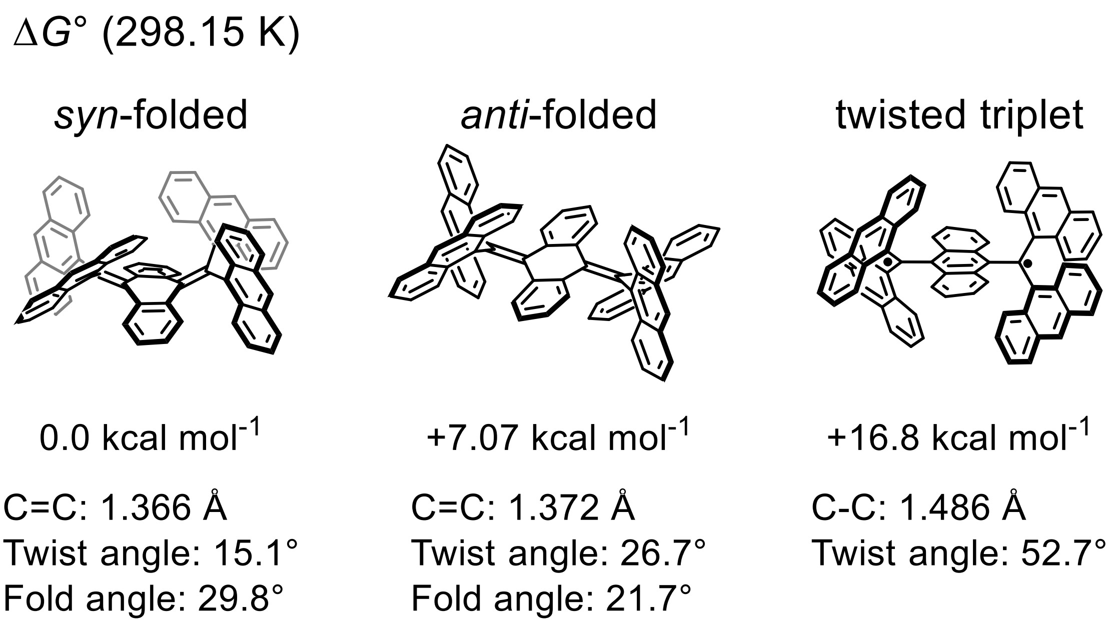

Figure S16. Gibbs free energy differences of *syn*-folded, *anti*-folded, and twisted triplet of **A-TH'**. ((U)ωB97X-D/6-31G**)

UV-vis NIR spectra of TAntM cation and TAntM radical

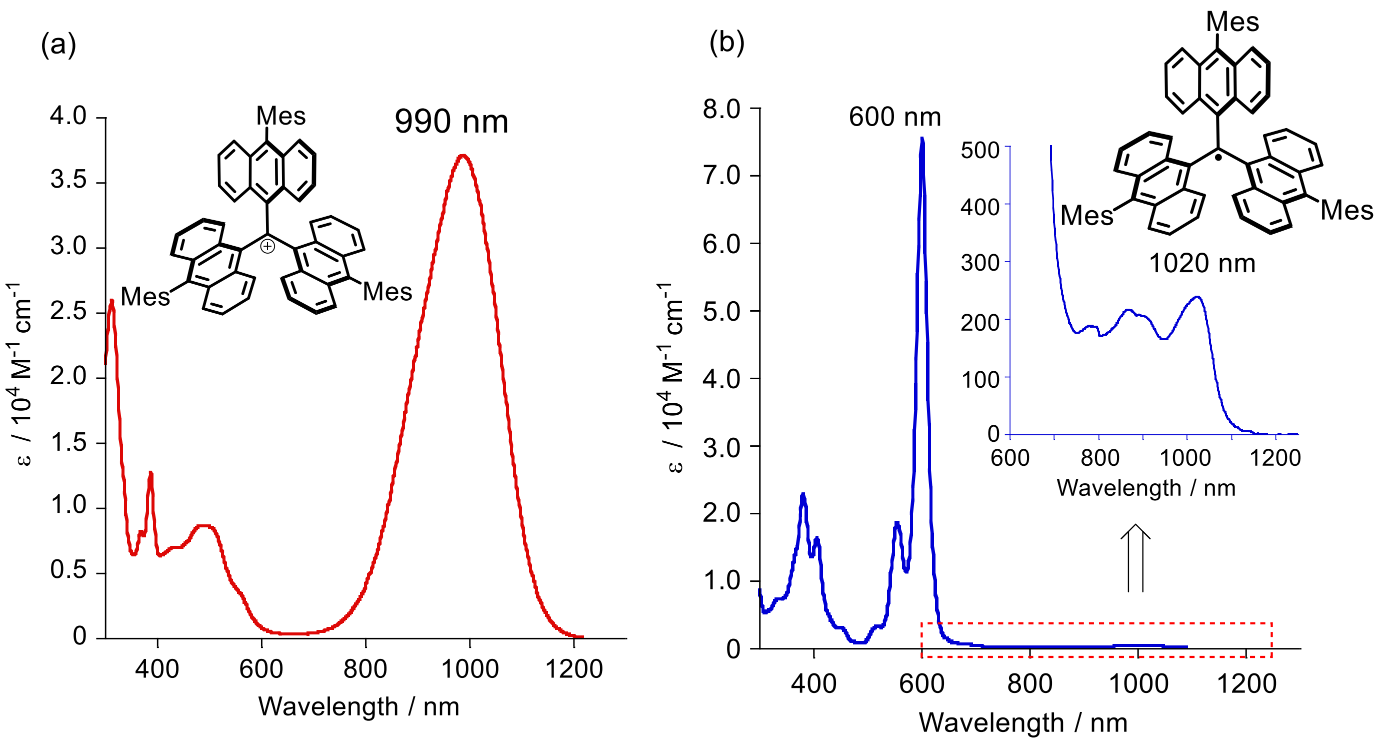

Figure S17. (a) UV-vis-NIR spectrum of **TAntM** cation. (b) UV-vis-NIR spectrum of **TAntM** radical.

Spin density map of twisted triplet state of A-CH'[2•]

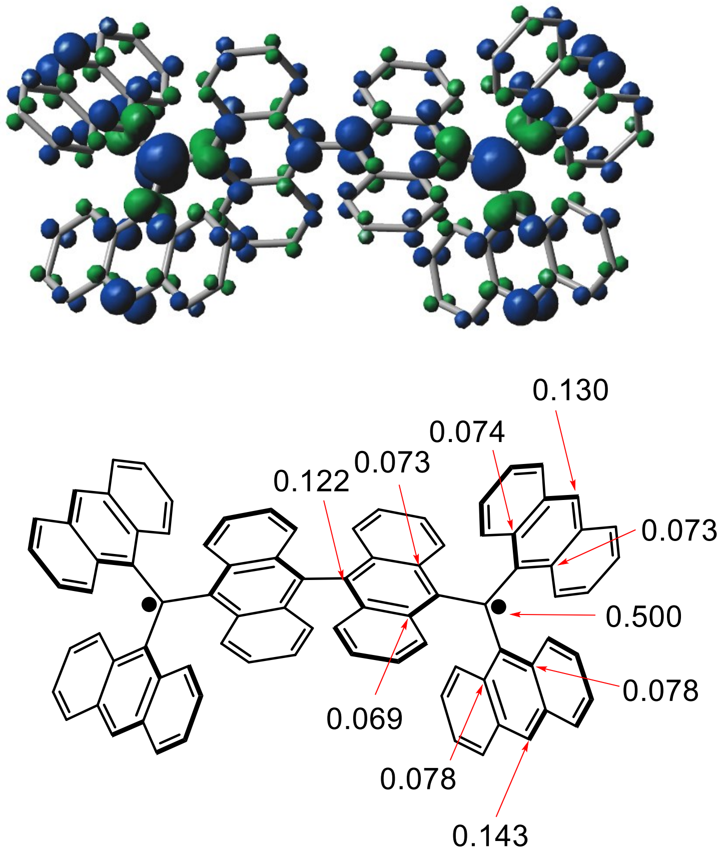

Figure S18. Spin density map andspin density values (α spin) of triplet state of **A-CH'**[2•] (UBLYP/6-31G**//UωB97X-D/6-31G**).

Decay plots of UV-vis absorption of A-CH$^{2•}$ at 5, 0 –5, –10, and –15 °C

(a)

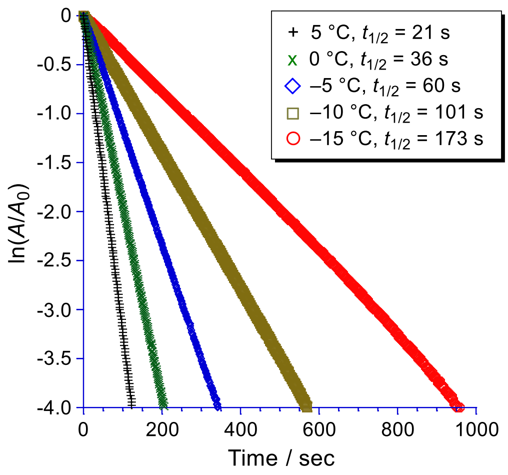

(b)

5 °C

	k / 10^{-2} s	ln(k/T)
1st.	3.338	-9.027
2nd.	3.237	-9.058
3rd.	3.236	-9.058
Ave.	3.270	-9.048

0 °C

	k / 10^{-2} s	ln(k/T)
1st.	1.921	-9.562
2nd.	1.916	-9.564
3rd.	1.941	-9.551
Ave.	1.926	-9.559

–5 °C

	k / 10^{-2} s	ln(k/T)
1st.	1.164	-10.044
2nd.	1.164	-10.044
3rd.	1.167	-10.042
Ave.	1.165	-10.043

–10 °C

	k / 10^{-3} s	ln(k/T)
1st.	6.767	-10.568
2nd.	6.943	-10.542
3rd.	6.964	-10.539
Ave.	6.891	-10.550

–15 °C

	k / 10^{-3} s	ln(k/T)
1st.	4.021	-11.069
2nd.	4.072	-11.057
3rd.	3.959	-11.085
Ave.	4.017	-11.070

Figure S19. (a) Decay plots of UV-vis absorption of **A-CH**$^{2•}$ at 5, 0 –5, –10, and –15 °C (monitored at λ_{abs} = 615 nm), and its half-life. (b) Three-times averages of the rate constants and ln(k/T) of each temperatures.

ESR spectrum of folded A-CH after ground

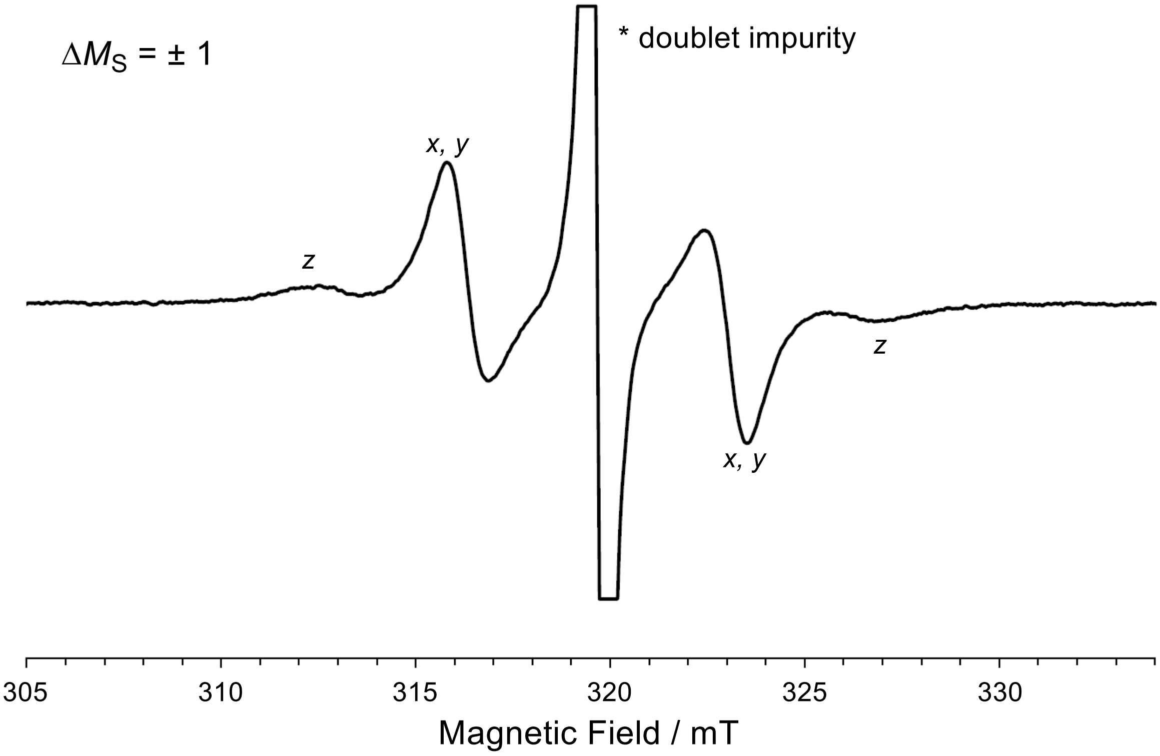

Figure S20. ESR spectrum of folded **A-CH** after ground, dissolve in cold toluene. The spectrum was measured at –73 °C.

Solid-state diffuse-reflection UV-vis-NIR spectra of A-CH after ground

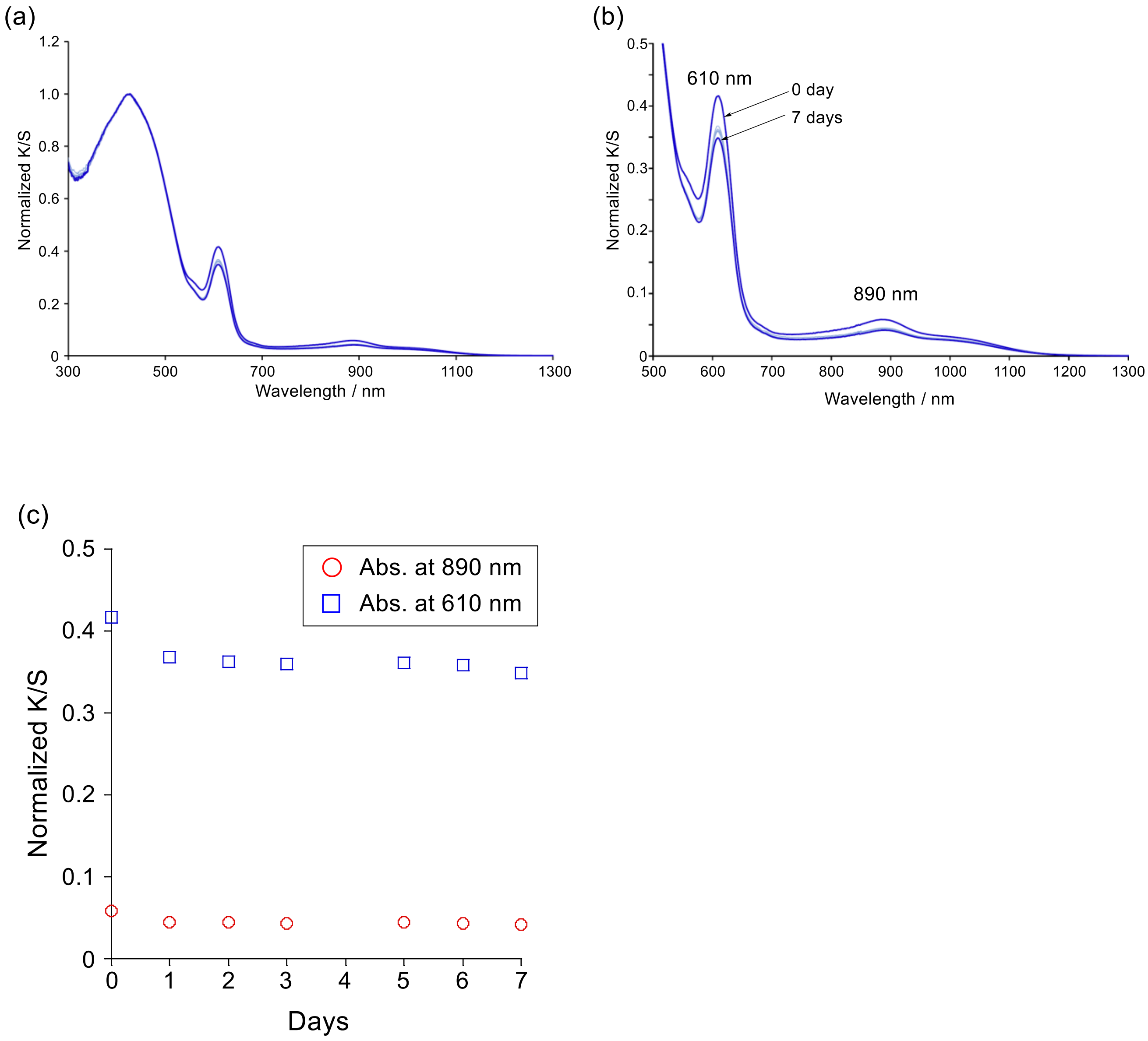

Figure S21. (a) Solid-state diffuse-reflection UV-vis-NIR spectra and solid-phase decay of folded **A-CH** after grinding (~7 days). (b) Magnified view of (a) from 500 nm to 1300 nm. (c) Decay plot of after grinding of **A-CH** at 890 nm and 610 nm.

X-ray crystallographic data

Crystal data for A-TH. CCDC 2149926. $C_{108}H_{84}$, M_W = 1381.75, monoclinic, space group *P* c (no. 7), *a* = 27.7003(15), *b* = 8.2413(4), *c* = 39.418(3) Å, β = 107.839(7)°, *V* = 8566.1(10) Å^3, *Z* = 4, *D* = 1.071 g cm^3, *T* = 93 K. Crystal size: 0.172 x 0.064 x 0.004 mm. Data collection with CuKα radiation (multi-layer mirror monochromated). 23691 measured reflection, 13386 unique reflections; structure solution by direct methods (Shelxs), refinement by fullmatrix least squares on *F* with anisotropic temperature factors for the non-H atoms. Final R_1 = 0.1736, wR_2 = 0.4388, *GOF* = 1.346.

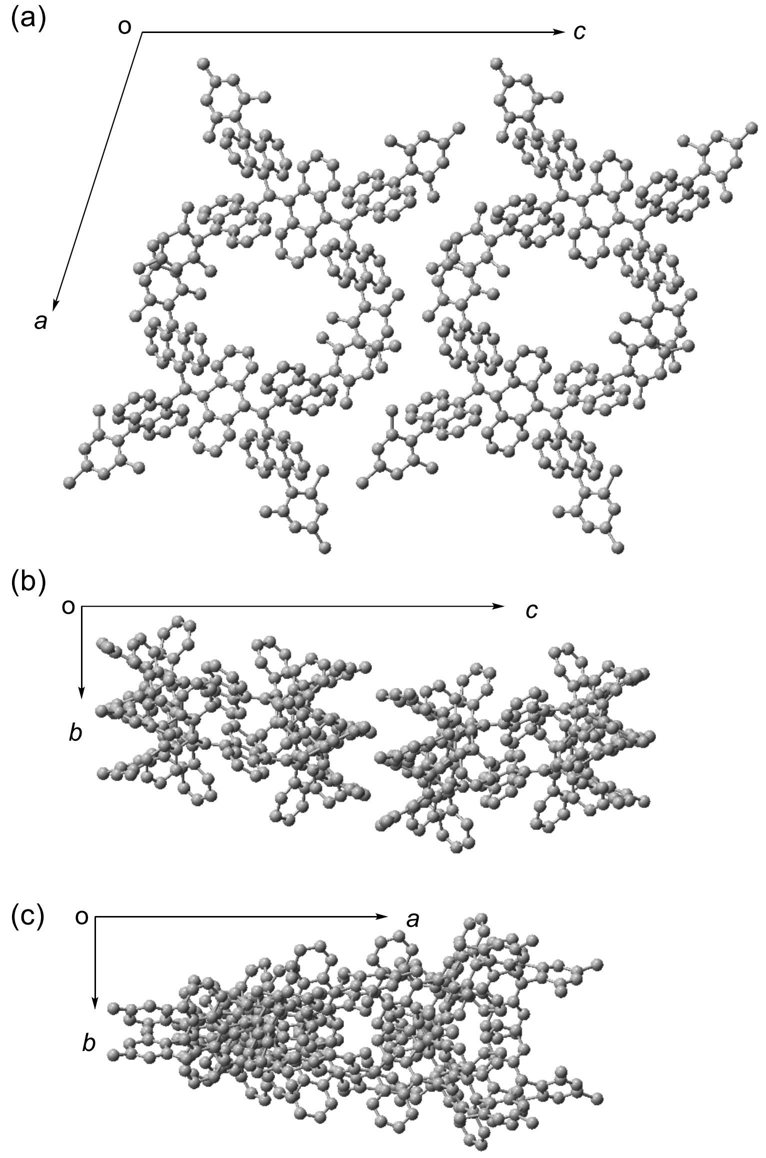

Figure S22. Packing structures of **A-TH**. (a) Viewing from *b* axis. (b) Viewing from *a* axis. (c) Viewing from *c* axis. Protons are omitted for clarity. Solvent mask was used due to disordering of recrystallized solvent.

Crystal data for A-CH. CCDC 2149927. $C_{122}H_{92}$, M_W = 1557.95, monoclinic, space group $P\ 2_1/c$ (no. 14), a = 19.5547(6), b = 31.2994(9), c = 8.1695(3) Å, β = 97.322(3)°, V = 4959.4(3) Å^3, Z = 2, D = 1.043 g cm^3, T = 120 K. Crystal size: 0.40 x 0.04 x 0.02 mm. Data collection with MoKα radiation (multi-layer mirror monochromated). 12489 measured reflection, 6981 unique reflections; structure solution by direct methods (Shelxs), refinement by fullmatrix least squares on F with anisotropic temperature factors for the non-H atoms. Final R_1 = 0.0552, wR_2 = 0.1432, GOF = 1.003.

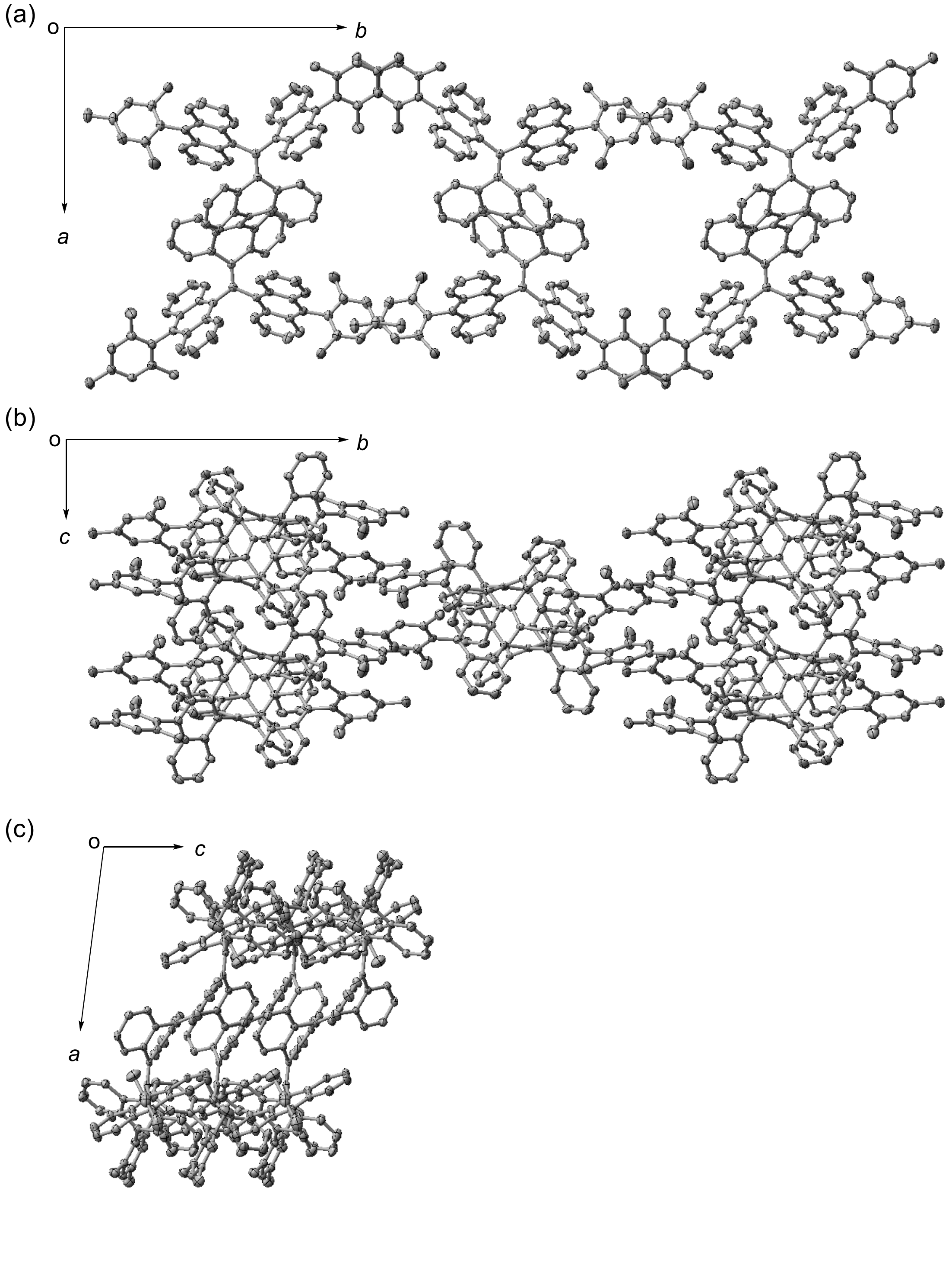

Figure S23. Packing structures of **A-CH**. (a) Viewing from c axis. (b) Viewing from a axis. (c) Viewing from b axis. Protons are omitted for clarity. Solvent mask was used due to disordering of recrystallized solvent.

NMR spectra

^{1}H NMR (600 MHz) of compound **A-TH** at 80 °C($C_2D_2Cl_4$).

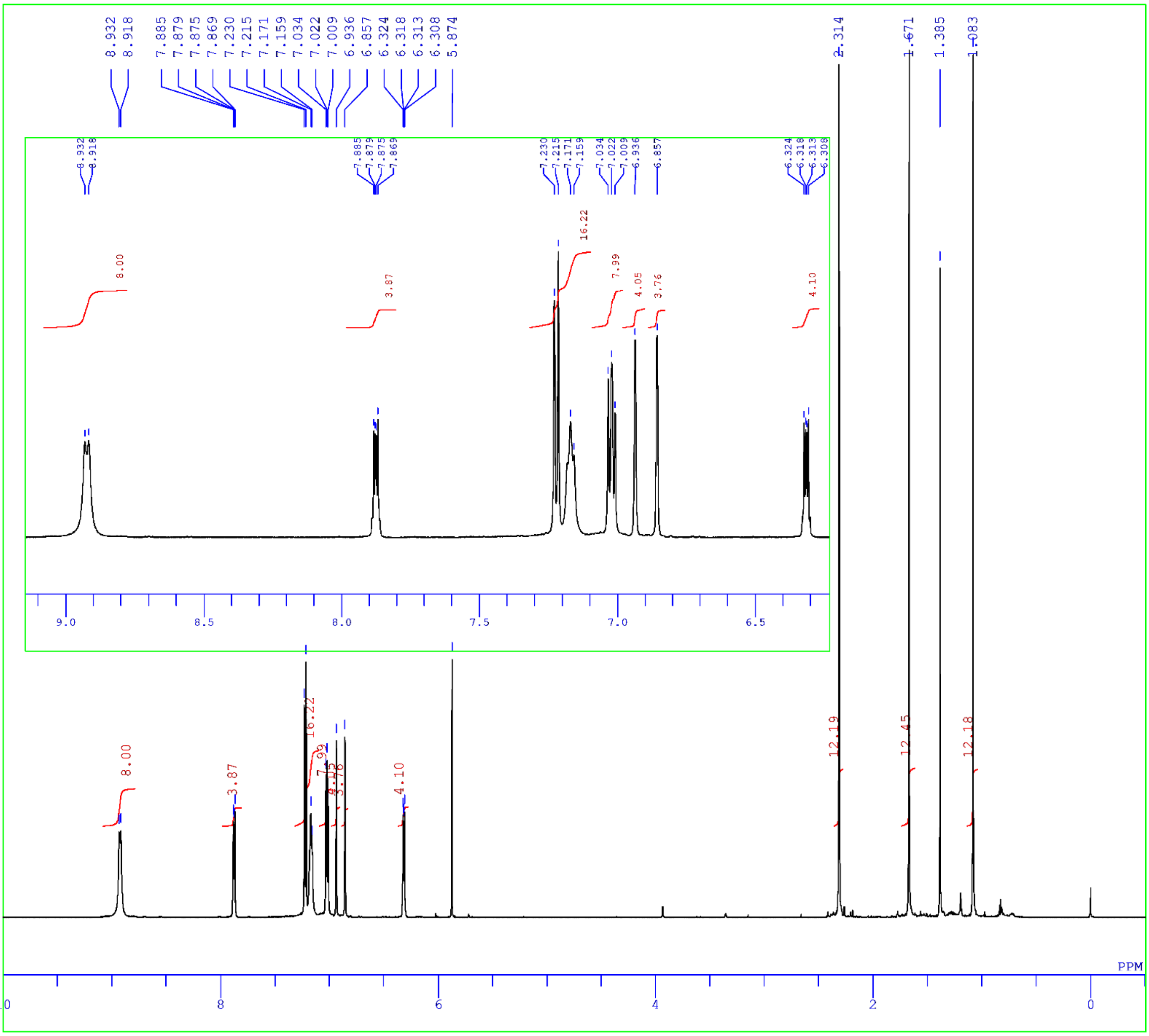

^{13}C NMR (150 MHz) of **A-TH** at 80 °C ($C_2D_2Cl_4$).

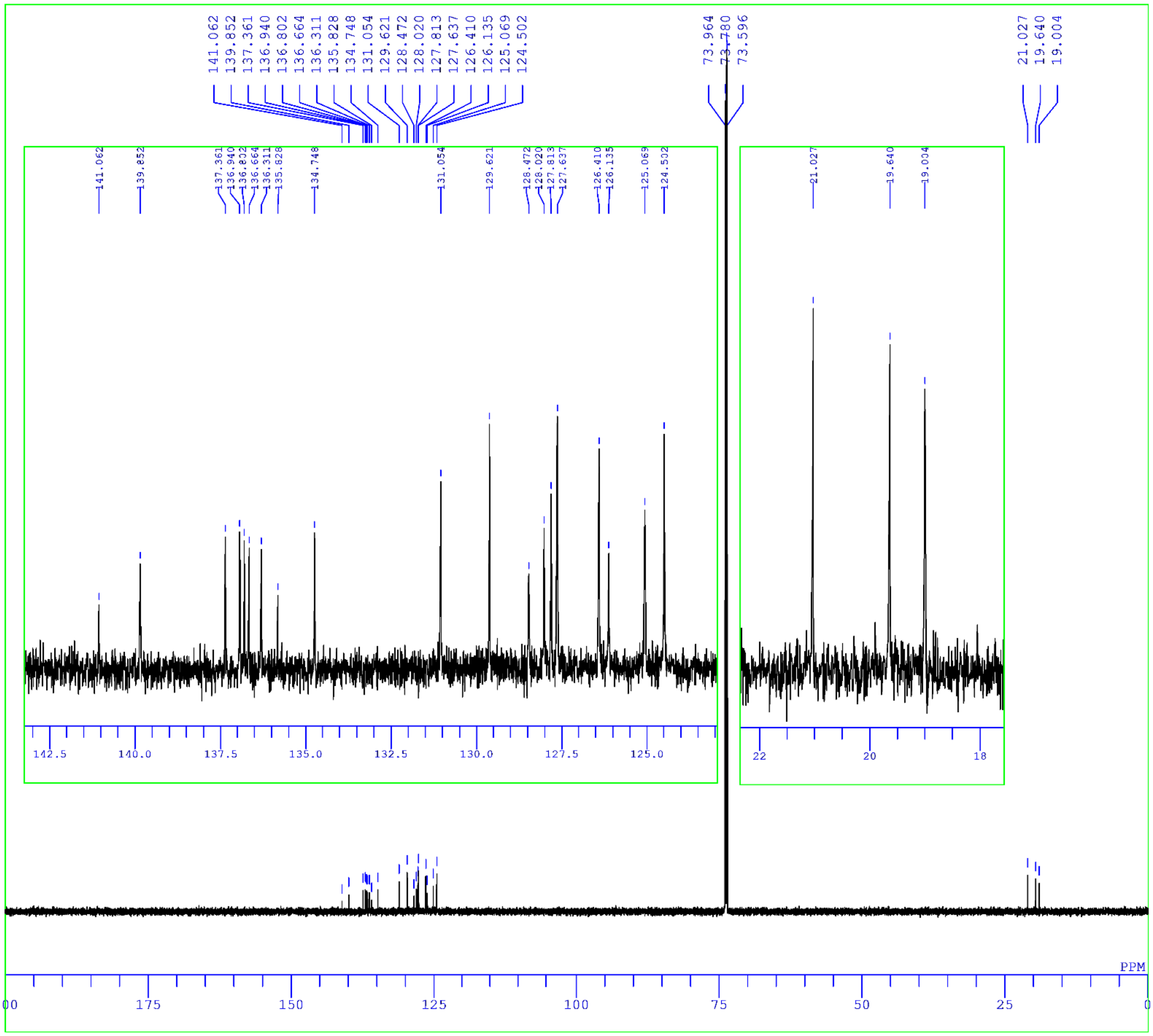

^{1}H NMR (400 MHz) of compound **A-CH** at 80 °C($C_2D_2Cl_4$).

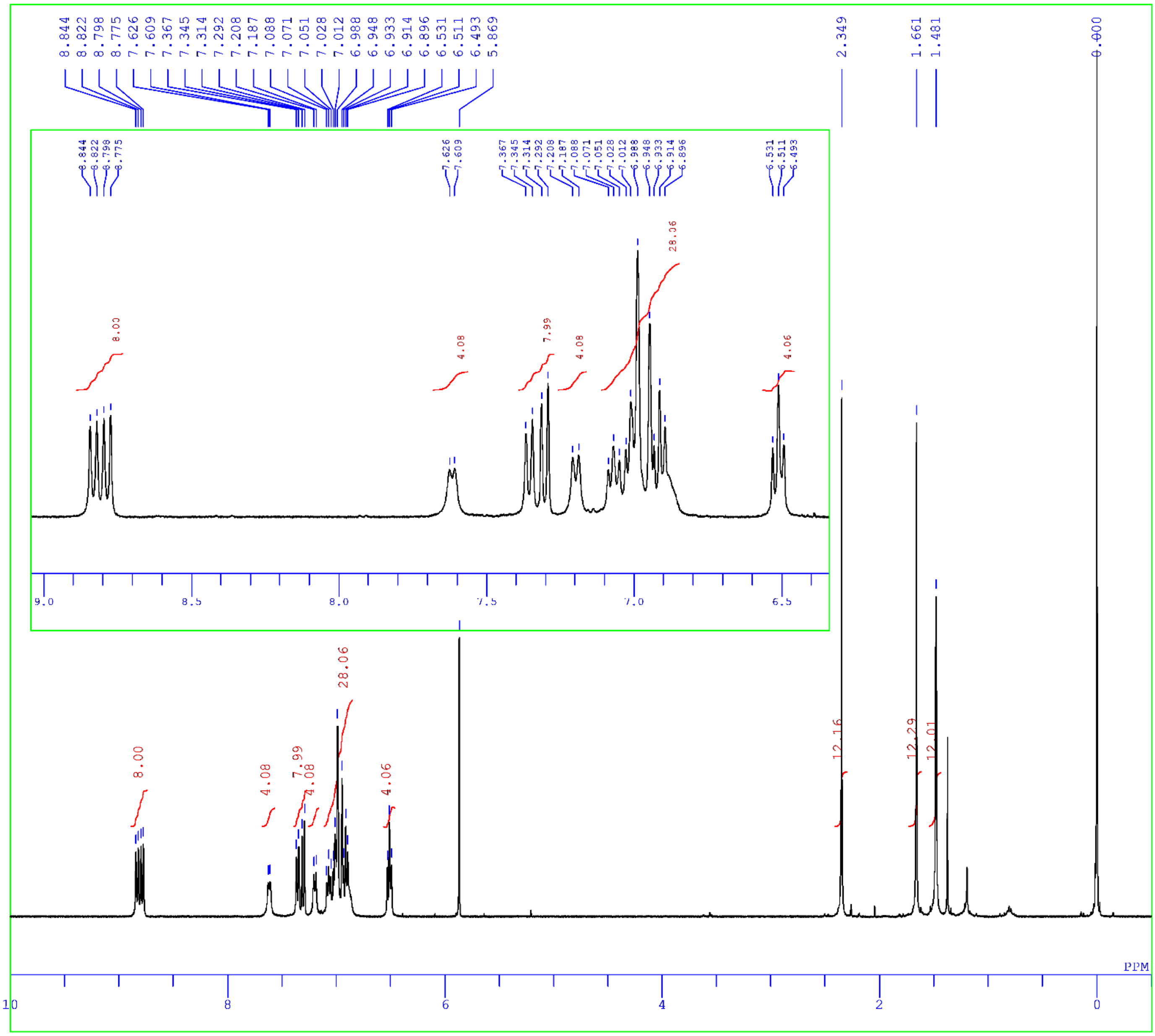

^{13}C NMR (100 MHz) of compound **A-CH** at 80 °C ($C_2D_2Cl_4$).

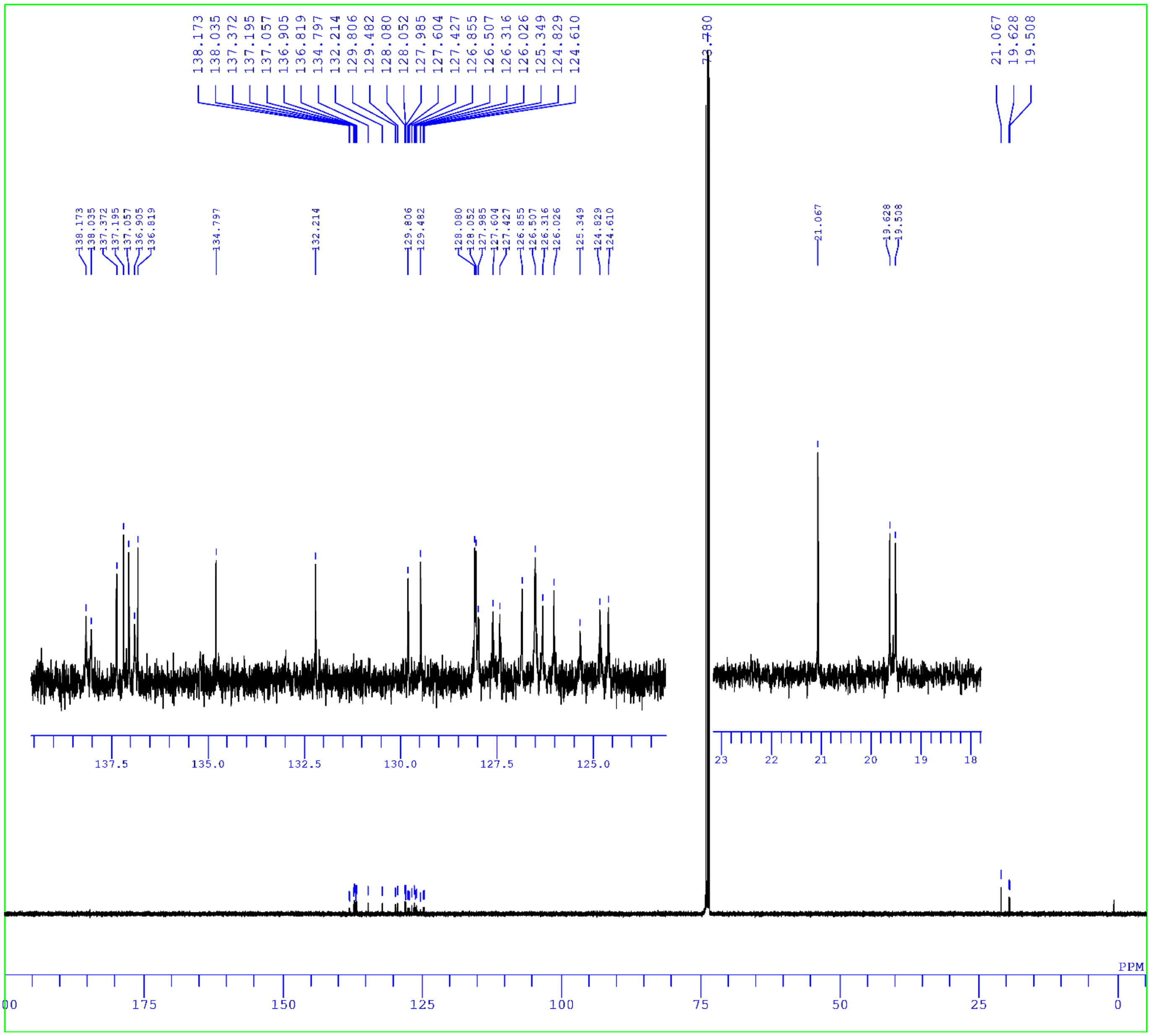

^{1}H NMR (400 MHz) of compound **A-CH^{2+}** (CD_2Cl_2).

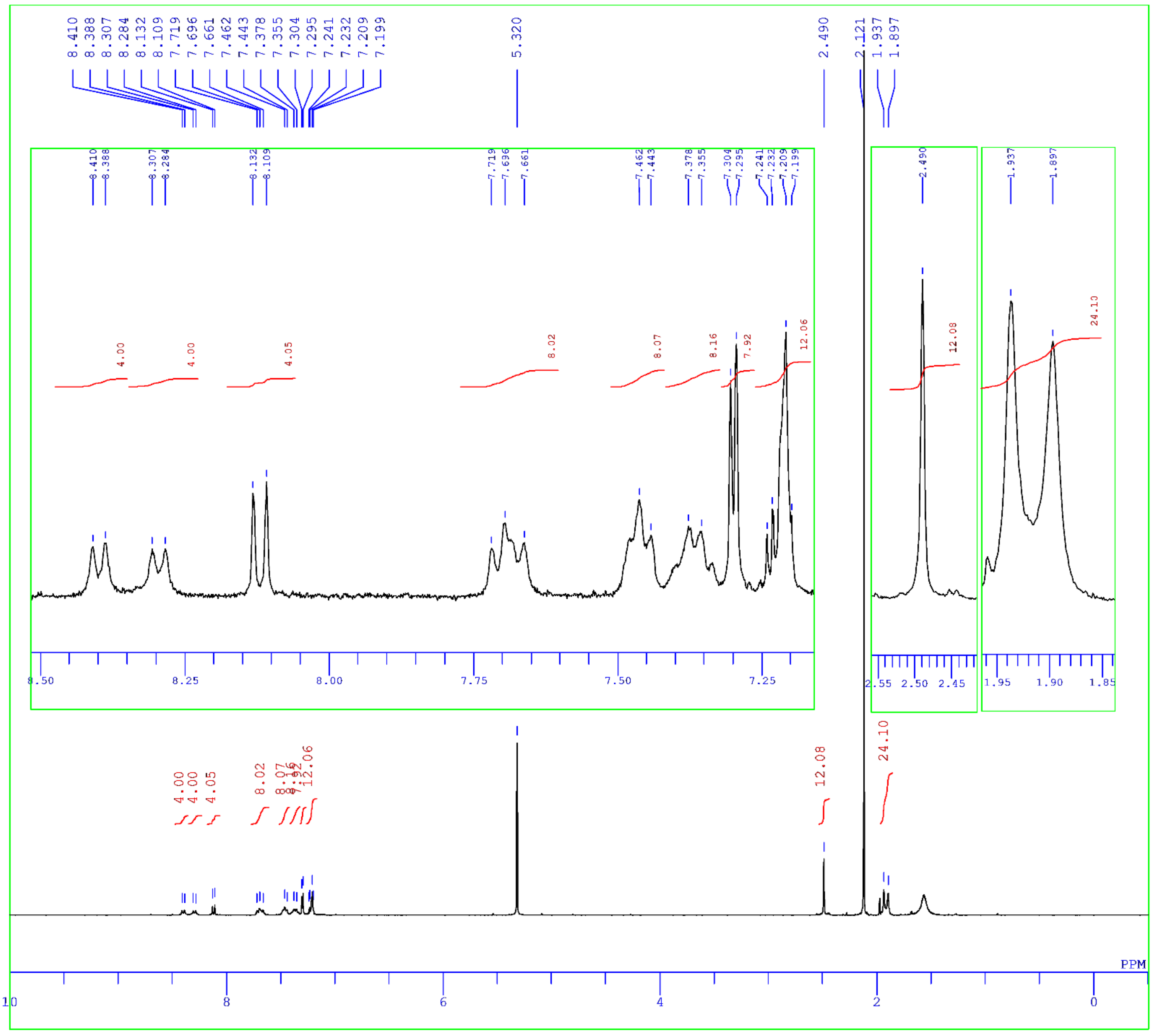

^{13}C NMR (100 MHz) of compound **A-CH^{2+}**

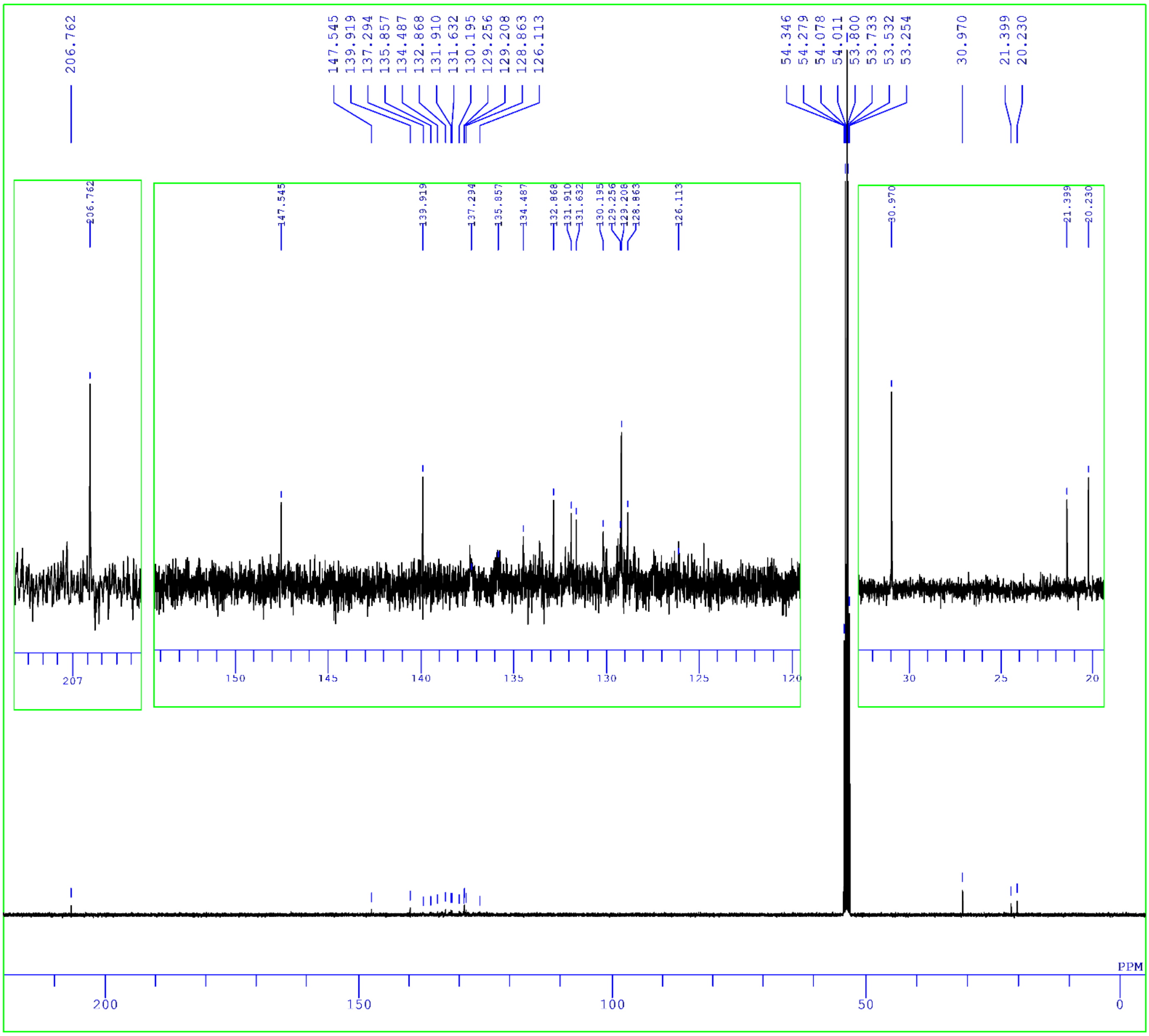

Table S1. Cartesian coordinate of **A-TH** *syn*-folded (ωB97X-D/6-31G**).

Center Number	Atomic Number	Atomic Type	X	Y	Z
1	6	0	0.683044	1.206396	-1.524214
2	6	0	-0.726782	1.271934	-1.434299
3	6	0	-1.435862	0.003517	-1.088488
4	6	0	-0.683091	-1.206475	-1.524147
5	6	0	0.726735	-1.272007	-1.434221
6	6	0	1.435815	-0.003571	-1.088481
7	6	0	1.394448	2.296584	-2.034813
8	6	0	0.764163	3.489514	-2.347075
9	6	0	-0.603959	3.600605	-2.140778
10	6	0	-1.335153	2.504561	-1.704902
11	6	0	-1.394492	-2.296700	-2.034670
12	6	0	-0.764202	-3.489649	-2.346851
13	6	0	0.603920	-3.600725	-2.140545
14	6	0	1.335109	-2.504651	-1.704740
15	6	0	2.622067	0.145634	-0.427681
16	6	0	5.241402	-2.797832	2.668779
17	6	0	4.951893	-2.363852	1.336726
18	6	0	3.901928	-1.411937	1.113496
19	6	0	3.175623	-0.960624	2.260392
20	6	0	3.478889	-1.395611	3.516211
21	6	0	4.532259	-2.327565	3.730703
22	6	0	5.676694	-2.853738	0.254548
23	6	0	5.387119	-2.456105	-1.047220
24	6	0	4.336645	-1.509094	-1.284638
25	6	0	3.608181	-0.970050	-0.195583
26	6	0	6.125479	-2.986819	-2.153231
27	6	0	5.839186	-2.623031	-3.431973
28	6	0	4.782946	-1.701303	-3.680831
29	6	0	4.064679	-1.168661	-2.653597
30	6	0	6.770546	3.314834	-0.813531
31	6	0	6.035724	3.787648	0.229661
32	6	0	4.774082	3.206941	0.571112
33	6	0	4.288367	2.071526	-0.160140
34	6	0	5.076644	1.647184	-1.275949
35	6	0	6.264294	2.237953	-1.590809
36	6	0	4.001793	3.750375	1.591619
37	6	0	2.750841	3.228924	1.899137
38	6	0	2.263077	2.075923	1.195516
39	6	0	3.053552	1.468263	0.185762
40	6	0	1.952003	3.833119	2.921497
41	6	0	0.727979	3.337962	3.245307
42	6	0	0.236952	2.191439	2.563099
43	6	0	0.974017	1.592047	1.587688
44	6	0	-3.175504	0.960877	2.260350
45	6	0	-3.478718	1.395969	3.516145
46	6	0	-4.532104	2.327912	3.730608
47	6	0	-5.241307	2.798070	2.668676
48	6	0	-3.608209	0.970045	-0.195605
49	6	0	-3.901888	1.412054	1.113449
50	6	0	-4.951853	2.363978	1.336646
51	6	0	-5.676706	2.853769	0.254462
52	6	0	-4.783103	1.701036	-3.680864
53	6	0	-4.064796	1.168475	-2.653615
54	6	0	-4.336714	1.509013	-1.284673
55	6	0	-5.387180	2.456038	-1.047287
56	6	0	-6.125582	2.986666	-2.153311
57	6	0	-5.839336	2.622779	-3.432036
58	6	0	-5.076662	-1.647350	-1.275841
59	6	0	-6.264303	-2.238168	-1.590642
60	6	0	-6.770533	-3.314992	-0.813273
61	6	0	-6.035688	-3.787710	0.229947
62	6	0	-3.053566	-1.468259	0.185862
63	6	0	-4.288369	-2.071576	-0.159999
64	6	0	-4.774053	-3.206953	0.571338
65	6	0	-4.001742	-3.750311	1.591868
66	6	0	-0.727896	-3.337754	3.245460
67	6	0	-0.236903	-2.191262	2.563176
68	6	0	-0.974002	-1.591940	1.587750
69	6	0	-2.263064	-2.075860	1.195633
70	6	0	-2.750791	-3.228827	1.899330
71	6	0	-1.951920	-3.832950	2.921706
72	6	0	-2.622101	-0.145657	-0.427661
73	1	0	2.463429	2.206564	-2.182791
74	1	0	1.339022	4.322316	-2.738228
75	1	0	-1.120207	4.532236	-2.348550
76	1	0	-2.398293	2.623042	-1.597112
77	1	0	-2.463474	-2.206696	-2.182655
78	1	0	-1.339061	-4.322478	-2.737949
79	1	0	1.120169	-4.532370	-2.348254
80	1	0	2.398250	-2.623118	-1.596933
81	1	0	6.043342	-3.516778	2.810725
82	1	0	2.361700	-0.263150	2.128664
83	1	0	2.906304	-1.027834	4.362135
84	1	0	4.760509	-2.661957	4.737485
85	1	0	6.479108	-3.566593	0.428106
86	1	0	6.921788	-3.695244	-1.942952
87	1	0	6.403278	-3.034479	-4.262486
88	1	0	4.542921	-1.425504	-4.702600
89	1	0	3.251300	-0.485466	-2.870120
90	1	0	7.723904	3.766471	-1.067923
91	1	0	6.385887	4.630330	0.819266
92	1	0	4.712095	0.854724	-1.908817
93	1	0	6.823782	1.885615	-2.451595
94	1	0	4.373795	4.609265	2.144864
95	1	0	2.351539	4.705953	3.430332
96	1	0	0.125259	3.806889	4.016111
97	1	0	-0.741803	1.798993	2.813652
98	1	0	0.574185	0.726123	1.084579
99	1	0	-2.361557	0.263428	2.128644
100	1	0	-2.906075	1.028287	4.362072
101	1	0	-4.760312	2.662389	4.737370
102	1	0	-6.043253	3.517014	2.810595
103	1	0	-6.479112	3.566638	0.427996
104	1	0	-4.543118	1.425158	-4.702621
105	1	0	-3.251420	0.485268	-2.870112
106	1	0	-6.921885	3.695105	-1.943058
107	1	0	-6.403461	3.034161	-4.262560
108	1	0	-4.712132	-0.854944	-1.908785
109	1	0	-6.823801	-1.885909	-2.451455
110	1	0	-7.723884	-3.766666	-1.067619
111	1	0	-6.385825	-4.630355	0.819620
112	1	0	-4.373724	-4.609177	2.145165
113	1	0	-0.125151	-3.806631	4.016274
114	1	0	0.741851	-1.798783	2.813685
115	1	0	-0.574198	-0.726044	1.084569
116	1	0	-2.351429	-4.705763	3.430598

No imaginary frequencies.
Zero-point correction= 0.937654 (Hartree/Particle)
Thermal correction to Energy= 0.988752
Thermal correction to Enthalpy= 0.989696
Thermal correction to Gibbs Free Energy= 0.853396
Sum of electronic and zero-point Energies= -2768.420409
Sum of electronic and thermal Energies= -2768.369311
Sum of electronic and thermal Enthalpies= -2768.368367
Sum of electronic and thermal Free Energies= -2768.504667

Table S2. Cartesian coordinate of **A-TH** *anti*-folded (ωB97X-D/6-31G**).

Center Number	Atomic Number	Atomic Type	Coordinates (Angstroms) X	Y	Z
1	6	0	-0.665594	1.285092	0.214311
2	6	0	-0.029531	1.263167	-4.039187
3	6	0	-4.151349	3.625001	-2.284651
4	6	0	-0.885523	1.462190	-5.157022
5	6	0	-0.146263	4.845958	0.024590
6	6	0	-1.169398	2.525063	-0.085174
7	6	0	0.080633	6.069980	0.738853
8	6	0	-0.910041	5.103176	2.733772
9	6	0	0.350149	4.764922	-1.315655
10	6	0	-3.346573	3.366234	-1.125627
11	6	0	-3.710219	3.205474	-3.535740
12	6	0	-0.396224	1.674650	-2.792874
13	6	0	-2.496447	2.542478	-3.689399
14	6	0	-1.659134	2.312058	-2.547089
15	6	0	-1.846598	4.169560	4.774704
16	6	0	-5.124459	4.321304	0.249028
17	6	0	0.792155	-3.765236	-0.662282
18	6	0	-1.152426	3.876713	2.030684
19	6	0	-2.092875	2.726227	-1.259429
20	6	0	-3.908194	3.713988	0.145001
21	6	0	-2.076747	2.094754	-4.982191
22	6	0	-0.314540	6.172973	2.068911
23	6	0	2.828247	1.904077	1.610516
24	6	0	-1.277424	5.212924	4.111898
25	6	0	-3.507496	-0.852444	-1.005571
26	6	0	-0.792155	3.765236	0.662282
27	6	0	0.971499	5.822779	-1.908228
28	6	0	-1.755907	2.806986	2.772248
29	6	0	-5.887555	4.629323	-0.910861
30	6	0	-5.413962	4.280939	-2.138213
31	6	0	-2.828247	-1.904077	-1.610516
32	6	0	2.092875	-2.726227	1.259429
33	6	0	1.659134	-2.312058	2.547089
34	6	0	-2.086959	2.947745	4.087369
35	6	0	1.456723	2.023725	1.456563
36	6	0	0.726136	7.162555	0.078205
37	6	0	0.314540	-6.172973	-2.068911
38	6	0	-2.777619	0.128985	-0.361544
39	6	0	0.705833	1.071585	0.747677
40	6	0	1.376619	-0.091324	0.304765
41	6	0	-0.726136	-7.162555	-0.078205
42	6	0	2.496447	-2.542478	3.689399
43	6	0	-1.376619	0.091324	-0.304765
44	6	0	1.169398	-2.525063	0.085174
45	6	0	1.152426	-3.876713	-2.030684
46	6	0	0.665594	-1.285092	-0.214311
47	6	0	-0.705833	-1.071585	-0.747677
48	6	0	3.507496	0.852444	1.005571
49	6	0	3.710219	-3.205474	3.535740
50	6	0	0.146263	-4.845958	-0.024590
51	6	0	3.908194	-3.713988	-0.145001
52	6	0	-0.971499	-5.822779	1.908228
53	6	0	1.277424	-5.212924	-4.111898
54	6	0	1.153664	7.048785	-1.208434
55	6	0	-0.350149	-4.764922	1.315655
56	6	0	4.151349	-3.625001	2.284651
57	6	0	-0.080633	-6.069980	-0.738853
58	6	0	2.777619	-0.128985	0.361544
59	6	0	0.396224	-1.674650	2.792874
60	6	0	0.910041	-5.103176	-2.733772
61	6	0	0.885523	-1.462190	5.157022
62	6	0	3.346573	-3.366234	1.125627
63	6	0	1.755907	-2.806986	-2.772248
64	6	0	2.076747	-2.094754	4.982191
65	6	0	-1.456723	-2.023725	-1.456563
66	6	0	0.029531	-1.263167	4.039187
67	6	0	5.413962	-4.280939	2.138213
68	6	0	1.846598	-4.169560	-4.774704
69	6	0	-1.153664	-7.048785	1.208434
70	6	0	2.086959	-2.947745	-4.087369
71	6	0	5.124459	-4.321304	-0.249028
72	6	0	5.887555	-4.629323	0.910861
73	1	0	0.930889	0.777303	-4.179386
74	1	0	-0.579831	1.121041	-6.140698
75	1	0	0.246385	3.839719	-1.863304
76	1	0	0.291556	1.519027	-1.972156
77	1	0	-2.117991	4.263034	5.821108
78	1	0	-5.517961	4.566475	1.230389
79	1	0	-3.366481	3.475110	1.048218
80	1	0	-2.739656	2.273469	-5.824165
81	1	0	3.367350	2.660825	2.171001
82	1	0	-1.085267	6.154102	4.619356
83	1	0	-4.589370	-0.786624	-1.049197
84	1	0	1.339632	5.727236	-2.924624
85	1	0	-1.946203	1.864595	2.273823
86	1	0	-6.848831	5.122754	-0.810178
87	1	0	-5.992620	4.483823	-3.035092
88	1	0	-3.367350	-2.660825	-2.171001
89	1	0	-2.541012	2.116670	4.617886
90	1	0	0.972992	2.879135	1.896504
91	1	0	0.874039	8.084014	0.634269
92	1	0	-3.297171	0.960029	0.098962
93	1	0	-0.874039	-8.084014	-0.634269
94	1	0	4.589370	0.786624	1.049197
95	1	0	3.366481	-3.475110	-1.048218
96	1	0	-1.339632	-5.727236	2.924624
97	1	0	1.085267	-6.154102	-4.619356
98	1	0	1.645096	7.881908	-1.700502
99	1	0	-0.246385	-3.839719	1.863304
100	1	0	3.297171	-0.960029	-0.098962
101	1	0	-0.291556	-1.519027	1.972156
102	1	0	0.579831	-1.121041	6.140698
103	1	0	1.946203	-1.864595	-2.273823
104	1	0	2.739656	-2.273469	5.824165
105	1	0	-0.972992	-2.879135	-1.896504
106	1	0	-0.930889	-0.777303	4.179386
107	1	0	5.992620	-4.483823	3.035092
108	1	0	2.117991	-4.263034	-5.821108
109	1	0	-1.645096	-7.881908	1.700502
110	1	0	2.541012	-2.116670	-4.617886
111	1	0	5.517961	-4.566475	-1.230389
112	1	0	6.848831	-5.122754	0.810178
113	1	0	-0.142627	7.103008	2.605383
114	1	0	4.330944	-3.389545	4.409292
115	1	0	0.142627	-7.103008	-2.605383
116	1	0	-4.330944	3.389545	-4.409292

No imaginary frequencies.
Zero-point correction= 0.936856 (Hartree/Particle)
Thermal correction to Energy= 0.988237
Thermal correction to Enthalpy= 0.989181
Thermal correction to Gibbs Free Energy= 0.849360
Sum of electronic and zero-point Energies= -2768.405900
Sum of electronic and thermal Energies= -2768.354519
Sum of electronic and thermal Enthalpies= -2768.353575
Sum of electronic and thermal Free Energies= -2768.493397

Table S3. Cartesian coordinate of **A-TH** TS-1 (ωB97X-D/6-31G**).

Center Number	Atomic Number	Atomic Type	Coordinates (Angstroms) X	Y	Z
1	6	0	-1.501194	-0.570570	-0.463590
2	6	0	-1.109849	3.617324	1.059671
3	6	0	-5.272800	2.420099	-1.547682
4	6	0	-1.822030	4.830080	0.854751
5	6	0	-4.208924	-0.604824	1.868242
6	6	0	-2.792483	-0.214398	-0.192585
7	6	0	-5.317498	-1.246319	2.514138
8	6	0	-5.485881	-2.819703	0.676361
9	6	0	-3.552614	0.449298	2.577834
10	6	0	-4.560224	1.186595	-1.388223
11	6	0	-4.763780	3.580796	-0.976926
12	6	0	-1.604616	2.424568	0.624767
13	6	0	-3.554003	3.572364	-0.293192
14	6	0	-2.846838	2.336329	-0.088447
15	6	0	-5.622753	-4.492765	-1.085300
16	6	0	-6.191741	0.133438	-2.873573
17	6	0	3.442862	1.175599	-0.155730
18	6	0	-4.377254	-2.187509	0.022998
19	6	0	-3.387036	1.126015	-0.594249
20	6	0	-5.053905	0.065856	-2.125653
21	6	0	-3.014565	4.800185	0.205357
22	6	0	-5.947498	-2.319890	1.890973
23	6	0	-0.631123	-3.571090	1.719622
24	6	0	-6.098576	-3.965218	0.074897
25	6	0	-0.693576	1.491889	-3.507004
26	6	0	-3.779198	-1.039457	0.595868
27	6	0	-3.988622	0.863027	3.800530
28	6	0	-3.896821	-2.796689	-1.185013
29	6	0	-6.938370	1.340242	-2.952128
30	6	0	-6.478888	2.452032	-2.315260
31	6	0	0.644083	1.804102	-3.287793
32	6	0	3.737439	-1.313220	0.157197
33	6	0	3.760438	-2.377065	-0.785878
34	6	0	-4.490153	-3.902624	-1.714678
35	6	0	-1.321730	-2.726598	0.869156
36	6	0	-5.754959	-0.775277	3.791871
37	6	0	4.798116	3.658995	-0.061617
38	6	0	-1.349309	0.691343	-2.591958
39	6	0	-0.720585	-1.617788	0.253519
40	6	0	0.674590	-1.423256	0.441089
41	6	0	6.252805	2.968128	-1.914984
42	6	0	4.749826	-3.410718	-0.667775
43	6	0	-0.713659	0.219397	-1.434945
44	6	0	2.788944	-0.171542	-0.075654
45	6	0	3.063350	2.160015	0.791419
46	6	0	1.429228	-0.330695	-0.239327
47	6	0	0.661162	0.465178	-1.241627
48	6	0	0.689914	-3.284473	2.032435
49	6	0	5.694177	-3.354253	0.353242
50	6	0	4.501833	1.447334	-1.054490
51	6	0	4.639489	-0.353025	2.281658
52	6	0	5.896432	0.826432	-2.955755
53	6	0	3.417645	4.384594	1.828279
54	6	0	-5.117287	0.252575	4.415993
55	6	0	4.882987	0.536226	-2.091087
56	6	0	5.682169	-2.329415	1.293914
57	6	0	5.186473	2.705158	-0.999477
58	6	0	1.312543	-2.220758	1.408832
59	6	0	2.862531	-2.467277	-1.901464
60	6	0	3.761661	3.414112	0.833541
61	6	0	3.880102	-4.541339	-2.637137
62	6	0	4.679176	-1.306848	1.214584
63	6	0	2.073621	1.933863	1.807182
64	6	0	4.771257	-4.486200	-1.611070
65	6	0	1.307484	1.269777	-2.198593
66	6	0	2.919302	-3.503780	-2.785281
67	6	0	6.641419	-2.299981	2.354456
68	6	0	2.480107	4.119569	2.777252
69	6	0	6.606904	2.054851	-2.860390
70	6	0	1.814035	2.861455	2.772032
71	6	0	5.557814	-0.366792	3.289052
72	6	0	6.592161	-1.342229	3.319982
73	1	0	-0.150220	3.642827	1.560138
74	1	0	-1.408649	5.766250	1.215656
75	1	0	-2.677537	0.909204	2.142133
76	1	0	-1.026774	1.527056	0.805526
77	1	0	-6.090678	-5.366561	-1.526660
78	1	0	-6.523108	-0.742581	-3.422110
79	1	0	-4.492448	-0.854227	-2.118943
80	1	0	-3.581114	5.711541	0.035556
81	1	0	-1.140973	-4.420627	2.161915
82	1	0	-6.950703	-4.412721	0.578758
83	1	0	-1.214332	1.851762	-4.388071
84	1	0	-3.465076	1.662674	4.314600
85	1	0	-3.023035	-2.373825	-1.669275
86	1	0	-7.851177	1.376380	-3.537867
87	1	0	-7.010989	3.396192	-2.391486
88	1	0	1.190555	2.424220	-3.990897
89	1	0	-4.092222	-4.343355	-2.623130
90	1	0	-2.357417	-2.944382	0.687148
91	1	0	-6.605291	-1.266167	4.256788
92	1	0	-2.379609	0.414366	-2.773694
93	1	0	6.769226	3.921067	-1.841911
94	1	0	1.237835	-3.879310	2.755637
95	1	0	3.848545	0.381227	2.307633
96	1	0	6.158249	0.115148	-3.732415
97	1	0	3.946987	5.333244	1.821077
98	1	0	-5.455130	0.600403	5.386890
99	1	0	4.341083	-0.392299	-2.205919
100	1	0	2.329598	-2.004664	1.690515
101	1	0	2.124587	-1.691969	-2.055600
102	1	0	3.904700	-5.361800	-3.346886
103	1	0	1.544438	0.989799	1.829747
104	1	0	5.524071	-5.259515	-1.486609
105	1	0	2.363056	1.464787	-2.115725
106	1	0	2.222473	-3.534359	-3.616729
107	1	0	7.405105	-3.072360	2.375867
108	1	0	2.243374	4.853308	3.540777
109	1	0	7.416056	2.264739	-3.552375
110	1	0	1.078838	2.641743	3.539964
111	1	0	5.491635	0.370148	4.083009
112	1	0	7.321963	-1.330952	4.123045
113	1	0	-6.800209	-2.792106	2.372686
114	1	0	6.450747	-4.132108	0.421878
115	1	0	5.324906	4.609163	-0.019151
116	1	0	-5.303769	4.517124	-1.094423

One imaginary frequency.
Zero-point correction= 0.937252 (Hartree/Particle)
Thermal correction to Energy= 0.987610
Thermal correction to Enthalpy= 0.988554
Thermal correction to Gibbs Free Energy= 0.854237
Sum of electronic and zero-point Energies= -2768.403099
Sum of electronic and thermal Energies= -2768.352741
Sum of electronic and thermal Enthalpies= -2768.351797
Sum of electronic and thermal Free Energies= -2768.486114

Table S4. Cartesian coordinate of **A-TH** TS-2 (ωB97X-D/6-31G**).

Center Number	Atomic Number	Atomic Type	Coordinates (Angstroms) X	Y	Z	Center Number	Atomic Number	Atomic Type	Coordinates (Angstroms) X	Y	Z
1	6	0	0.737187	1.213597	-1.703834	59	6	0	-5.926515	-1.421479	-3.039925
2	6	0	-0.670473	1.271313	-1.606270	60	6	0	-6.975532	-2.152444	-2.419008
3	6	0	-1.432582	0.032243	-1.274938	61	6	0	-6.809657	-2.625252	-1.152433
4	6	0	-0.737076	-1.213524	-1.703863	62	6	0	-3.417791	-1.239890	-0.268453
5	6	0	0.670593	-1.271232	-1.606453	63	6	0	-4.595486	-1.537589	-0.995922
6	6	0	1.432705	-0.032199	-1.275023	64	6	0	-5.603143	-2.382661	-0.424832
7	6	0	1.433495	2.332194	-2.170887	65	6	0	-5.371609	-2.983544	0.809418
8	6	0	0.777676	3.513042	-2.483056	66	6	0	-2.570229	-3.689253	3.106402
9	6	0	-0.601282	3.588806	-2.340740	67	6	0	-1.538831	-2.923519	2.496285
10	6	0	-1.311697	2.473689	-1.922743	68	6	0	-1.806078	-2.106284	1.438987
11	6	0	-1.433402	-2.332092	-2.170967	69	6	0	-3.114246	-2.032445	0.862205
12	6	0	-0.777582	-3.512886	-2.483341	70	6	0	-4.124738	-2.890293	1.420060
13	6	0	0.601398	-3.588623	-2.341223	71	6	0	-3.824894	-3.668981	2.581869
14	6	0	1.311827	-2.473533	-1.923176	72	6	0	-2.612847	0.004583	-0.609032
15	6	0	2.612921	-0.004580	-0.609033	73	1	0	2.504389	2.283403	-2.301359
16	6	0	3.341171	-2.632175	3.558153	74	1	0	1.345741	4.364868	-2.842051
17	6	0	3.713437	-2.364461	2.203351	75	1	0	-1.133253	4.503840	-2.579564
18	6	0	2.894180	-1.501523	1.398606	76	1	0	-2.386055	2.542796	-1.867164
19	6	0	1.690718	-1.009952	1.993586	77	1	0	-2.504310	-2.283322	-2.301329
20	6	0	1.355155	-1.304899	3.281228	78	1	0	-1.345669	-4.364692	-2.842349
21	6	0	2.200561	-2.114627	4.088899	79	1	0	1.133368	-4.503605	-2.580245
22	6	0	4.860446	-2.930538	1.658194	80	1	0	2.386198	-2.542590	-1.867760
23	6	0	5.181698	-2.744736	0.317232	81	1	0	3.989357	-3.272063	4.150099
24	6	0	4.368440	-1.886832	-0.497336	82	1	0	1.022390	-0.417308	1.386565
25	6	0	3.274302	-1.193344	0.071922	83	1	0	0.415833	-0.945844	3.686474
26	6	0	6.305662	-3.412664	-0.262530	84	1	0	1.921556	-2.328561	5.115468
27	6	0	6.584115	-3.302313	-1.590271	85	1	0	5.495879	-3.556922	2.279184
28	6	0	5.734397	-2.523663	-2.421984	86	1	0	6.921945	-4.030833	0.384089
29	6	0	4.679489	-1.842097	-1.892923	87	1	0	7.432515	-3.823718	-2.021223
30	6	0	6.975654	2.152562	-2.418895	88	1	0	5.923442	-2.477314	-3.489604
31	6	0	6.809706	2.625387	-1.152336	89	1	0	4.021121	-1.293387	-2.549991
32	6	0	5.603171	2.382764	-0.424781	90	1	0	7.887030	2.361843	-2.969423
33	6	0	4.595564	1.537646	-0.995899	91	1	0	7.579558	3.229722	-0.680837
34	6	0	4.791678	1.117047	-2.348373	92	1	0	3.977737	0.609410	-2.850254
35	6	0	5.926686	1.421557	-3.039845	93	1	0	6.026735	1.113785	-4.075749
36	6	0	5.371585	2.983646	0.809460	94	1	0	6.146707	3.596186	1.263041
37	6	0	4.124708	2.890337	1.420081	95	1	0	4.617205	4.276965	3.009299
38	6	0	3.114275	2.032443	0.862200	96	1	0	2.342684	4.308687	3.967956
39	6	0	3.417871	1.239897	-0.268450	97	1	0	0.521984	3.005406	2.863037
40	6	0	3.824808	3.668974	2.581910	98	1	0	0.992309	1.574095	0.964084
41	6	0	2.570142	3.689136	3.106447	99	1	0	-1.022527	0.417197	1.386743
42	6	0	1.538788	2.923371	2.496290	100	1	0	-0.416070	0.945874	3.686644
43	6	0	1.806089	2.106207	1.438951	101	1	0	-1.921844	2.328690	5.115487
44	6	0	-1.690882	1.009882	1.993696	102	1	0	-3.989637	3.272085	4.150001
45	6	0	-1.355372	1.304912	3.281334	103	1	0	-5.496116	3.556790	2.279021
46	6	0	-2.200814	2.114684	4.088923	104	1	0	-5.923195	2.477220	-3.489813
47	6	0	-3.341420	2.632175	3.558112	105	1	0	-4.020844	1.293418	-2.550070
48	6	0	-3.274314	1.193293	0.071913	106	1	0	-6.922150	4.030551	0.383839
49	6	0	-2.894305	1.501450	1.398632	107	1	0	-7.432519	3.823427	-2.021513
50	6	0	-3.713625	2.364393	2.203307	108	1	0	-3.977554	-0.609373	-2.850256
51	6	0	-4.860627	2.930418	1.658077	109	1	0	-6.026515	-1.113713	-4.075836
52	6	0	-5.734250	2.523538	-2.422175	110	1	0	-7.886888	-2.361709	-2.969574
53	6	0	-4.679339	1.842031	-1.893043	111	1	0	-7.579548	-3.229557	-0.680959
54	6	0	-4.368427	1.886750	-0.497424	112	1	0	-6.146769	-3.596042	1.262990
55	6	0	-5.181792	2.744594	0.317096	113	1	0	-2.342812	-4.308852	3.967887
56	6	0	-6.305766	3.412442	-0.262741	114	1	0	-0.522036	-3.005619	2.863042
57	6	0	-6.584104	3.302090	-1.590507	115	1	0	-0.992271	-1.574163	0.964181
58	6	0	-4.791529	-1.116991	-2.348408	116	1	0	-4.617339	-4.276917	3.009248

One imaginary frequency.
Zero-point correction= 0.937558 (Hartree/Particle)
Thermal correction to Energy= 0.987587
Thermal correction to Enthalpy= 0.988531
Thermal correction to Gibbs Free Energy= 0.856737
Sum of electronic and zero-point Energies= -2768.405676
Sum of electronic and thermal Energies= -2768.355648
Sum of electronic and thermal Enthalpies= -2768.354703
Sum of electronic and thermal Free Energies= -2768.486498

Table S5. Cartesian coordinate of **A-TH** twisted triplet state (UωB97X-D/6-31G**)

Center Number	Atomic Number	Atomic Type	Coordinates (Angstroms) X	Y	Z
1	6	0	-1.219629	-0.022734	-0.716349
2	6	0	-1.219629	0.022734	0.716349
3	6	0	-0.000000	0.000000	1.432832
4	6	0	1.219629	-0.022734	0.716349
5	6	0	1.219629	0.022734	-0.716349
6	6	0	-0.000000	-0.000000	-1.432832
7	6	0	-2.482370	-0.143070	-1.383982
8	6	0	-3.660425	-0.094108	-0.702953
9	6	0	-3.660425	0.094108	0.702953
10	6	0	-2.482370	0.143070	1.383982
11	6	0	2.482370	-0.143070	1.383982
12	6	0	3.660425	-0.094108	0.702953
13	6	0	3.660425	0.094108	-0.702953
14	6	0	2.482370	0.143070	-1.383982
15	6	0	0.000000	-0.000000	-2.919055
16	6	0	3.202725	-1.218622	-6.545417
17	6	0	2.396980	-1.601902	-5.429730
18	6	0	1.612794	-0.617209	-4.743901
19	6	0	1.769820	0.739393	-5.168112
20	6	0	2.562995	1.074216	-6.229023
21	6	0	3.277526	0.080961	-6.947152
22	6	0	2.400791	-2.922534	-4.982261
23	6	0	1.631704	-3.323428	-3.890529
24	6	0	0.794294	-2.372215	-3.220618
25	6	0	0.793583	-1.001671	-3.633847
26	6	0	1.644431	-4.686092	-3.461788
27	6	0	0.834340	-5.115287	-2.453436
28	6	0	-0.055880	-4.198570	-1.836952
29	6	0	-0.077922	-2.884131	-2.210218
30	6	0	-3.277526	-0.080961	-6.947152
31	6	0	-3.202725	1.218622	-6.545417
32	6	0	-2.396980	1.601902	-5.429730
33	6	0	-1.612794	0.617209	-4.743901
34	6	0	-1.769820	-0.739393	-5.168112
35	6	0	-2.562995	-1.074216	-6.229023
36	6	0	-2.400791	2.922534	-4.982261
37	6	0	-1.631704	3.323428	-3.890529
38	6	0	-0.794294	2.372215	-3.220618
39	6	0	-0.793583	1.001671	-3.633847
40	6	0	-1.644431	4.686092	-3.461788
41	6	0	-0.834340	5.115287	-2.453436
42	6	0	0.055880	4.198570	-1.836952
43	6	0	0.077922	2.884131	-2.210218
44	6	0	-1.769820	0.739393	5.168112
45	6	0	-2.562995	1.074216	6.229023
46	6	0	-3.277526	0.080961	6.947152
47	6	0	-3.202725	-1.218622	6.545417
48	6	0	-0.793583	-1.001671	3.633847
49	6	0	-1.612794	-0.617209	4.743901
50	6	0	-2.396980	-1.601902	5.429730
51	6	0	-2.400791	-2.922534	4.982261
52	6	0	0.055880	-4.198570	1.836952
53	6	0	0.077922	-2.884131	2.210218
54	6	0	-0.794294	-2.372215	3.220618
55	6	0	-1.631704	-3.323428	3.890529
56	6	0	-1.644431	-4.686092	3.461788
57	6	0	-0.834340	-5.115287	2.453436
58	6	0	1.769820	-0.739393	5.168112
59	6	0	2.562995	-1.074216	6.229023
60	6	0	3.277526	-0.080961	6.947152
61	6	0	3.202725	1.218622	6.545417
62	6	0	0.793583	1.001671	3.633847
63	6	0	1.612794	0.617209	4.743901
64	6	0	2.396980	1.601902	5.429730
65	6	0	2.400791	2.922534	4.982261
66	6	0	0.834340	5.115287	2.453436
67	6	0	-0.055880	4.198570	1.836952
68	6	0	-0.077922	2.884131	2.210218
69	6	0	0.794294	2.372215	3.220618
70	6	0	1.631704	3.323428	3.890529
71	6	0	1.644431	4.686092	3.461788
72	6	0	0.000000	0.000000	2.919055
73	1	0	-2.499697	-0.281635	-2.456087
74	1	0	-4.599303	-0.187391	-1.238875
75	1	0	-4.599303	0.187391	1.238875
76	1	0	-2.499697	0.281635	2.456087
77	1	0	2.499697	-0.281635	2.456087
78	1	0	4.599303	-0.187391	1.238875
79	1	0	4.599303	0.187391	-1.238875
80	1	0	2.499697	0.281635	-2.456087
81	1	0	3.765902	-1.992317	-7.059635
82	1	0	1.260337	1.528682	-4.634029
83	1	0	2.654547	2.116065	-6.518522
84	1	0	3.894704	0.362374	-7.794166
85	1	0	3.016927	-3.655952	-5.496537
86	1	0	2.309795	-5.376878	-3.972011
87	1	0	0.847986	-6.154044	-2.139160
88	1	0	-0.736859	-4.541932	-1.064703
89	1	0	-0.790090	-2.228432	-1.733139
90	1	0	-3.894704	-0.362374	-7.794166
91	1	0	-3.765902	1.992317	-7.059635
92	1	0	-1.260337	-1.528682	-4.634029
93	1	0	-2.654547	-2.116065	-6.518522
94	1	0	-3.016927	3.655952	-5.496537
95	1	0	-2.309795	5.376878	-3.972011
96	1	0	-0.847986	6.154044	-2.139160
97	1	0	0.736859	4.541932	-1.064703
98	1	0	0.790090	2.228432	-1.733139
99	1	0	-1.260337	1.528682	4.634029
100	1	0	-2.654547	2.116065	6.518522
101	1	0	-3.894704	0.362374	7.794166
102	1	0	-3.765902	-1.992317	7.059635
103	1	0	-3.016927	-3.655952	5.496537
104	1	0	0.736859	-4.541932	1.064703
105	1	0	0.790090	-2.228432	1.733139
106	1	0	-2.309795	-5.376878	3.972011
107	1	0	-0.847986	-6.154044	2.139160
108	1	0	1.260337	-1.528682	4.634029
109	1	0	2.654547	-2.116065	6.518522
110	1	0	3.894704	-0.362374	7.794166
111	1	0	3.765902	1.992317	7.059635
112	1	0	3.016927	3.655952	5.496537
113	1	0	0.847986	6.154044	2.139160
114	1	0	-0.736859	4.541932	1.064703
115	1	0	-0.790090	2.228432	1.733139
116	1	0	2.309795	5.376878	3.972011

No imaginary frequencies.
Zero-point correction= 0.933125 (Hartree/Particle)
Thermal correction to Energy= 0.984963
Thermal correction to Enthalpy= 0.985907
Thermal correction to Gibbs Free Energy= 0.846018
Sum of electronic and zero-point Energies= -2768.390783
Sum of electronic and thermal Energies= -2768.338946
Sum of electronic and thermal Enthalpies= -2768.338002
Sum of electronic and thermal Free Energies= -2768.477891

Table S6. Cartesian coordinate of **A-TH** radical cation state (UωB97X-D/6-31G**)

Center Number	Atomic Number	Atomic Type	Coordinates (Angstroms) X	Y	Z
1	6	0	-1.242349	-0.033989	-0.711546
2	6	0	-1.242349	0.033989	0.711546
3	6	0	0.000000	-0.000000	1.447532
4	6	0	1.242349	-0.033989	0.711546
5	6	0	1.242349	0.033989	-0.711546
6	6	0	-0.000000	-0.000000	-1.447532
7	6	0	-2.491045	-0.215643	-1.365971
8	6	0	-3.679218	-0.139927	-0.688644
9	6	0	-3.679218	0.139927	0.688644
10	6	0	-2.491045	0.215643	1.365971
11	6	0	2.491045	-0.215643	1.365971
12	6	0	3.679218	-0.139927	0.688644
13	6	0	3.679218	0.139927	-0.688644
14	6	0	2.491045	0.215643	-1.365971
15	6	0	-0.000000	0.000000	-2.881582
16	6	0	3.331429	-0.664036	-6.545902
17	6	0	2.581800	-1.175579	-5.443075
18	6	0	1.687955	-0.317866	-4.723050
19	6	0	1.670379	1.059548	-5.102261
20	6	0	2.411731	1.518892	-6.154260
21	6	0	3.242407	0.646832	-6.904475
22	6	0	2.741950	-2.500003	-5.039730
23	6	0	2.038131	-3.034225	-3.961820
24	6	0	1.110150	-2.211986	-3.243098
25	6	0	0.948995	-0.841179	-3.618412
26	6	0	2.212638	-4.404850	-3.598711
27	6	0	1.470907	-4.967274	-2.604648
28	6	0	0.495389	-4.181419	-1.938454
29	6	0	0.319650	-2.861229	-2.243668
30	6	0	-3.242407	-0.646832	-6.904475
31	6	0	-3.331429	0.664036	-6.545902
32	6	0	-2.581800	1.175579	-5.443075
33	6	0	-1.687955	0.317866	-4.723050
34	6	0	-1.670379	-1.059548	-5.102261
35	6	0	-2.411731	-1.518892	-6.154260
36	6	0	-2.741950	2.500003	-5.039730
37	6	0	-2.038131	3.034225	-3.961820
38	6	0	-1.110150	2.211986	-3.243098
39	6	0	-0.948995	0.841179	-3.618412
40	6	0	-2.212638	4.404850	-3.598711
41	6	0	-1.470907	4.967274	-2.604648
42	6	0	-0.495389	4.181419	-1.938454
43	6	0	-0.319650	2.861229	-2.243668
44	6	0	-1.670379	1.059548	5.102261
45	6	0	-2.411731	1.518892	6.154260
46	6	0	-3.242407	0.646832	6.904475
47	6	0	-3.331429	-0.664036	6.545902
48	6	0	-0.948995	-0.841179	3.618412
49	6	0	-1.687955	-0.317866	4.723050
50	6	0	-2.581800	-1.175579	5.443075
51	6	0	-2.741950	-2.500003	5.039730
52	6	0	-0.495389	-4.181419	1.938454
53	6	0	-0.319650	-2.861229	2.243668
54	6	0	-1.110150	-2.211986	3.243098
55	6	0	-2.038131	-3.034225	3.961820
56	6	0	-2.212638	-4.404850	3.598711
57	6	0	-1.470907	-4.967274	2.604648
58	6	0	1.670379	-1.059548	5.102261
59	6	0	2.411731	-1.518892	6.154260
60	6	0	3.242407	-0.646832	6.904475
61	6	0	3.331429	0.664036	6.545902
62	6	0	0.948995	0.841179	3.618412
63	6	0	1.687955	0.317866	4.723050
64	6	0	2.581800	1.175579	5.443075
65	6	0	2.741950	2.500003	5.039730
66	6	0	1.470907	4.967274	2.604648
67	6	0	0.495389	4.181419	1.938454
68	6	0	0.319650	2.861229	2.243668
69	6	0	1.110150	2.211986	3.243098
70	6	0	2.038131	3.034225	3.961820
71	6	0	2.212638	4.404850	3.598711
72	6	0	0.000000	0.000000	2.881582
73	1	0	-2.515282	-0.417423	-2.425854
74	1	0	-4.613606	-0.276293	-1.221801
75	1	0	-4.613606	0.276293	1.221801
76	1	0	-2.515282	0.417423	2.425854
77	1	0	2.515282	-0.417423	2.425854
78	1	0	4.613606	-0.276293	1.221801
79	1	0	4.613606	0.276293	-1.221801
80	1	0	2.515282	0.417423	-2.425854
81	1	0	3.983998	-1.344789	-7.084099
82	1	0	1.069442	1.764516	-4.545757
83	1	0	2.370686	2.571481	-6.414125
84	1	0	3.815108	1.031270	-7.741196
85	1	0	3.434267	-3.136786	-5.584696
86	1	0	2.941094	-4.992738	-4.148869
87	1	0	1.600800	-6.010993	-2.340475
88	1	0	-0.140570	-4.638639	-1.187218
89	1	0	-0.457165	-2.316070	-1.726361
90	1	0	-3.815108	-1.031270	-7.741196
91	1	0	-3.983998	1.344789	-7.084099
92	1	0	-1.069442	-1.764516	-4.545757
93	1	0	-2.370686	-2.571481	-6.414125
94	1	0	-3.434267	3.136786	-5.584696
95	1	0	-2.941094	4.992738	-4.148869
96	1	0	-1.600800	6.010993	-2.340475
97	1	0	0.140570	4.638639	-1.187218
98	1	0	0.457165	2.316070	-1.726361
99	1	0	-1.069442	1.764516	4.545757
100	1	0	-2.370686	2.571481	6.414125
101	1	0	-3.815108	1.031270	7.741196
102	1	0	-3.983998	-1.344789	7.084099
103	1	0	-3.434267	-3.136786	5.584696
104	1	0	0.140570	-4.638639	1.187218
105	1	0	0.457165	-2.316070	1.726361
106	1	0	-2.941094	-4.992738	4.148869
107	1	0	-1.600800	-6.010993	2.340475
108	1	0	1.069442	-1.764516	4.545757
109	1	0	2.370686	-2.571481	6.414125
110	1	0	3.815108	-1.031270	7.741196
111	1	0	3.983998	1.344789	7.084099
112	1	0	3.434267	3.136786	5.584696
113	1	0	1.600800	6.010993	2.340475
114	1	0	-0.140570	4.638639	1.187218
115	1	0	-0.457165	2.316070	1.726361
116	1	0	2.941094	4.992738	4.148869

No imaginary frequencies.
Zero-point correction= 0.936450 (Hartree/Particle)
Thermal correction to Energy= 0.988162
Thermal correction to Enthalpy= 0.989106
Thermal correction to Gibbs Free Energy= 0.849051
Sum of electronic and zero-point Energies= -2768.197326
Sum of electronic and thermal Energies= -2768.145614
Sum of electronic and thermal Enthalpies= -2768.144670
Sum of electronic and thermal Free Energies= -2768.284725

Table S7. Cartesian coordinate of **A-TH** dication state (ωB97X-D/6-31G**)

Center Number	Atomic Number	Atomic Type	Coordinates (Angstroms) X	Y	Z
1	6	0	-1.228384	-0.008422	-0.716946
2	6	0	-1.228384	0.008422	0.716946
3	6	0	0.000000	0.000000	1.423628
4	6	0	1.228384	-0.008422	0.716946
5	6	0	1.228384	0.008422	-0.716946
6	6	0	-0.000000	0.000000	-1.423628
7	6	0	-2.491676	-0.113393	-1.385883
8	6	0	-3.669938	-0.078731	-0.704229
9	6	0	-3.669938	0.078731	0.704229
10	6	0	-2.491676	0.113393	1.385883
11	6	0	2.491676	-0.113393	1.385883
12	6	0	3.669938	-0.078731	0.704229
13	6	0	3.669938	0.078731	-0.704229
14	6	0	2.491676	0.113393	-1.385883
15	6	0	-0.000000	-0.000000	-2.914442
16	6	0	3.398686	-1.316783	-6.305734
17	6	0	2.520372	-1.631861	-5.226728
18	6	0	1.596757	-0.655759	-4.722987
19	6	0	1.668975	0.635822	-5.308543
20	6	0	2.528834	0.909253	-6.343190
21	6	0	3.397378	-0.074549	-6.867817
22	6	0	2.584921	-2.897384	-4.645547
23	6	0	1.741373	-3.292150	-3.606242
24	6	0	0.789362	-2.361205	-3.089806
25	6	0	0.755631	-1.011217	-3.597006
26	6	0	1.787504	-4.629523	-3.117574
27	6	0	0.882709	-5.062068	-2.190462
28	6	0	-0.119206	-4.171552	-1.740725
29	6	0	-0.169151	-2.870922	-2.175220
30	6	0	-3.397378	0.074549	-6.867817
31	6	0	-3.398686	1.316783	-6.305734
32	6	0	-2.520372	1.631861	-5.226728
33	6	0	-1.596757	0.655759	-4.722987
34	6	0	-1.668975	-0.635822	-5.308543
35	6	0	-2.528834	-0.909253	-6.343190
36	6	0	-2.584921	2.897384	-4.645547
37	6	0	-1.741373	3.292150	-3.606242
38	6	0	-0.789362	2.361205	-3.089806
39	6	0	-0.755631	1.011217	-3.597006
40	6	0	-1.787504	4.629523	-3.117574
41	6	0	-0.882709	5.062068	-2.190462
42	6	0	0.119206	4.171552	-1.740725
43	6	0	0.169151	2.870922	-2.175220
44	6	0	-1.668975	0.635822	5.308543
45	6	0	-2.528834	0.909253	6.343190
46	6	0	-3.397378	-0.074549	6.867817
47	6	0	-3.398686	-1.316783	6.305734
48	6	0	-0.755631	-1.011217	3.597006
49	6	0	-1.596757	-0.655759	4.722987
50	6	0	-2.520372	-1.631861	5.226728
51	6	0	-2.584921	-2.897384	4.645547
52	6	0	0.119206	-4.171552	1.740725
53	6	0	0.169151	-2.870922	2.175220
54	6	0	-0.789362	-2.361205	3.089806
55	6	0	-1.741373	-3.292150	3.606242
56	6	0	-1.787504	-4.629523	3.117574
57	6	0	-0.882709	-5.062068	2.190462
58	6	0	1.668975	-0.635822	5.308543
59	6	0	2.528834	-0.909253	6.343190
60	6	0	3.397378	0.074549	6.867817
61	6	0	3.398686	1.316783	6.305734
62	6	0	0.755631	1.011217	3.597006
63	6	0	1.596757	0.655759	4.722987
64	6	0	2.520372	1.631861	5.226728
65	6	0	2.584921	2.897384	4.645547
66	6	0	0.882709	5.062068	2.190462
67	6	0	-0.119206	4.171552	1.740725
68	6	0	-0.169151	2.870922	2.175220
69	6	0	0.789362	2.361205	3.089806
70	6	0	1.741373	3.292150	3.606242
71	6	0	1.787504	4.629523	3.117574
72	6	0	0.000000	-0.000000	2.914442
73	1	0	-2.517584	-0.231047	-2.459921
74	1	0	-4.608815	-0.160845	-1.240521
75	1	0	-4.608815	0.160845	1.240521
76	1	0	-2.517584	0.231047	2.459921
77	1	0	2.517584	-0.231047	2.459921
78	1	0	4.608815	-0.160845	1.240521
79	1	0	4.608815	0.160845	-1.240521
80	1	0	2.517584	0.231047	-2.459921
81	1	0	4.073614	-2.088183	-6.662989
82	1	0	1.038537	1.435302	-4.949572
83	1	0	2.542021	1.908952	-6.764516
84	1	0	4.060821	0.165672	-7.690332
85	1	0	3.303347	-3.613981	-5.036567
86	1	0	2.536246	-5.303959	-3.521000
87	1	0	0.903959	-6.085509	-1.833191
88	1	0	-0.879069	-4.524665	-1.051042
89	1	0	-0.983901	-2.248362	-1.836087
90	1	0	-4.060821	-0.165672	-7.690332
91	1	0	-4.073614	2.088183	-6.662989
92	1	0	-1.038537	-1.435302	-4.949572
93	1	0	-2.542021	-1.908952	-6.764516
94	1	0	-3.303347	3.613981	-5.036567
95	1	0	-2.536246	5.303959	-3.521000
96	1	0	-0.903959	6.085509	-1.833191
97	1	0	0.879069	4.524665	-1.051042
98	1	0	0.983901	2.248362	-1.836087
99	1	0	-1.038537	1.435302	4.949572
100	1	0	-2.542021	1.908952	6.764516
101	1	0	-4.060821	0.165672	7.690332
102	1	0	-4.073614	-2.088183	6.662989
103	1	0	-3.303347	-3.613981	5.036567
104	1	0	0.879069	-4.524665	1.051042
105	1	0	0.983901	-2.248362	1.836087
106	1	0	-2.536246	-5.303959	3.521000
107	1	0	-0.903959	-6.085509	1.833191
108	1	0	1.038537	-1.435302	4.949572
109	1	0	2.542021	-1.908952	6.764516
110	1	0	4.060821	-0.165672	7.690332
111	1	0	4.073614	2.088183	6.662989
112	1	0	3.303347	3.613981	5.036567
113	1	0	0.903959	6.085509	1.833191
114	1	0	-0.879069	4.524665	1.051042
115	1	0	-0.983901	2.248362	1.836087
116	1	0	2.536246	5.303959	3.521000

No imaginary frequencies.
Zero-point correction= 0.938548 (Hartree/Particle)
Thermal correction to Energy= 0.990230
Thermal correction to Enthalpy= 0.991175
Thermal correction to Gibbs Free Energy= 0.851423
Sum of electronic and zero-point Energies= -2767.910376
Sum of electronic and thermal Energies= -2767.858693
Sum of electronic and thermal Enthalpies= -2767.857749
Sum of electronic and thermal Free Energies= -2767.997501

Table S8. Cartesian coordinate of **A-CH** folded (ωB97X-D/6-31G**)

Center Number	Atomic Number	Atomic Type	Coordinates (Angstroms) X	Y	Z	Center Number	Atomic Number	Atomic Type	Coordinates (Angstroms) X	Y	Z
1	6	0	2.529371	1.672097	0.546530	70	6	0	-4.036080	3.589040	1.124714
2	6	0	1.133606	1.574457	0.323167	71	6	0	-3.150234	4.311206	1.987217
3	6	0	0.482473	0.232914	0.414347	72	6	0	-4.415312	0.007405	-0.193953
4	6	0	1.058284	-0.559048	1.524239	73	6	0	-3.124694	-2.936549	-0.512004
5	6	0	2.455751	-0.552163	1.621039	74	6	0	-2.370086	-4.079764	-0.281328
6	6	0	3.240238	0.374240	0.759986	75	6	0	-0.999317	-3.982725	-0.078062
7	6	0	3.125035	2.936602	0.512229	76	6	0	-0.390894	-2.736860	-0.098820
8	6	0	2.370429	4.079839	0.281623	77	6	0	-0.295907	1.249711	-2.467936
9	6	0	0.999671	3.982817	0.078319	78	6	0	-0.919108	1.977174	-3.472113
10	6	0	0.391240	2.736953	0.098999	79	6	0	-2.310613	2.012993	-3.544300
11	6	0	0.296313	-1.249558	2.468149	80	6	0	-3.072643	1.293769	-2.633966
12	6	0	0.919609	-1.977086	3.472226	81	6	0	-3.239957	-0.374255	-0.760116
13	6	0	2.311121	-2.013027	3.544205	82	6	0	-2.529027	-1.672043	-0.546455
14	6	0	3.073073	-1.293858	2.633766	83	6	0	-1.133281	-1.574380	-0.323069
15	6	0	4.415547	-0.007475	0.193734	84	6	0	-0.482191	-0.232814	-0.414206
16	6	0	7.128819	-4.202679	0.873471	85	6	0	-1.057957	0.559130	-1.524133
17	6	0	6.011897	-3.534355	0.280445	86	6	0	-2.455414	0.552148	-1.621127
18	6	0	5.785247	-2.144833	0.560417	87	1	0	4.183548	3.042917	0.689987
19	6	0	6.688036	-1.509700	1.471996	88	1	0	2.860790	5.048083	0.274459
20	6	0	7.736506	-2.181262	2.027093	89	1	0	0.398213	4.871011	-0.085143
21	6	0	7.972008	-3.549609	1.718761	90	1	0	-0.677613	2.663648	-0.050205
22	6	0	5.133966	-4.221731	-0.552107	91	1	0	-0.786472	-1.227100	2.402183
23	6	0	4.035044	-3.588991	-1.124731	92	1	0	0.320590	-2.512875	4.201362
24	6	0	3.795589	-2.199066	-0.857745	93	1	0	2.802892	-2.581166	4.327097
25	6	0	4.679555	-1.478022	-0.012165	94	1	0	4.154765	-1.288748	2.717837
26	6	0	3.148934	-4.310823	-1.987242	95	1	0	7.282981	-5.251574	0.635991
27	6	0	2.092793	-3.699213	-2.588410	96	1	0	6.532726	-0.474744	1.736580
28	6	0	1.860470	-2.315974	-2.353890	97	1	0	8.398210	-1.665041	2.715363
29	6	0	2.666837	-1.605052	-1.517045	98	1	0	8.814837	-4.066937	2.165549
30	6	0	6.597610	1.292025	-4.377243	99	1	0	5.310427	-5.274434	-0.759529
31	6	0	7.280545	1.985639	-3.426481	100	1	0	3.348500	-5.365465	-2.155610
32	6	0	6.916571	1.898905	-2.045946	101	1	0	1.427398	-4.255595	-3.239931
33	6	0	5.823849	1.055574	-1.650760	102	1	0	1.012645	-1.823947	-2.820524
34	6	0	5.127742	0.361829	-2.690441	103	1	0	2.442347	-0.561108	-1.346281
35	6	0	5.498493	0.472957	-3.997061	104	1	0	6.880206	1.365602	-5.422398
36	6	0	7.606338	2.628798	-1.083071	105	1	0	8.115553	2.625680	-3.697223
37	6	0	7.246124	2.575495	0.259902	106	1	0	4.280987	-0.257131	-2.438504
38	6	0	6.155873	1.737594	0.670442	107	1	0	4.944536	-0.067787	-4.757724
39	6	0	5.458043	0.960441	-0.289552	108	1	0	8.437707	3.260338	-1.386825
40	6	0	7.951703	3.351001	1.234143	109	1	0	8.774149	3.973504	0.893095
41	6	0	7.605140	3.316894	2.549026	110	1	0	8.145727	3.910971	3.278468
42	6	0	6.520279	2.496439	2.967066	111	1	0	6.237908	2.475712	4.014783
43	6	0	5.827690	1.740205	2.069477	112	1	0	4.996651	1.137359	2.414780
44	6	0	-5.827483	-1.740670	-2.069324	113	1	0	-4.996189	-1.138178	-2.414657
45	6	0	-6.520242	-2.496883	-2.966798	114	1	0	-6.237742	-2.476544	-4.014487
46	6	0	-7.605436	-3.316846	-2.548649	115	1	0	-8.146188	-3.910891	-3.277995
47	6	0	-7.952104	-3.350554	-1.233780	116	1	0	-8.774783	-3.972713	-0.892667
48	6	0	-5.457780	-0.960491	0.289515	117	1	0	-8.438137	-3.259280	1.387185
49	6	0	-6.155799	-1.737627	-0.670329	118	1	0	-4.942990	0.067331	4.757607
50	6	0	-7.246301	-2.575120	-0.259647	119	1	0	-4.279674	0.256308	2.438243
51	6	0	-7.606521	-2.628108	1.083344	120	1	0	-8.115460	-2.624624	3.697585
52	6	0	-5.497350	-0.473098	3.997015	121	1	0	-6.879307	-1.365057	5.422551
53	6	0	-5.126759	-0.362145	2.690341	122	1	0	-6.531927	0.474296	-1.737562
54	6	0	-5.823391	-1.055529	1.650771	123	1	0	-8.397893	1.663910	-2.716283
55	6	0	-6.916390	-1.898421	2.046117	124	1	0	-8.815883	4.065411	-2.165748
56	6	0	-7.280217	-1.984946	3.426706	125	1	0	-7.284787	5.250411	-0.635727
57	6	0	-6.596830	-1.291622	4.377354	126	1	0	-5.312338	5.273911	0.759940
58	6	0	-6.687880	1.509065	-1.472625	127	1	0	-1.428491	4.256731	3.239644
59	6	0	-7.736601	2.180252	-2.027710	128	1	0	-1.012566	1.825319	2.819879
60	6	0	-7.972859	3.548375	-1.718990	129	1	0	-2.441893	0.561915	1.345836
61	6	0	-7.130091	4.201644	-0.873429	130	1	0	-3.350334	5.365720	2.155757
62	6	0	-4.679702	1.477899	0.011931	131	1	0	-4.183216	-3.042867	-0.689699
63	6	0	-5.785602	2.144366	-0.560671	132	1	0	-2.860434	-5.048015	-0.274087
64	6	0	-6.012915	3.533721	-0.280441	133	1	0	-0.397857	-4.870909	0.085438
65	6	0	-5.135343	4.221343	0.552286	134	1	0	0.677958	-2.663538	0.050395
66	6	0	-2.093675	3.700083	2.588134	135	1	0	0.786871	1.227305	-2.401817
67	6	0	-1.860686	2.316988	2.353407	136	1	0	-0.320034	2.512987	-4.201185
68	6	0	-2.666854	1.605742	1.516644	137	1	0	-2.802312	2.581091	-4.327267
69	6	0	-3.795986	2.199246	0.857528	138	1	0	-4.154318	1.288570	-2.718223

No imaginary frequencies.

Zero-point correction= 1.113726 (Hartree/Particle)

Thermal correction to Energy= 1.174907

Thermal correction to Enthalpy= 1.175851

Thermal correction to Gibbs Free Energy= 1.016979

Sum of electronic and zero-point Energies= -3306.406054

Sum of electronic and thermal Energies= -3306.344874

Sum of electronic and thermal Enthalpies= -3306.343930

Sum of electronic and thermal Free Energies= -3306.502802

Table S9. Cartesian coordinate of **A-CH** twisted singlet (y_0 = 0.985, UωB97X-D/6-31G**)

Center Number	Atomic Number	Atomic Type	Coordinates (Angstroms) X	Y	Z
1	6	0	2.972783	-1.425674	0.630086
2	6	0	1.542513	-1.539441	0.663223
3	6	0	0.741094	-0.650279	-0.074124
4	6	0	1.333672	0.297294	-0.927287
5	6	0	2.757930	0.403342	-0.992081
6	6	0	3.581539	-0.400396	-0.152358
7	6	0	3.715487	-2.285322	1.499974
8	6	0	3.103153	-3.221827	2.281519
9	6	0	1.691984	-3.358746	2.274917
10	6	0	0.938124	-2.531325	1.499085
11	6	0	0.532257	1.166119	-1.734507
12	6	0	1.104192	2.046629	-2.602632
13	6	0	2.515874	2.094845	-2.734489
14	6	0	3.310069	1.301389	-1.958338
15	6	0	5.010835	-0.081793	-0.024929
16	6	0	7.822391	3.906382	-1.115317
17	6	0	6.759069	3.311026	-0.368958
18	6	0	6.440333	1.926318	-0.562368
19	6	0	7.163538	1.235160	-1.585636
20	6	0	8.163914	1.839954	-2.291575
21	6	0	8.517343	3.192270	-2.043676
22	6	0	6.003700	4.081099	0.514042
23	6	0	4.960923	3.526303	1.253226
24	6	0	4.665267	2.129653	1.127556
25	6	0	5.383703	1.327460	0.188377
26	6	0	4.204388	4.334659	2.157760
27	6	0	3.235165	3.792660	2.946124
28	6	0	2.997317	2.393523	2.895787
29	6	0	3.689593	1.595938	2.029524
30	6	0	9.205760	-1.338442	2.763978
31	6	0	9.160861	-2.224018	1.729878
32	6	0	8.099233	-2.188692	0.774257
33	6	0	7.087073	-1.178560	0.872993
34	6	0	7.147637	-0.308465	2.006070
35	6	0	8.168579	-0.380164	2.910320
36	6	0	8.022105	-3.151242	-0.231582
37	6	0	6.987140	-3.147348	-1.166181
38	6	0	5.989223	-2.119187	-1.120062
39	6	0	6.032423	-1.129191	-0.089790
40	6	0	6.934201	-4.141860	-2.190633
41	6	0	5.971507	-4.109443	-3.153964
42	6	0	5.018744	-3.057435	-3.154725
43	6	0	5.032003	-2.098333	-2.182299
44	6	0	-7.163540	1.235285	1.585427
45	6	0	-8.163947	1.840142	2.291265
46	6	0	-8.517279	3.192475	2.043315
47	6	0	-7.822202	3.906539	1.115014
48	6	0	-5.383535	1.327470	-0.188424
49	6	0	-6.440191	1.926395	0.562219
50	6	0	-6.758840	3.311120	0.368763
51	6	0	-6.003348	4.081132	-0.514182
52	6	0	-2.996862	2.393357	-2.895653
53	6	0	-3.689256	1.595823	-2.029441
54	6	0	-4.664983	2.129593	-1.127554
55	6	0	-4.960542	3.526265	-1.253263
56	6	0	-4.203875	4.334570	-2.157740
57	6	0	-3.234621	3.792512	-2.946020
58	6	0	-7.147251	-0.308401	-2.006429
59	6	0	-8.168081	-0.379986	-2.910819
60	6	0	-9.205456	-1.338055	-2.764541
61	6	0	-9.160866	-2.223557	-1.730361
62	6	0	-6.032493	-1.129128	0.089665
63	6	0	-7.087011	-1.178407	-0.873272
64	6	0	-8.099367	-2.188349	-0.774596
65	6	0	-8.022546	-3.150834	0.231327
66	6	0	-5.972451	-4.109294	3.153970
67	6	0	-5.019471	-3.057487	3.154770
68	6	0	-5.032437	-2.098421	2.182302
69	6	0	-5.989559	-2.119110	1.119978
70	6	0	-6.987675	-3.147083	1.166035
71	6	0	-6.935049	-4.141557	2.190536
72	6	0	-5.010806	-0.081845	0.024930
73	6	0	-3.715381	-2.285443	-1.499770
74	6	0	-3.102976	-3.221868	-2.281360
75	6	0	-1.691799	-3.358683	-2.274760
76	6	0	-0.937999	-2.531266	-1.498866
77	6	0	-0.532426	1.165881	1.735106
78	6	0	-1.104449	2.046315	2.603252
79	6	0	-2.516140	2.094539	2.734969
80	6	0	-3.310264	1.301145	1.958676
81	6	0	-3.581558	-0.400515	0.152573
82	6	0	-2.972739	-1.425781	-0.629851
83	6	0	-1.542456	-1.539472	-0.662946
84	6	0	-0.741110	-0.650330	0.074496
85	6	0	-1.333760	0.297133	0.927732
86	6	0	-2.758024	0.403163	0.992423
87	1	0	4.790727	-2.195318	1.545837
88	1	0	3.704071	-3.860241	2.921106
89	1	0	1.215948	-4.108272	2.898705
90	1	0	-0.143202	-2.610605	1.503678
91	1	0	-0.547430	1.110928	-1.649611
92	1	0	0.479342	2.697770	-3.205933
93	1	0	2.968472	2.760575	-3.462217
94	1	0	4.382887	1.338088	-2.093712
95	1	0	8.055557	4.951066	-0.929549
96	1	0	6.911923	0.210286	-1.818376
97	1	0	8.689456	1.280286	-3.058579
98	1	0	9.321709	3.654300	-2.606769
99	1	0	6.233845	5.137485	0.628591
100	1	0	4.430767	5.396028	2.206867
101	1	0	2.666918	4.415518	3.629334
102	1	0	2.268789	1.946048	3.565369
103	1	0	3.505008	0.531765	2.047808
104	1	0	10.014672	-1.375205	3.486427
105	1	0	9.927411	-2.985387	1.616537
106	1	0	6.362232	0.417165	2.163323
107	1	0	8.180063	0.297142	3.758098
108	1	0	8.785095	-3.923754	-0.287206
109	1	0	7.690565	-4.921667	-2.187110
110	1	0	5.941266	-4.868285	-3.929093
111	1	0	4.275835	-3.007097	-3.944222
112	1	0	4.303708	-1.301513	-2.229381
113	1	0	-6.912006	0.210399	1.818205
114	1	0	-8.689586	1.280515	3.058232
115	1	0	-9.321676	3.654558	2.606319
116	1	0	-8.055301	4.951232	0.929213
117	1	0	-6.233431	5.137527	-0.628783
118	1	0	-2.268298	1.945820	-3.565158
119	1	0	-3.504717	0.531644	-2.047694
120	1	0	-4.430176	5.395954	-2.206859
121	1	0	-2.666281	4.415335	-3.629185
122	1	0	-6.361683	0.417063	-2.163631
123	1	0	-8.179314	0.297250	-3.758658
124	1	0	-10.014274	-1.374735	-3.487101
125	1	0	-9.927570	-2.984779	-1.617076
126	1	0	-8.785696	-3.923191	0.286919
127	1	0	-5.942450	-4.868102	3.929142
128	1	0	-4.276619	-3.007276	3.944330
129	1	0	-4.303965	-1.301768	2.229422
130	1	0	-7.691570	-4.921211	2.186965
131	1	0	-4.790629	-2.195517	-1.545625
132	1	0	-3.703850	-3.860298	-2.920972
133	1	0	-1.215706	-4.108131	-2.898598
134	1	0	0.143331	-2.610487	-1.503449
135	1	0	0.547270	1.110689	1.650311
136	1	0	-0.479660	2.697402	3.206674
137	1	0	-2.968802	2.760217	3.462705
138	1	0	-4.383097	1.337840	2.093931

No imaginary frequencies.

Zero-point correction= 1.108879 (Hartree/Particle)

Thermal correction to Energy= 1.171027

Thermal correction to Enthalpy= 1.171971

Thermal correction to Gibbs Free Energy= 1.007106

Sum of electronic and zero-point Energies= -3306.382454

Sum of electronic and thermal Energies= -3306.320306

Sum of electronic and thermal Enthalpies= -3306.319362

Sum of electronic and thermal Free Energies= -3306.484226

Table S10. Cartesian coordinate of **A-CH** twisted triplet (UωB97X-D/6-31G**)

Center Number	Atomic Number	Atomic Type	X	Y	Z	Center Number	Atomic Number	Atomic Type	X	Y	Z
			Coordinates (Angstroms)						Coordinates (Angstroms)		
1	6	0	0.641691	2.970913	-1.411416	70	6	0	1.152759	-6.971566	-3.163663
2	6	0	0.673866	1.540454	-1.523590	71	6	0	2.169686	-6.910745	-4.165391
3	6	0	-0.072810	0.741437	-0.640844	72	6	0	0.026747	-5.013170	-0.080964
4	6	0	-0.932234	1.335552	0.299316	73	6	0	-1.520431	-3.711740	-2.263975
5	6	0	-0.996221	2.760295	0.403293	74	6	0	-2.309230	-3.097221	-3.192757
6	6	0	-0.150087	3.581490	-0.395244	75	6	0	-2.300821	-1.685816	-3.329334
7	6	0	1.520431	3.711740	-2.263975	76	6	0	-1.516287	-0.933788	-2.508649
8	6	0	2.309230	3.097221	-3.192757	77	6	0	1.745172	-0.535741	1.164575
9	6	0	2.300821	1.685816	-3.329334	78	6	0	2.618667	-1.109561	2.038294
10	6	0	1.516287	0.933788	-2.508649	79	6	0	2.749916	-2.521607	2.083583
11	6	0	-1.745172	0.535741	1.164575	80	6	0	1.967935	-3.314312	1.294674
12	6	0	-2.618667	1.109561	2.038294	81	6	0	0.150087	-3.581490	-0.395244
13	6	0	-2.749916	2.521607	2.083583	82	6	0	-0.641691	-2.970913	-1.411416
14	6	0	-1.967935	3.314312	1.294674	83	6	0	-0.673866	-1.540454	-1.523590
15	6	0	-0.026747	5.013170	-0.080964	84	6	0	0.072810	-0.741437	-0.640844
16	6	0	-1.146481	7.834491	3.891790	85	6	0	0.932234	-1.335552	0.299316
17	6	0	-0.394520	6.770882	3.304074	86	6	0	0.996221	-2.760295	0.403293
18	6	0	-0.578118	6.448405	1.918880	87	1	0	1.567193	4.787021	-2.174328
19	6	0	-1.597783	7.168398	1.219144	88	1	0	2.955652	3.696425	-3.825888
20	6	0	-2.309230	8.169254	1.816714	89	1	0	2.929813	1.208439	-4.073637
21	6	0	-2.071017	8.526360	3.169761	90	1	0	1.518371	-0.147576	-2.587949
22	6	0	0.484435	6.018845	4.082044	91	1	0	-1.659427	-0.544051	1.112164
23	6	0	1.229200	4.975968	3.534902	92	1	0	-3.226419	0.486396	2.686879
24	6	0	1.113432	4.676598	2.138238	93	1	0	-3.481569	2.975496	2.744121
25	6	0	0.178292	5.391177	1.327823	94	1	0	-2.102421	4.387275	1.329758
26	6	0	2.129788	4.223208	4.351096	95	1	0	-0.968107	8.070448	4.937138
27	6	0	2.923732	3.254289	3.816681	96	1	0	-1.823299	6.913994	0.193333
28	6	0	2.883297	3.013057	2.417829	97	1	0	-3.073198	8.692280	1.250564
29	6	0	2.020988	3.701703	1.612837	98	1	0	-2.638394	9.331007	3.626022
30	6	0	2.757340	9.211268	-1.337144	99	1	0	0.591413	6.251845	5.138596
31	6	0	1.729804	9.158688	-2.229942	100	1	0	2.171392	4.452238	5.412217
32	6	0	0.777281	8.094248	-2.196282	101	1	0	3.603855	2.688886	4.445465
33	6	0	0.871705	7.087385	-1.180468	102	1	0	3.557566	2.285104	1.976519
34	6	0	1.998397	7.155744	-0.302762	103	1	0	2.046954	3.514952	0.549220
35	6	0	2.899996	8.179192	-0.372909	104	1	0	3.477466	10.022302	-1.372637
36	6	0	-0.221154	8.009263	-3.165895	105	1	0	1.619559	9.921120	-2.995888
37	6	0	-1.152759	6.971566	-3.163663	106	1	0	2.153031	6.374446	0.427829
38	6	0	-1.111487	5.979094	-2.130063	107	1	0	3.742820	8.196548	0.310437
39	6	0	-0.088448	6.029945	-1.132800	108	1	0	-0.273252	8.768186	-3.942646
40	6	0	-2.169686	6.910745	-4.165391	109	1	0	-2.162361	7.663044	-4.949093
41	6	0	-3.130816	5.945746	-4.134978	110	1	0	-3.900248	5.909492	-4.899328
42	6	0	-3.136997	4.998741	-3.077849	111	1	0	-3.925119	4.254263	-3.029136
43	6	0	-2.171642	5.019608	-2.111718	112	1	0	-2.223158	4.295756	-1.311187
44	6	0	1.597783	-7.168398	1.219144	113	1	0	1.823299	-6.913994	0.193333
45	6	0	2.309230	-8.169254	1.816714	114	1	0	3.073198	-8.692280	1.250564
46	6	0	2.071017	-8.526360	3.169761	115	1	0	2.638394	-9.331007	3.626022
47	6	0	1.146481	-7.834491	3.891790	116	1	0	0.968107	-8.070448	4.937138
48	6	0	-0.178292	-5.391177	1.327823	117	1	0	-0.591413	-6.251845	5.138596
49	6	0	0.578118	-6.448405	1.918880	118	1	0	-3.557566	-2.285104	1.976519
50	6	0	0.394520	-6.770882	3.304074	119	1	0	-2.046954	-3.514952	0.549220
51	6	0	-0.484435	-6.018845	4.082044	120	1	0	-2.171392	-4.452238	5.412217
52	6	0	-2.883297	-3.013057	2.417829	121	1	0	-3.603855	-2.688886	4.445465
53	6	0	-2.020988	-3.701703	1.612837	122	1	0	-2.153031	-6.374446	0.427829
54	6	0	-1.113432	-4.676598	2.138238	123	1	0	-3.742820	-8.196548	0.310437
55	6	0	-1.229200	-4.975968	3.534902	124	1	0	-3.477466	-10.022302	-1.372637
56	6	0	-2.129788	-4.223208	4.351096	125	1	0	-1.619559	-9.921120	-2.995888
57	6	0	-2.923732	-3.254289	3.816681	126	1	0	0.273252	-8.768186	-3.942646
58	6	0	-1.998397	-7.155744	-0.302762	127	1	0	3.900248	-5.909492	-4.899328
59	6	0	-2.899996	-8.179192	-0.372909	128	1	0	3.925119	-4.254263	-3.029136
60	6	0	-2.757340	-9.211268	-1.337144	129	1	0	2.223158	-4.295756	-1.311187
61	6	0	-1.729804	-9.158688	-2.229942	130	1	0	2.162361	-7.663044	-4.949093
62	6	0	0.088448	-6.029945	-1.132800	131	1	0	-1.567193	-4.787021	-2.174328
63	6	0	-0.871705	-7.087385	-1.180468	132	1	0	-2.955652	-3.696425	-3.825888
64	6	0	-0.777281	-8.094248	-2.196282	133	1	0	-2.929813	-1.208439	-4.073637
65	6	0	0.221154	-8.009263	-3.165895	134	1	0	-1.518371	0.147576	-2.587949
66	6	0	3.130816	-5.945746	-4.134978	135	1	0	1.659427	0.544051	1.112164
67	6	0	3.136997	-4.998741	-3.077849	136	1	0	3.226419	-0.486396	2.686879
68	6	0	2.171642	-5.019608	-2.111718	137	1	0	3.481569	-2.975496	2.744121
69	6	0	1.111487	-5.979094	-2.130063	138	1	0	2.102421	-4.387275	1.329758

No imaginary frequencies.

Zero-point correction= 1.108919 (Hartree/Particle)
Thermal correction to Energy= 1.171046
Thermal correction to Enthalpy= 1.171990
Thermal correction to Gibbs Free Energy= 1.007141

Sum of electronic and zero-point Energies= -3306.382310
Sum of electronic and thermal Energies= -3306.320183
Sum of electronic and thermal Enthalpies= -3306.319239
Sum of electronic and thermal Free Energies= -3306.484088

Table S11. Cartesian coordinate of **A-CH** TS (ωB97X-D/6-31G**)

Center Number	Atomic Number	Atomic Type	X	Y	Z	Center Number	Atomic Number	Atomic Type	X	Y	Z
1	6	0	-1.794491	0.152339	2.018223	70	6	0	6.268366	2.467059	0.953185
2	6	0	-0.459424	-0.280852	2.204602	71	6	0	7.642151	2.593554	1.337711
3	6	0	-0.065287	-1.696036	1.863872	72	6	0	3.824550	-0.314172	-0.118268
4	6	0	-1.194000	-2.553208	1.368965	73	6	0	4.299785	-0.290457	3.155543
5	6	0	-2.506365	-2.060304	1.128568	74	6	0	4.065239	-0.174802	4.523609
6	6	0	-2.749897	-0.598995	1.178677	75	6	0	2.918500	-0.711790	5.095640
7	6	0	-2.217382	1.366012	2.578841	76	6	0	1.989938	-1.350024	4.284949
8	6	0	-1.326188	2.265383	3.131796	77	6	0	2.033336	-4.588014	1.467154
9	6	0	0.027179	1.961726	3.076341	78	6	0	2.900579	-5.393831	0.733944
10	6	0	0.439226	0.716528	2.632874	79	6	0	3.831310	-4.821723	-0.122782
11	6	0	-1.003780	-3.931143	1.210566	80	6	0	3.960566	-3.438174	-0.158279
12	6	0	-2.000433	-4.809363	0.828655	81	6	0	3.481915	-1.201992	0.847758
13	6	0	-3.283126	-4.325590	0.630197	82	6	0	3.381099	-0.932405	2.324109
14	6	0	-3.516773	-2.971688	0.787671	83	6	0	2.206743	-1.439660	2.912430
15	6	0	-3.658795	0.071021	0.408225	84	6	0	1.224951	-2.148899	2.048125
16	6	0	-6.098585	-1.055450	-3.811107	85	6	0	2.053560	-3.198835	1.339077
17	6	0	-6.091816	-0.972518	-2.382869	86	6	0	3.118626	-2.628286	0.600058
18	6	0	-4.863741	-0.679310	-1.702285	87	1	0	-3.270142	1.610734	2.560330
19	6	0	-3.680535	-0.572201	-2.496266	88	1	0	-1.680006	3.201173	3.551173
20	6	0	-3.720222	-0.671645	-3.854576	89	1	0	0.776737	2.681119	3.388328
21	6	0	-4.952451	-0.899220	-4.529326	90	1	0	1.499927	0.548493	2.615318
22	6	0	-7.253530	-1.177292	-1.643479	91	1	0	-0.034946	-4.341053	1.388536
23	6	0	-7.228389	-1.177150	-0.251146	92	1	0	-1.771853	-5.864082	0.714665
24	6	0	-6.005772	-0.882918	0.438590	93	1	0	-4.101686	-4.986451	0.365073
25	6	0	-4.842339	-0.550008	-0.297067	94	1	0	-4.522713	-2.620917	0.647833
26	6	0	-8.405618	-1.481008	0.504067	95	1	0	-7.042654	-1.258026	-4.309041
27	6	0	-8.373578	-1.552976	1.862440	96	1	0	-2.730335	-0.434720	-2.002661
28	6	0	-7.147896	-1.330401	2.551459	97	1	0	-2.799504	-0.581027	-4.422737
29	6	0	-6.015315	-1.003400	1.868371	98	1	0	-4.969719	-0.966254	-5.612387
30	6	0	-6.655398	4.289076	1.165600	99	1	0	-8.189435	-1.374394	-2.160520
31	6	0	-5.732866	4.646900	0.230399	100	1	0	-9.326847	-1.671432	-0.039368
32	6	0	-4.675522	3.760492	-0.144720	101	1	0	-9.270478	-1.796052	2.422673
33	6	0	-4.603306	2.453145	0.439609	102	1	0	-7.115206	-1.429203	3.631767
34	6	0	-5.571736	2.143611	1.444055	103	1	0	-5.085873	-0.858578	2.408729
35	6	0	-6.556441	3.018686	1.795204	104	1	0	-7.450760	4.972082	1.445853
36	6	0	-3.717718	4.150000	-1.075800	105	1	0	-5.774201	5.623085	-0.244772
37	6	0	-2.656976	3.314259	-1.403779	106	1	0	-5.505725	1.199396	1.962131
38	6	0	-2.554013	2.012870	-0.801288	107	1	0	-7.266134	2.743441	2.568880
39	6	0	-3.575343	1.552764	0.066882	108	1	0	-3.797167	5.124994	-1.550365
40	6	0	-1.683438	3.730359	-2.368409	109	1	0	-1.797196	4.714548	-2.814341
41	6	0	-0.654762	2.917717	-2.727250	110	1	0	0.074261	3.236756	-3.464425
42	6	0	-0.514380	1.649169	-2.099964	111	1	0	0.331362	1.019698	-2.351286
43	6	0	-1.407559	1.231848	-1.159684	112	1	0	-1.243965	0.282877	-0.669677
44	6	0	5.990302	-0.146230	-2.217792	113	1	0	6.290370	-0.191017	-1.188119
45	6	0	6.955225	0.076934	-3.154575	114	1	0	7.981136	0.226556	-2.832973
46	6	0	6.637899	0.101763	-4.538472	115	1	0	7.412985	0.294145	-5.273073
47	6	0	5.358971	-0.150847	-4.925831	116	1	0	5.090608	-0.176732	-5.978249
48	6	0	3.587121	-0.558513	-1.599640	117	1	0	2.853122	-0.832668	-5.462303
49	6	0	4.618114	-0.356666	-2.559874	118	1	0	-0.796636	-1.956870	-1.024767
50	6	0	4.330314	-0.411633	-3.966733	119	1	0	1.264774	-1.048744	-0.166411
51	6	0	3.057978	-0.763649	-4.396724	120	1	0	0.655284	-1.630668	-5.040123
52	6	0	0.005155	-1.712859	-1.711514	121	1	0	-1.154537	-2.263975	-3.464094
53	6	0	1.181031	-1.220621	-1.226723	122	1	0	1.684676	1.132052	-0.177988
54	6	0	2.298444	-0.937330	-2.075114	123	1	0	0.270989	3.097340	-0.018400
55	6	0	2.056122	-1.073700	-3.487719	124	1	0	1.244404	5.345097	0.428665
56	6	0	0.789709	-1.539626	-3.965922	125	1	0	3.675828	5.577219	0.806420
57	6	0	-0.202907	-1.885980	-3.105053	126	1	0	5.864324	4.570582	1.065021
58	6	0	2.137378	2.094514	0.023969	127	1	0	9.471619	1.608686	1.798910
59	6	0	1.341654	3.199022	0.110171	128	1	0	8.511830	-0.673706	1.455853
60	6	0	1.896413	4.478879	0.381950	129	1	0	6.191432	-0.957657	0.777463
61	6	0	3.235569	4.608072	0.588884	130	1	0	8.033885	3.591995	1.510803
62	6	0	4.379699	1.034503	0.274718	131	1	0	5.220741	0.106525	2.760656
63	6	0	3.546258	2.174033	0.257396	132	1	0	4.802334	0.324984	5.143733
64	6	0	4.094465	3.466183	0.560916	133	1	0	2.748615	-0.635508	6.164667
65	6	0	5.448879	3.586810	0.860410	134	1	0	1.073491	-1.757461	4.698941
66	6	0	8.434425	1.500097	1.499463	135	1	0	1.360838	-5.063281	2.171857
67	6	0	7.889292	0.200285	1.293245	136	1	0	2.848263	-6.471804	0.846245
68	6	0	6.593464	0.042236	0.904412	137	1	0	4.494875	-5.445705	-0.712079
69	6	0	5.723323	1.167067	0.690167	138	1	0	4.748613	-2.974712	-0.743703

One imaginary frequency.

Zero-point correction= 1.114695 (Hartree/Particle)
Thermal correction to Energy= 1.174524
Thermal correction to Enthalpy= 1.175469
Thermal correction to Gibbs Free Energy= 1.022999

Sum of electronic and zero-point Energies= -3306.360936
Sum of electronic and thermal Energies= -3306.301107
Sum of electronic and thermal Enthalpies= -3306.300163
Sum of electronic and thermal Free Energies= -3306.452632

Table S12. Cartesian coordinate of **A-CH** dication state(ωB97X-D/6-31G**)

Center Number	Atomic Number	Atomic Type	Coordinates (Angstroms) X	Y	Z
1	6	0	0.660637	2.948033	-1.351280
2	6	0	0.701593	1.514041	-1.416922
3	6	0	-0.059239	0.744086	-0.521319
4	6	0	-0.924189	1.350557	0.404579
5	6	0	-0.985742	2.776946	0.480979
6	6	0	-0.143546	3.571231	-0.350030
7	6	0	1.542958	3.660393	-2.222423
8	6	0	2.340352	3.013942	-3.122098
9	6	0	2.340352	1.599297	-3.213330
10	6	0	1.552830	0.874360	-2.373320
11	6	0	-1.748131	0.563846	1.270813
12	6	0	-2.629700	1.152578	2.124996
13	6	0	-2.757929	2.565177	2.142297
14	6	0	-1.969311	3.346303	1.348038
15	6	0	-0.019374	5.009180	-0.077638
16	6	0	-1.212869	7.816881	3.868798
17	6	0	-0.461428	6.762110	3.268217
18	6	0	-0.568612	6.497642	1.862475
19	6	0	-1.520796	7.277631	1.145270
20	6	0	-2.233993	8.276398	1.753743
21	6	0	-2.072123	8.571767	3.128881
22	6	0	0.354212	5.961758	4.065758
23	6	0	1.122540	4.921314	3.544911
24	6	0	1.074612	4.649678	2.142536
25	6	0	0.186294	5.405690	1.301188
26	6	0	1.984217	4.170682	4.399500
27	6	0	2.818189	3.220698	3.889149
28	6	0	2.848271	3.012309	2.488205
29	6	0	2.018014	3.703127	1.645812
30	6	0	2.646514	9.275542	-1.416866
31	6	0	1.672708	9.103351	-2.355322
32	6	0	0.767589	8.003973	-2.272767
33	6	0	0.837056	7.079121	-1.180832
34	6	0	1.906834	7.265520	-0.263869
35	6	0	2.767949	8.327060	-0.374526
36	6	0	-0.168790	7.806719	-3.286587
37	6	0	-1.074216	6.745480	-3.282010
38	6	0	-1.064812	5.816311	-2.194287
39	6	0	-0.090394	5.969845	-1.139584
40	6	0	-2.030240	6.625918	-4.332844
41	6	0	-2.981167	5.648605	-4.300595
42	6	0	-3.026037	4.771938	-3.191580
43	6	0	-2.112894	4.856416	-2.171929
44	6	0	1.520796	-7.277631	1.145270
45	6	0	2.233993	-8.276398	1.753743
46	6	0	2.072123	-8.571767	3.128881
47	6	0	1.212869	-7.816881	3.868798
48	6	0	-0.186294	-5.405690	1.301188
49	6	0	0.568612	-6.497642	1.862475
50	6	0	0.461428	-6.762110	3.268217
51	6	0	-0.354212	-5.961758	4.065758
52	6	0	-2.848271	-3.012309	2.488205
53	6	0	-2.018014	-3.703127	1.645812
54	6	0	-1.074612	-4.649678	2.142536
55	6	0	-1.122540	-4.921314	3.544911
56	6	0	-1.984217	-4.170682	4.399500
57	6	0	-2.818189	-3.220698	3.889149
58	6	0	-1.906834	-7.265520	-0.263869
59	6	0	-2.767949	-8.327060	-0.374526
60	6	0	-2.646514	-9.275542	-1.416866
61	6	0	-1.672708	-9.103351	-2.355322
62	6	0	0.090394	-5.969845	-1.139584
63	6	0	-0.837056	-7.079121	-1.180832
64	6	0	-0.767589	-8.003973	-2.272767
65	6	0	0.168790	-7.806719	-3.286587
66	6	0	2.981167	-5.648605	-4.300595
67	6	0	3.026037	-4.771938	-3.191580
68	6	0	2.112894	-4.856416	-2.171929
69	6	0	1.064812	-5.816311	-2.194287
70	6	0	1.074216	-6.745480	-3.282010
71	6	0	2.030240	-6.625918	-4.332844
72	6	0	0.019374	-5.009180	-0.077638
73	6	0	-1.542958	-3.660393	-2.222423
74	6	0	-2.340352	-3.013942	-3.122098
75	6	0	-2.340352	-1.599297	-3.213330
76	6	0	-1.552830	-0.874360	-2.373320
77	6	0	1.748131	-0.563846	1.270813
78	6	0	2.629700	-1.152578	2.124996
79	6	0	2.757929	-2.565177	2.142297
80	6	0	1.969311	-3.346303	1.348038
81	6	0	0.143546	-3.571231	-0.350030
82	6	0	-0.660637	-2.948033	-1.351280
83	6	0	-0.701593	-1.514041	-1.416922
84	6	0	0.059239	-0.744086	-0.521319
85	6	0	0.924189	-1.350557	0.404579
86	6	0	0.985742	-2.776946	0.480979
87	1	0	1.592019	4.737876	-2.176863
88	1	0	2.989491	3.592657	-3.770428
89	1	0	2.977390	1.103070	-3.936800
90	1	0	1.562064	-0.208562	-2.415990
91	1	0	-1.668855	-0.516671	1.230892
92	1	0	-3.250708	0.546017	2.775258
93	1	0	-3.498649	3.029708	2.784234
94	1	0	-2.114403	4.418246	1.363909
95	1	0	-1.090007	7.995150	4.932497
96	1	0	-1.699929	7.091882	0.096649
97	1	0	-2.943903	8.849669	1.166807
98	1	0	-2.641789	9.373604	3.584181
99	1	0	0.404540	6.166884	5.132360
100	1	0	1.976692	4.391287	5.462344
101	1	0	3.480731	2.659290	4.538130
102	1	0	3.561803	2.310674	2.066732
103	1	0	2.115935	3.541293	0.582138
104	1	0	3.335543	10.109972	-1.477040
105	1	0	1.575850	9.790227	-3.190220
106	1	0	2.064489	6.563438	0.541327
107	1	0	3.567145	8.434350	0.351301
108	1	0	-0.197677	8.512676	-4.113292
109	1	0	-1.994875	7.341810	-5.148015
110	1	0	-3.712625	5.559453	-5.095460
111	1	0	-3.811086	4.024991	-3.135787
112	1	0	-2.217278	4.183152	-1.333993
113	1	0	1.699929	-7.091882	0.096649
114	1	0	2.943903	-8.849669	1.166807
115	1	0	2.641789	-9.373604	3.584181
116	1	0	1.090007	-7.995150	4.932497
117	1	0	-0.404540	-6.166884	5.132360
118	1	0	-3.561803	-2.310674	2.066732
119	1	0	-2.115935	-3.541293	0.582138
120	1	0	-1.976692	-4.391287	5.462344
121	1	0	-3.480731	-2.659290	4.538130
122	1	0	-2.064489	-6.563438	0.541327
123	1	0	-3.567145	-8.434350	0.351301
124	1	0	-3.335543	-10.109972	-1.477040
125	1	0	-1.575850	-9.790227	-3.190220
126	1	0	0.197677	-8.512676	-4.113292
127	1	0	3.712625	-5.559453	-5.095460
128	1	0	3.811086	-4.024991	-3.135787
129	1	0	2.217278	-4.183152	-1.333993
130	1	0	1.994875	-7.341810	-5.148015
131	1	0	-1.592019	-4.737876	-2.176863
132	1	0	-2.989491	-3.592657	-3.770428
133	1	0	-2.977390	-1.103070	-3.936800
134	1	0	-1.562064	0.208562	-2.415990
135	1	0	1.668855	0.516671	1.230892
136	1	0	3.250708	-0.546017	2.775258
137	1	0	3.498649	-3.029708	2.784234
138	1	0	2.114403	-4.418246	1.363909

No imaginary frequencies.

Zero-point correction= 1.114386 (Hartree/Particle)

Thermal correction to Energy= 1.176475

Thermal correction to Enthalpy= 1.177419

Thermal correction to Gibbs Free Energy= 1.012026

Sum of electronic and zero-point Energies= -3305.919840

Sum of electronic and thermal Energies= -3305.857751

Sum of electronic and thermal Enthalpies= -3305.856807

Sum of electronic and thermal Free Energies= -3306.022200
